# Supplementary figures and images for: COVID RADAR app: Description and validation of population surveillance of symptoms and behavior in relation to COVID-19 (part 1 of 2)
Source: PLoS One. 2021 Jun 30;16(6):e0253566. doi: 10.1371/journal.pone.0253566 (PMC8244909; doi:10.1371/journal.pone.0253566)

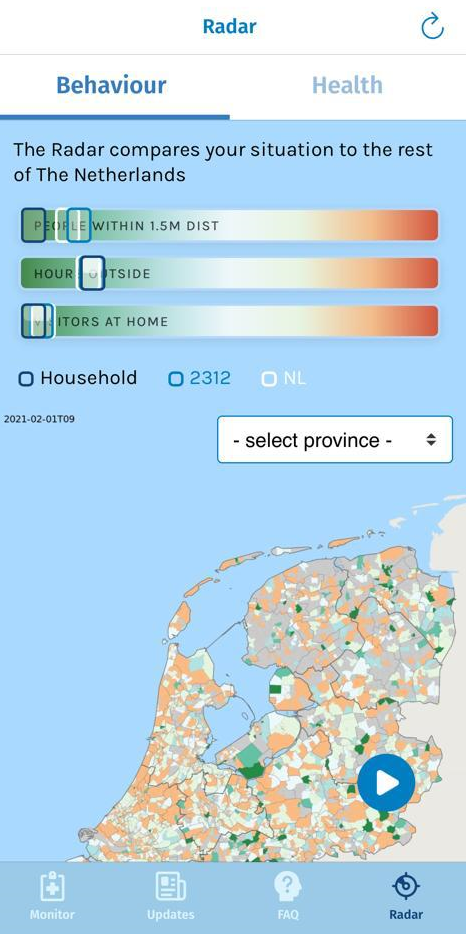

Supplement: S1 Fig — “Reprinted from “urlmaps.com” under the Creative Commons Attribution License, with permission from “i-mapping”, original copyright 2021”. (TIF) [file pone.0253566.s001.tif]

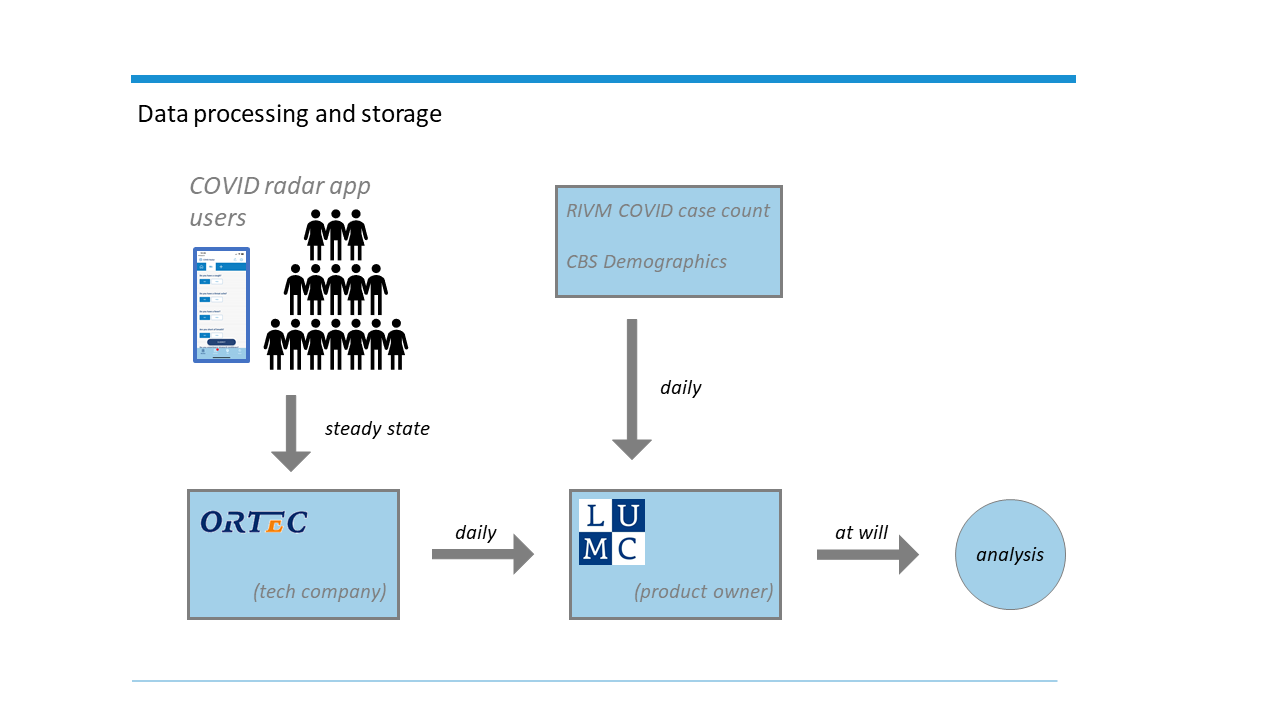

Supplement: S2 Fig — (TIF) [file pone.0253566.s002.tif]

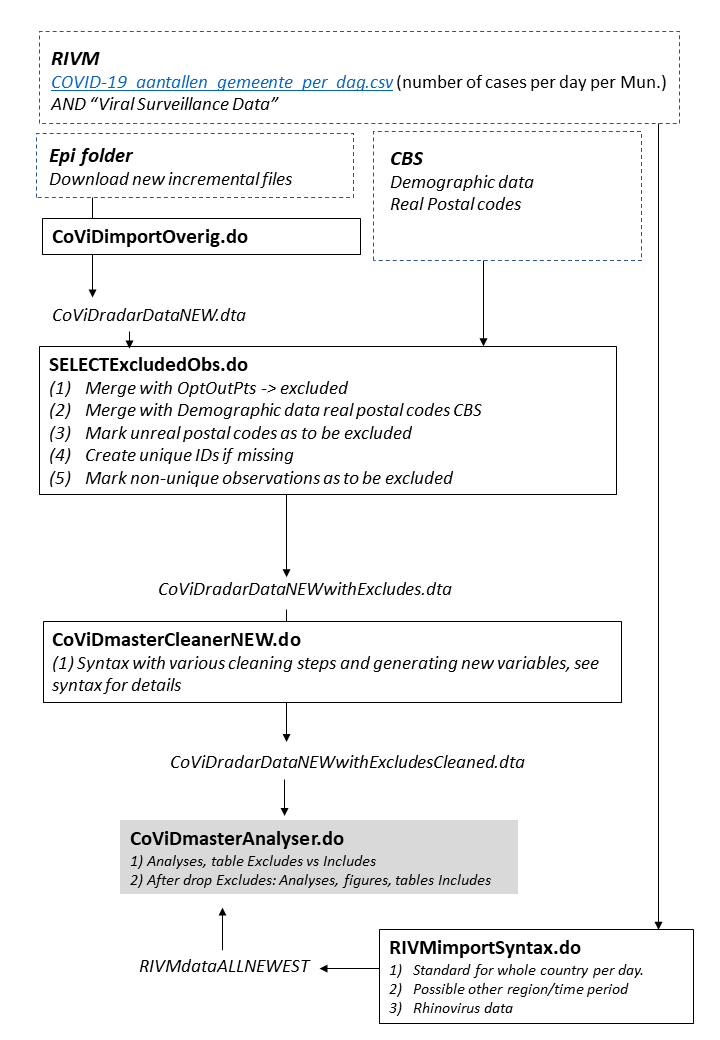

Supplement: S3 Fig — (TIF) [file pone.0253566.s003.tif]

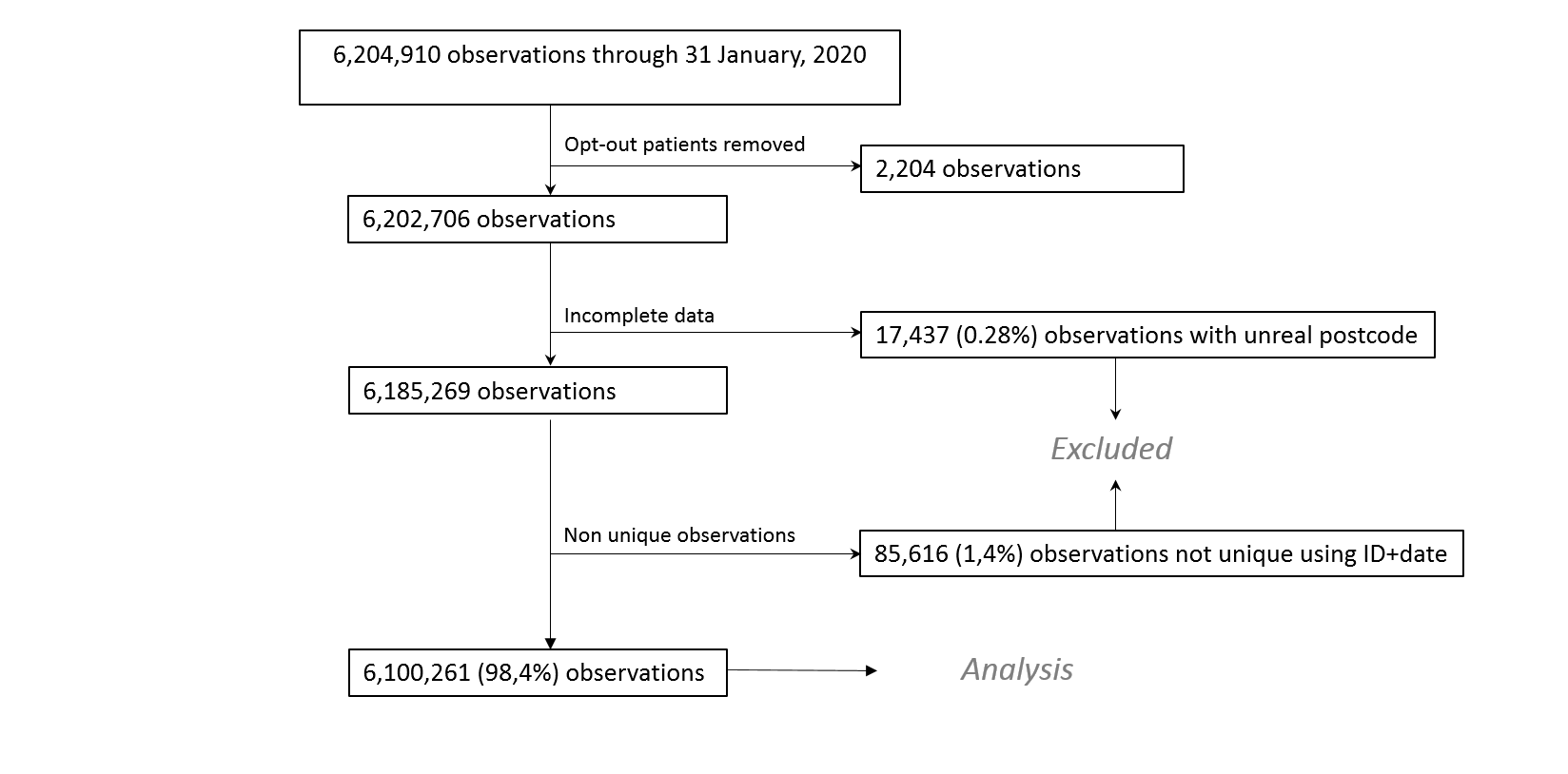

Supplement: S4 Fig — (TIF) [file pone.0253566.s004.tif]

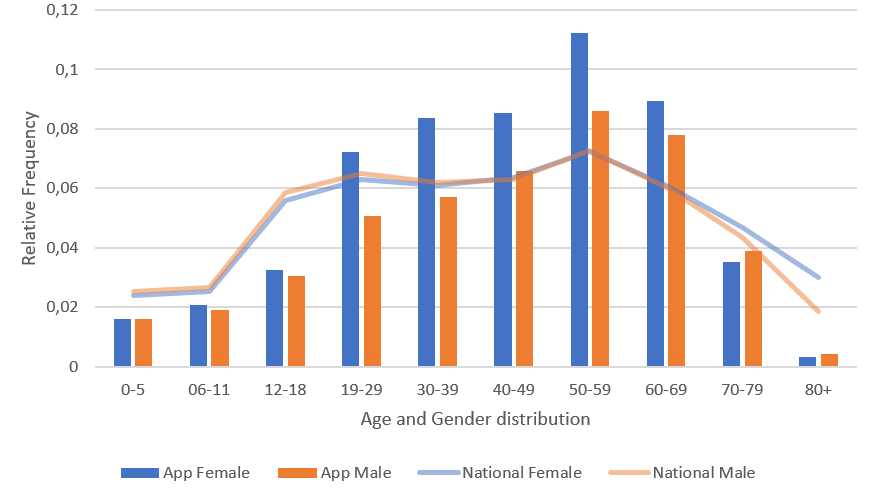

Supplement: S5 Fig — (TIF) [file pone.0253566.s005.tif]

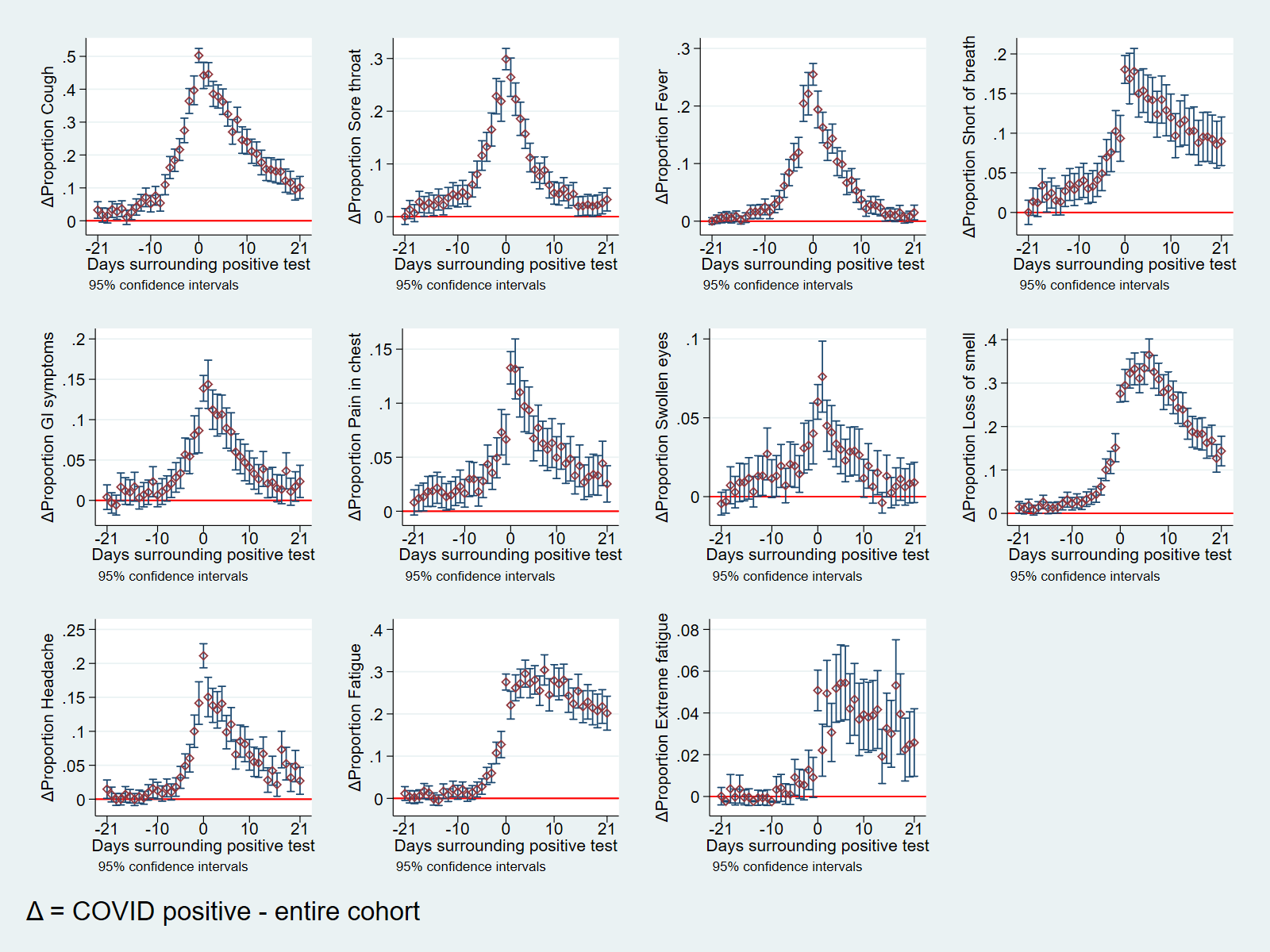

Supplement: S6 Fig — (TIF) [file pone.0253566.s006.tif]

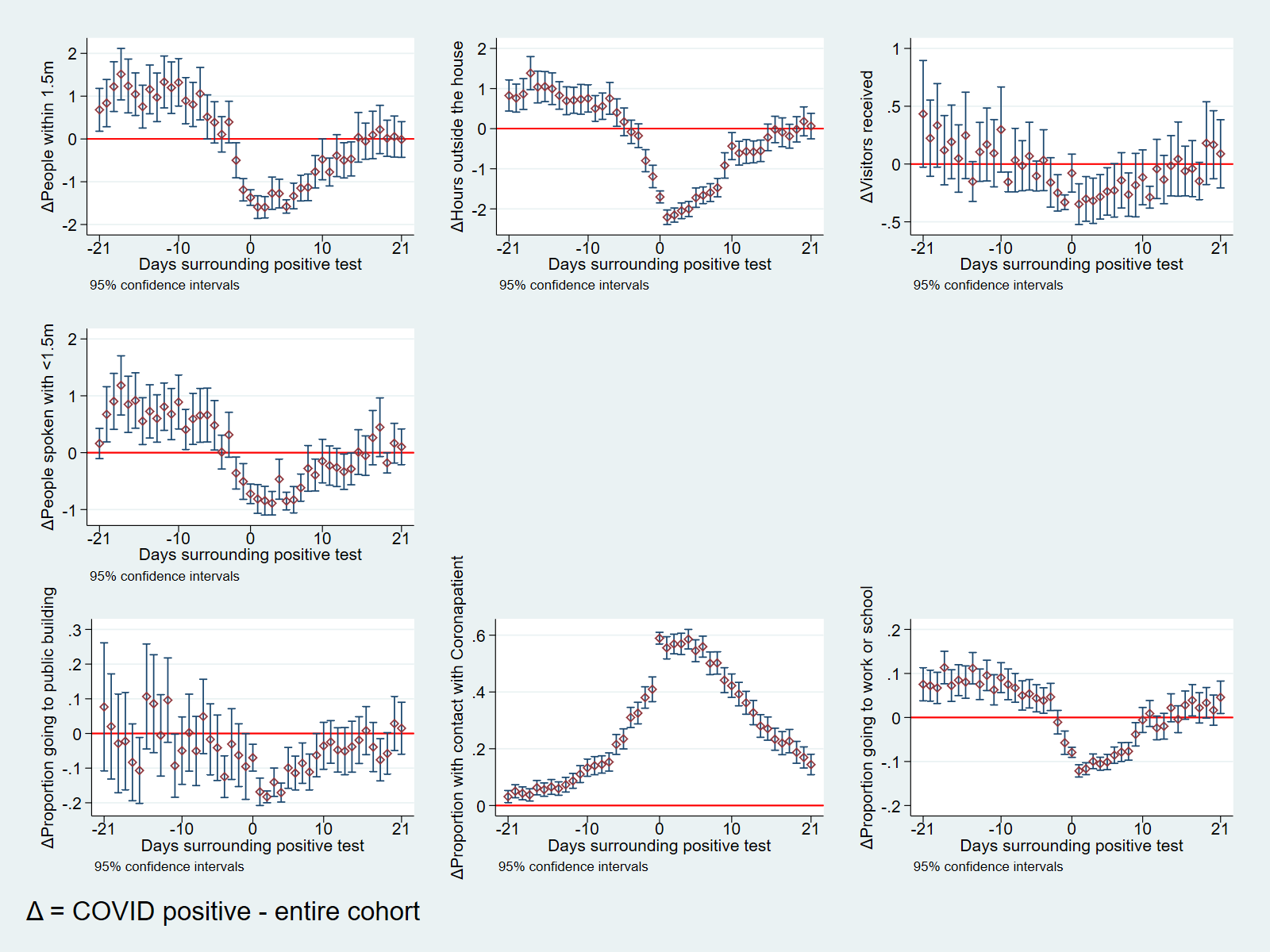

Supplement: S7 Fig — (TIF) [file pone.0253566.s007.tif]

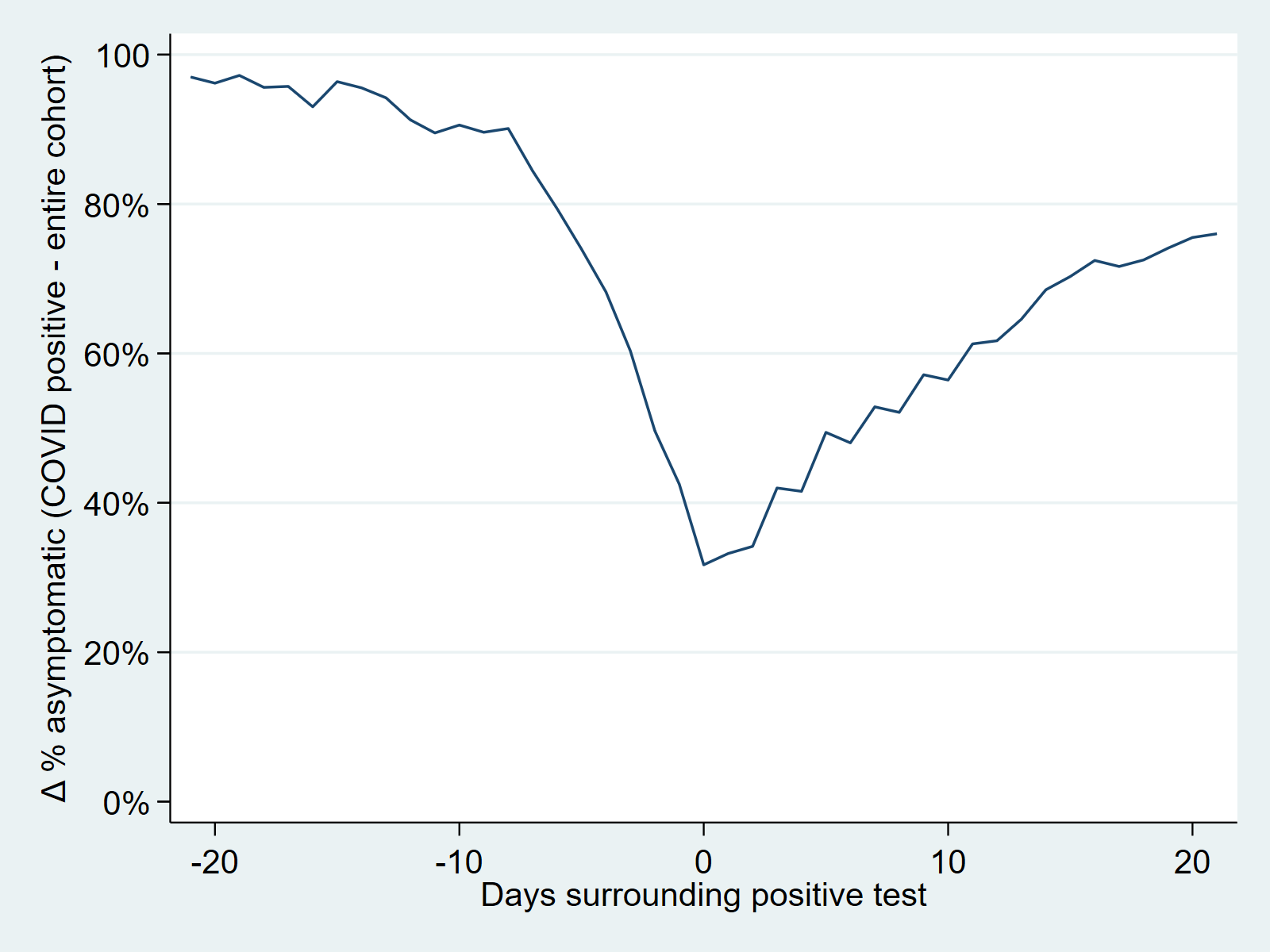

Supplement: S8 Fig — (TIF) [file pone.0253566.s008.tif]

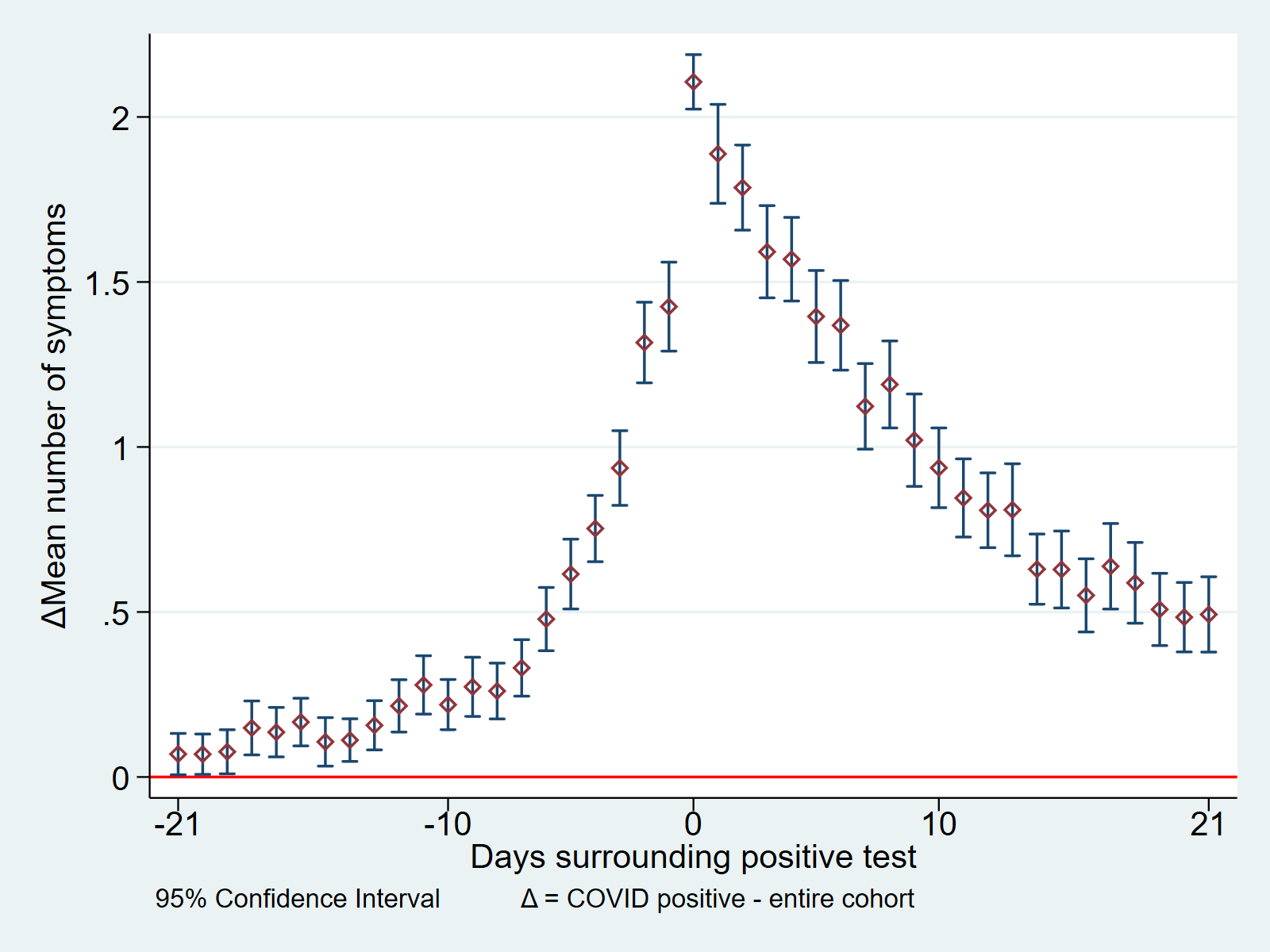

Supplement: S9 Fig — (TIF) [file pone.0253566.s009.tif]

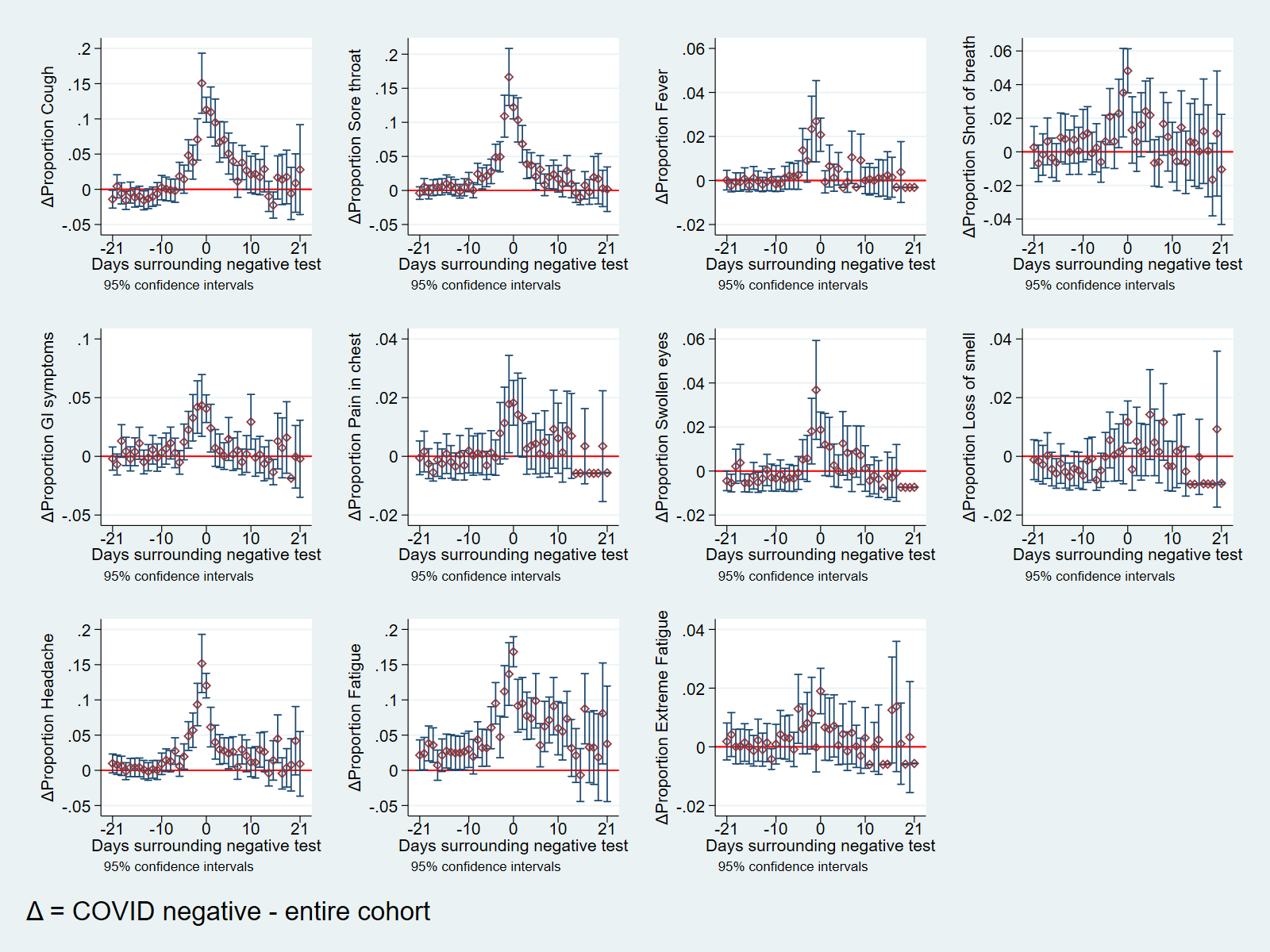

Supplement: S10 Fig — (TIF) [file pone.0253566.s010.tif]

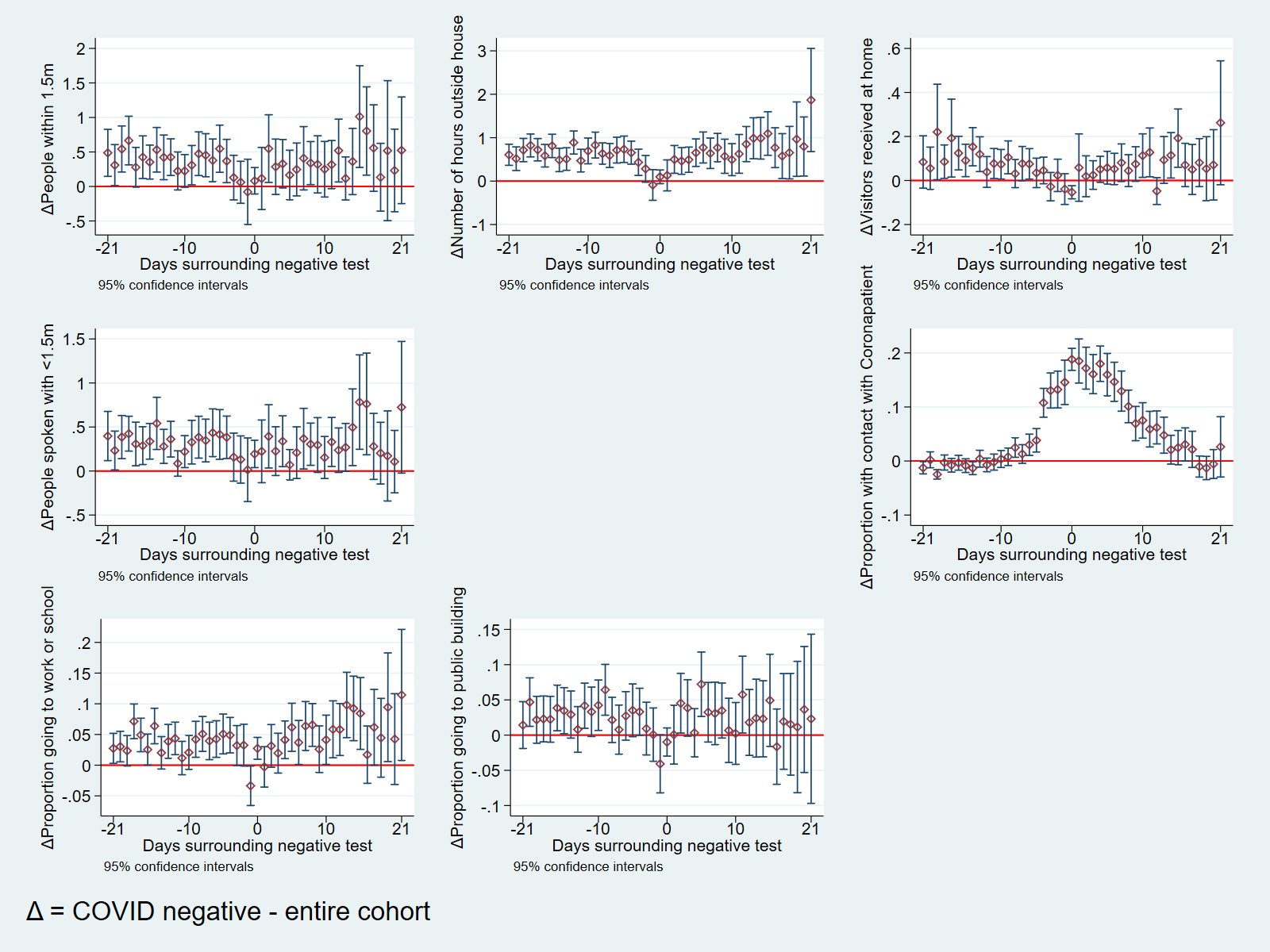

Supplement: S11 Fig — (TIF) [file pone.0253566.s011.tif]

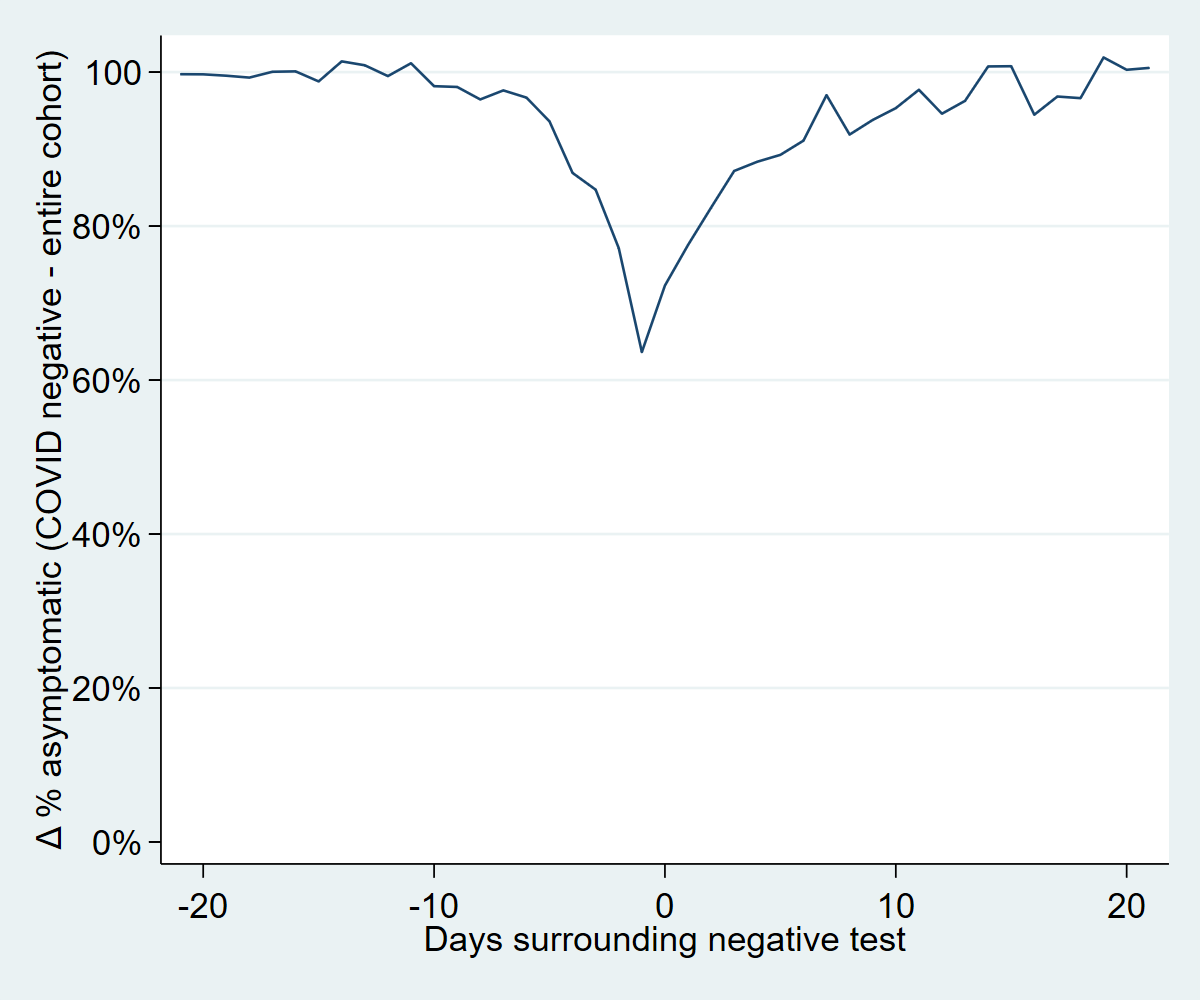

Supplement: S12 Fig — (TIF) [file pone.0253566.s012.tif]

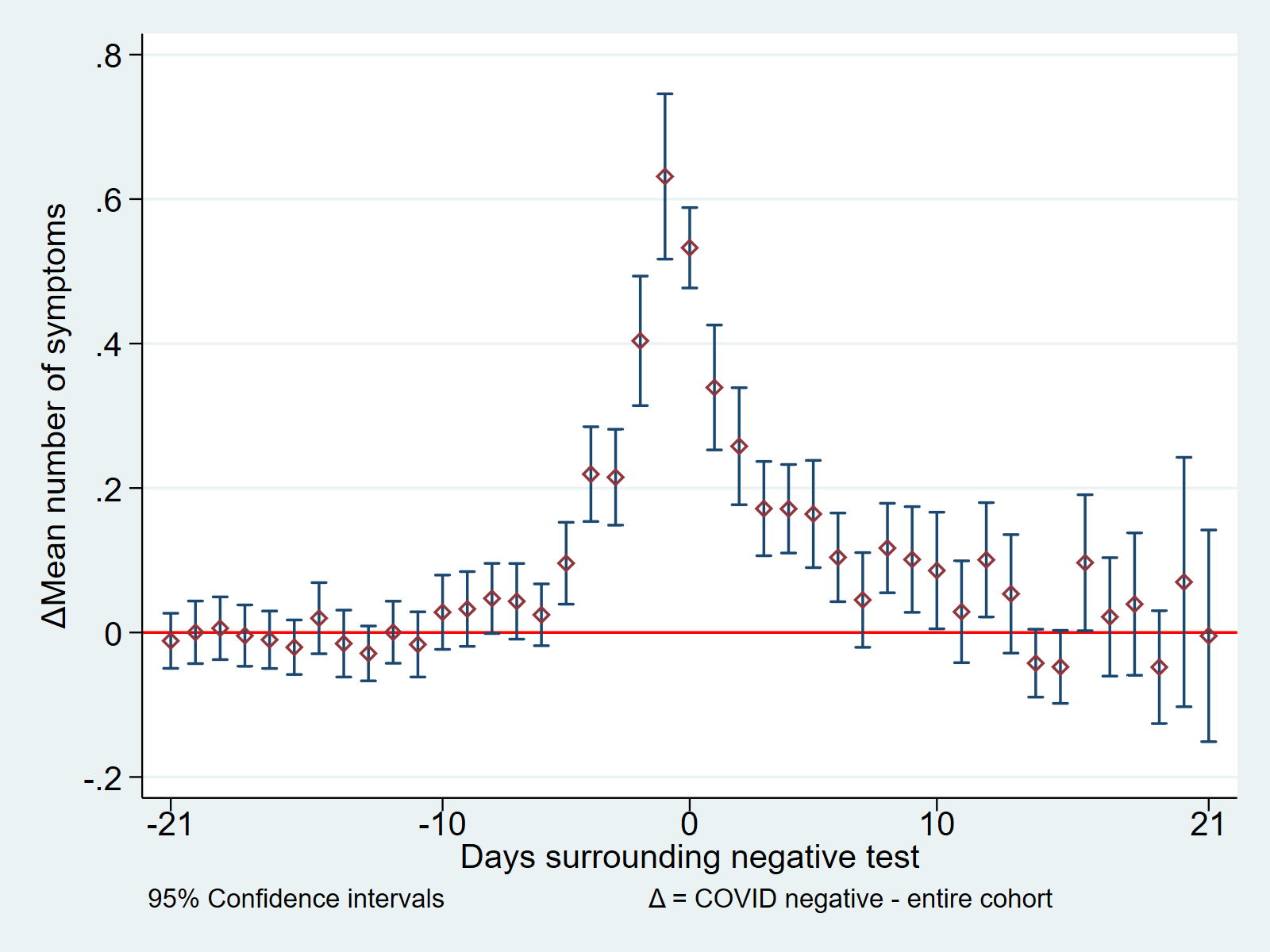

Supplement: S13 Fig — (TIF) [file pone.0253566.s013.tif]

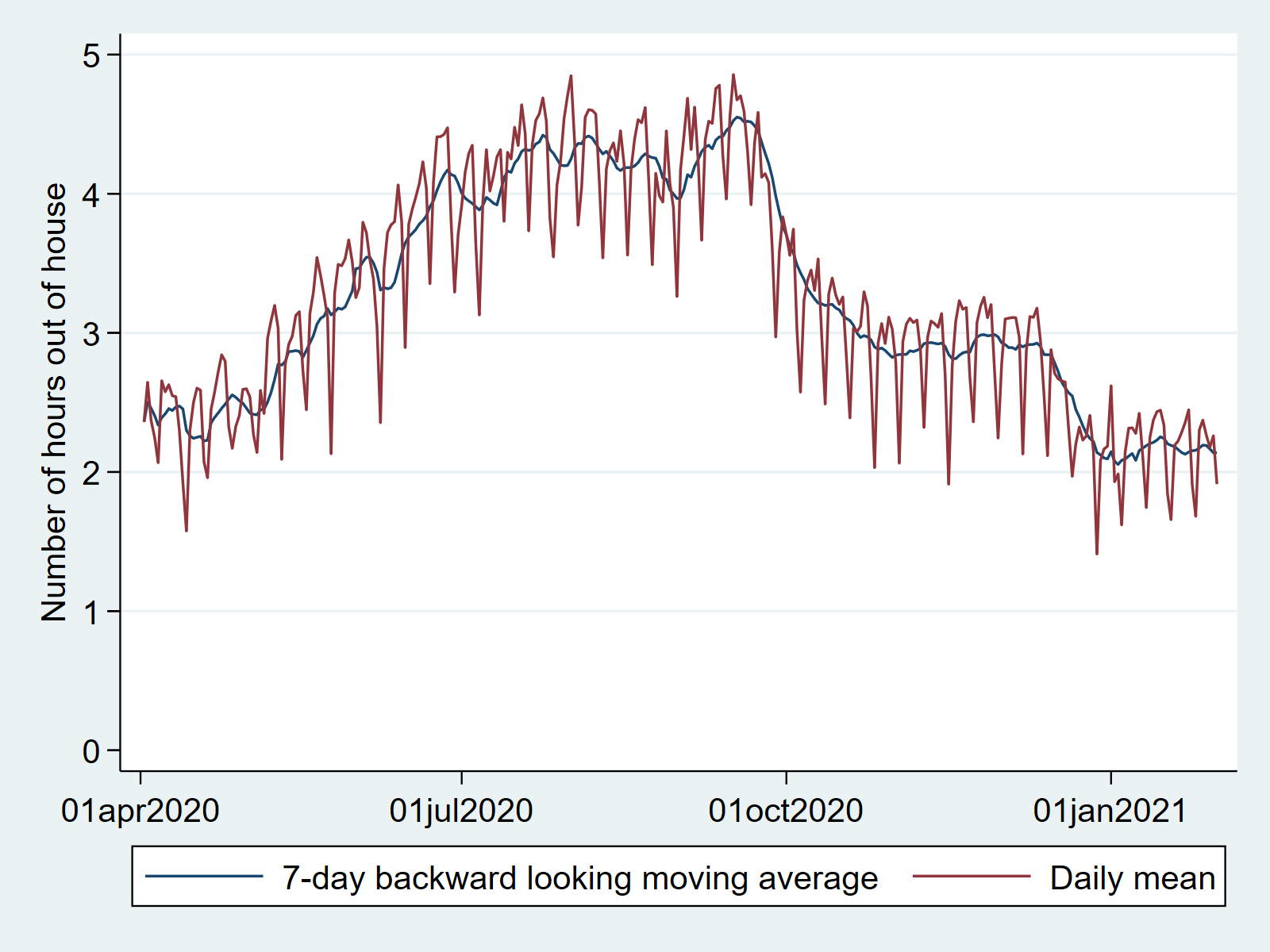

Supplement: S14 Fig — (TIF) [file pone.0253566.s014.tif]

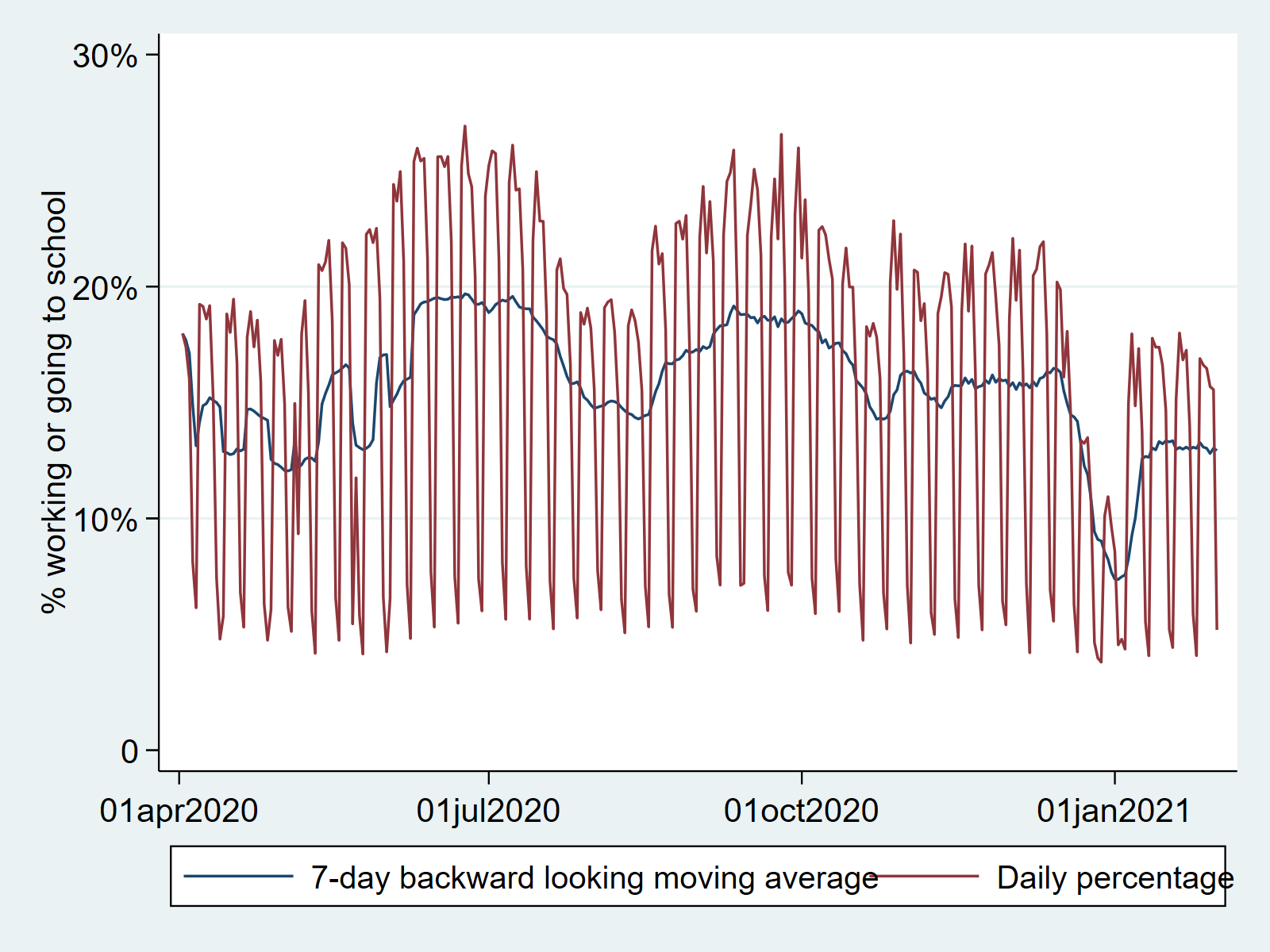

Supplement: S15 Fig — (TIF) [file pone.0253566.s015.tif]

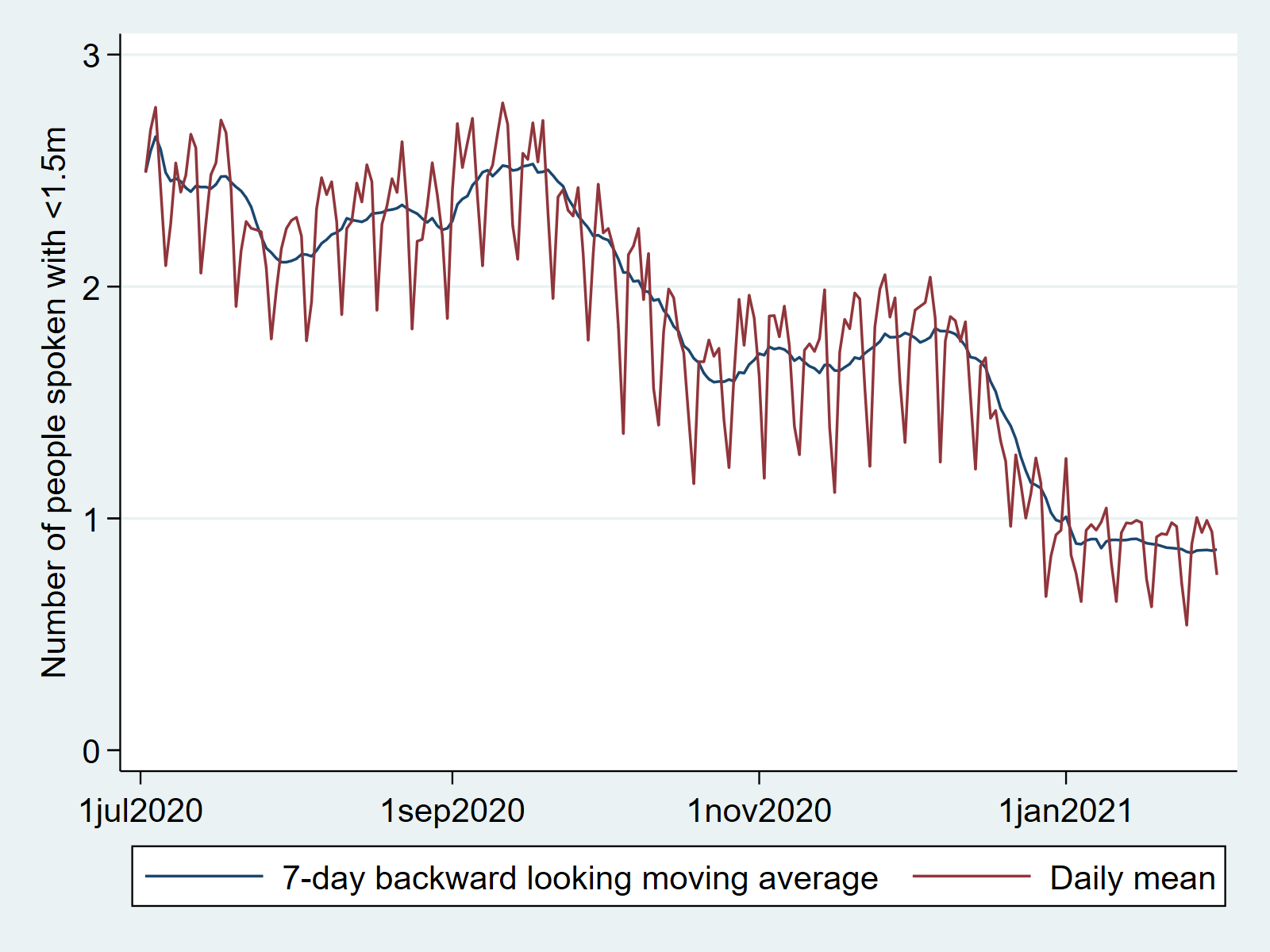

Supplement: S16 Fig — (TIF) [file pone.0253566.s016.tif]

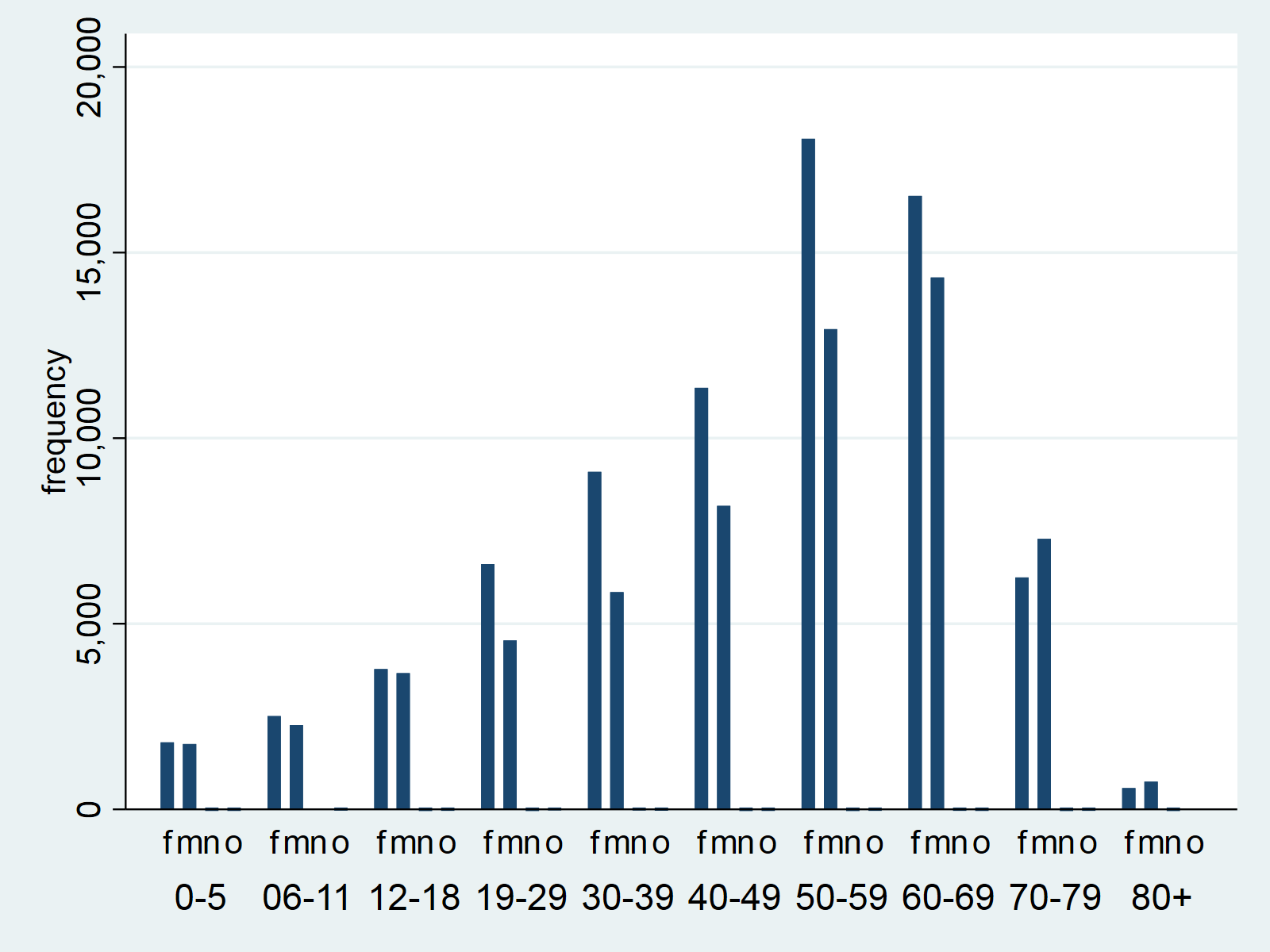

Supplement: S7 File — (ZIP) [file pone.0253566.s023.zip › sensitivity/Loyalagegenderobs.tif]

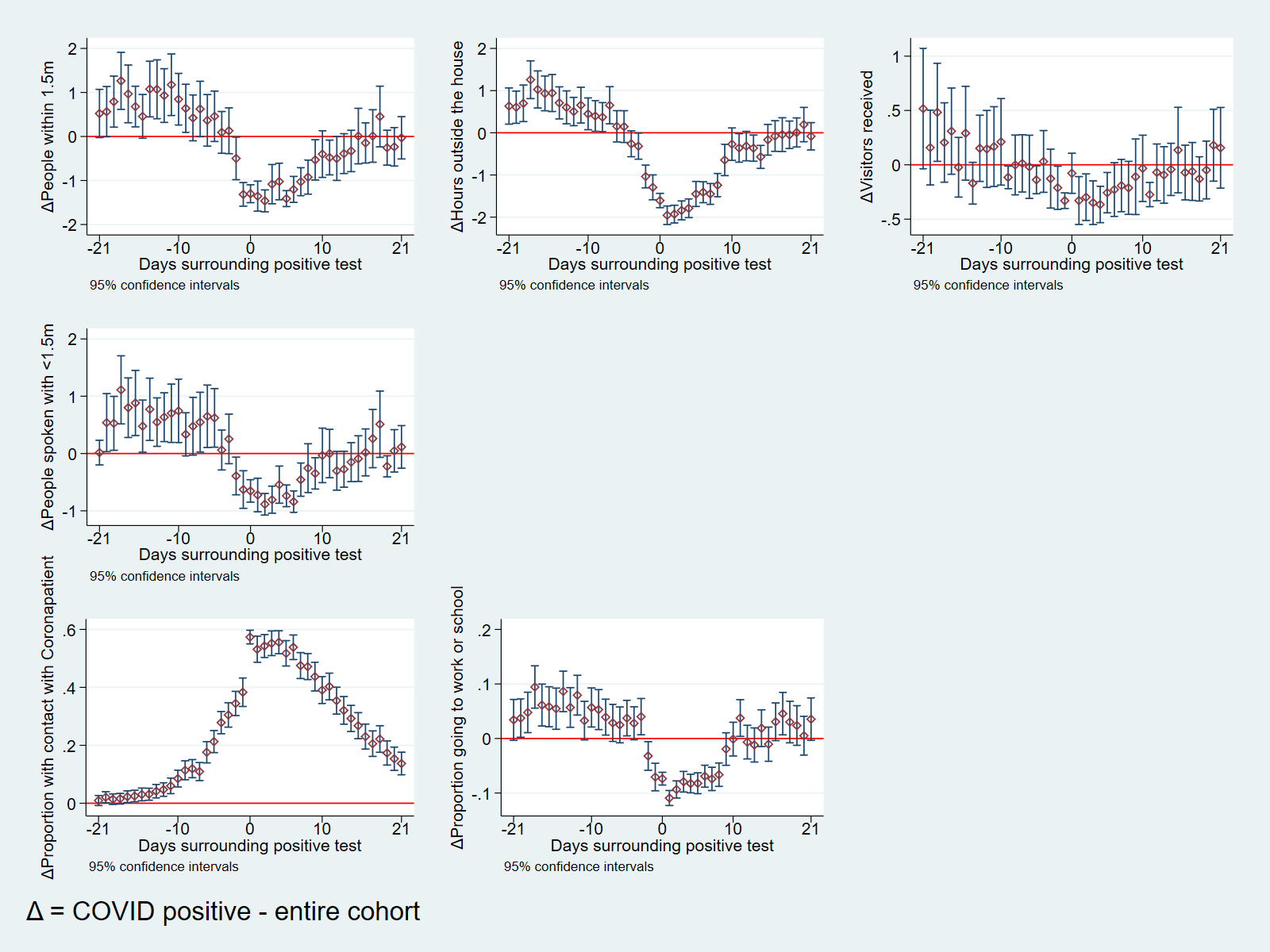

Supplement: S7 File — (ZIP) [file pone.0253566.s023.zip › sensitivity/noHCOROS19S25Behave.tif]

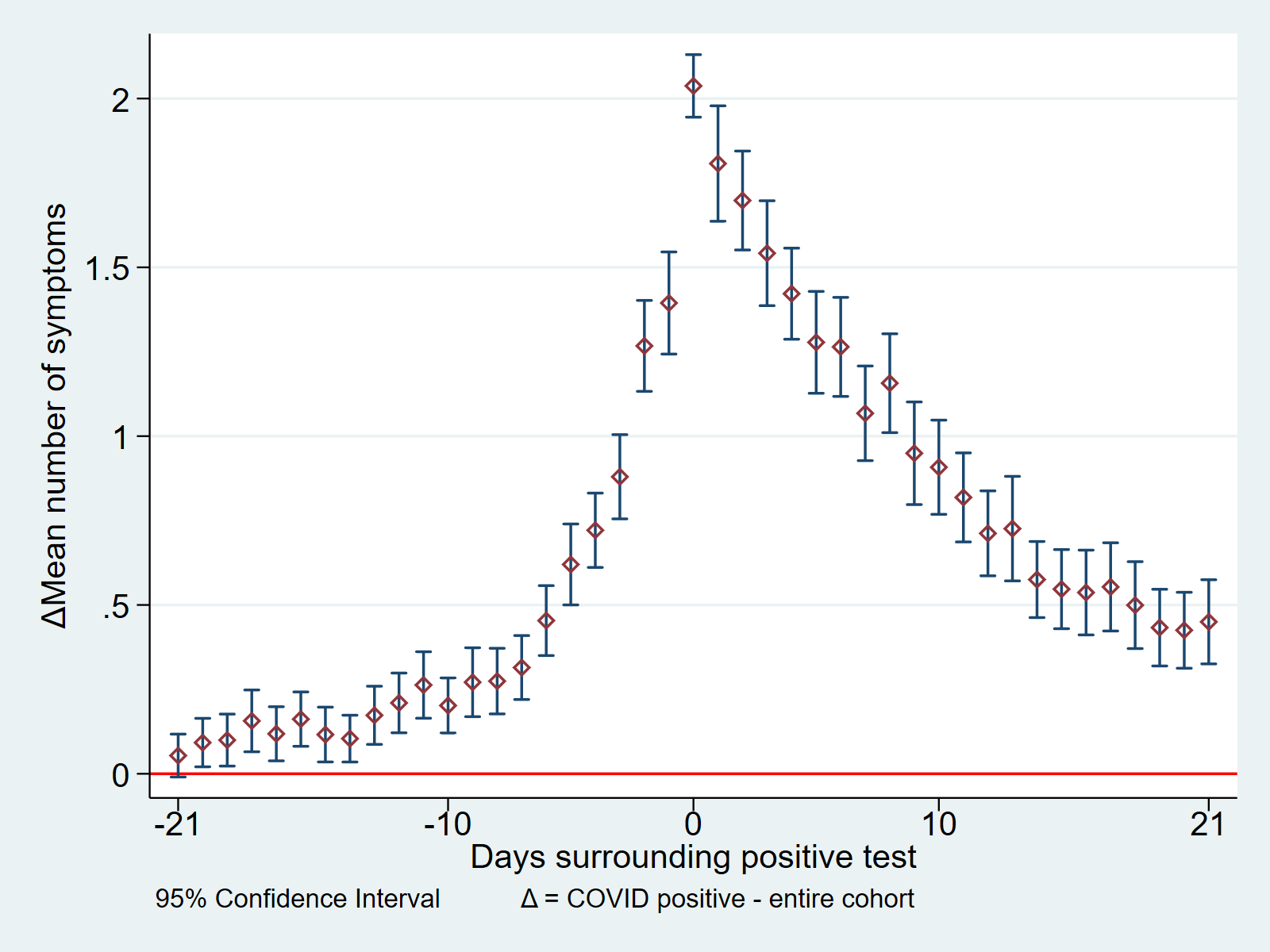

Supplement: S7 File — (ZIP) [file pone.0253566.s023.zip › sensitivity/noHCPROaantalsymp.tif]

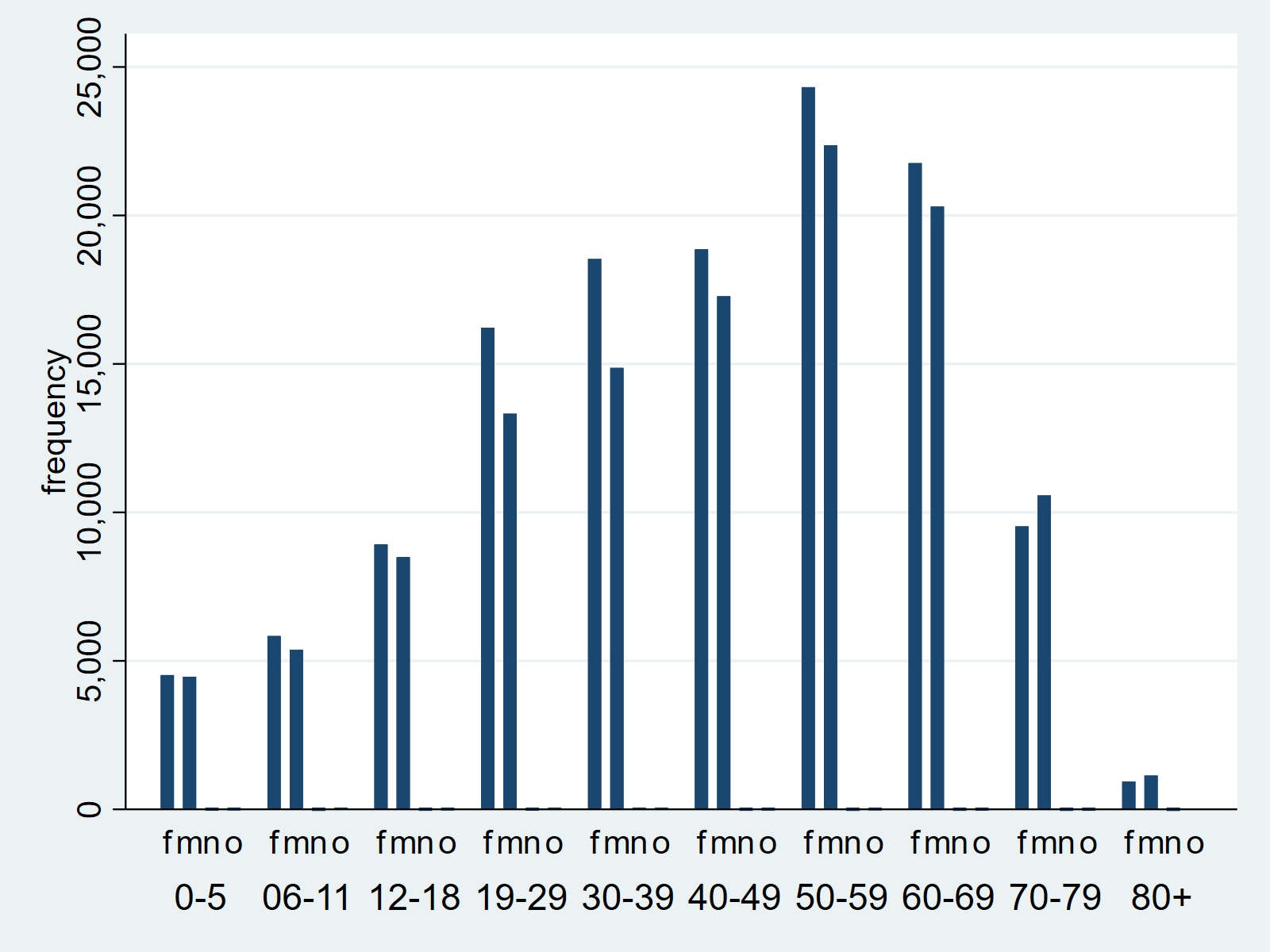

Supplement: S7 File — (ZIP) [file pone.0253566.s023.zip › sensitivity/noHCPROagegenderobs.tif]

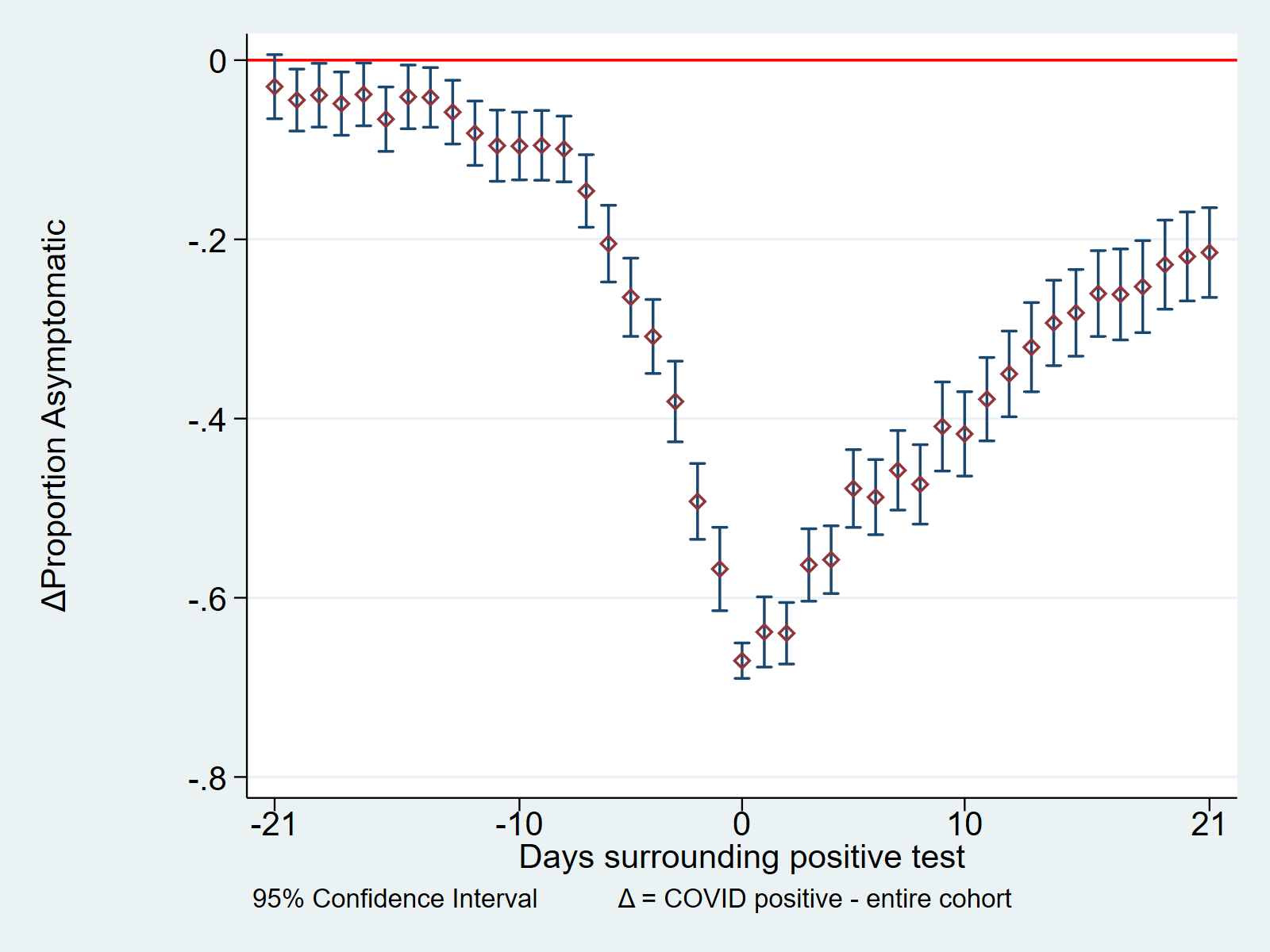

Supplement: S7 File — (ZIP) [file pone.0253566.s023.zip › sensitivity/noHCPROasymp.tif]

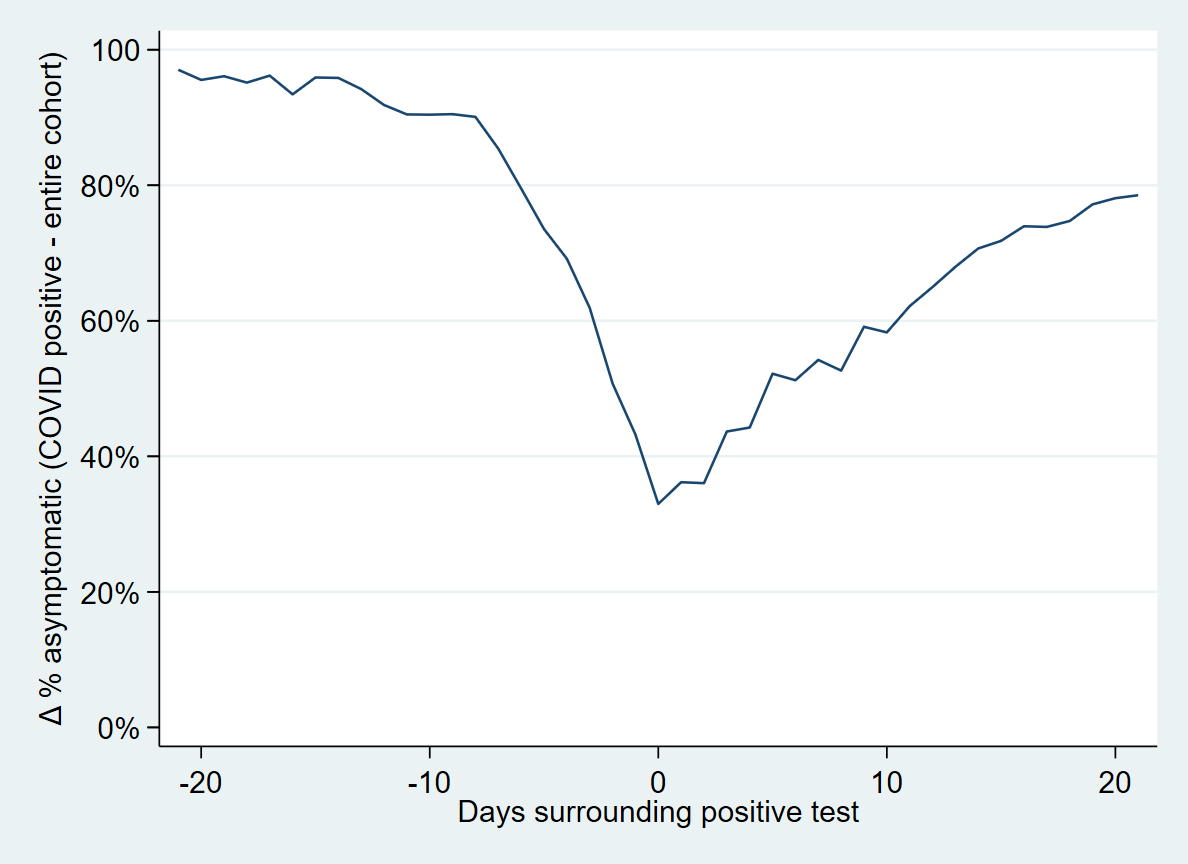

Supplement: S7 File — (ZIP) [file pone.0253566.s023.zip › sensitivity/noHCPROasymptest.tif]

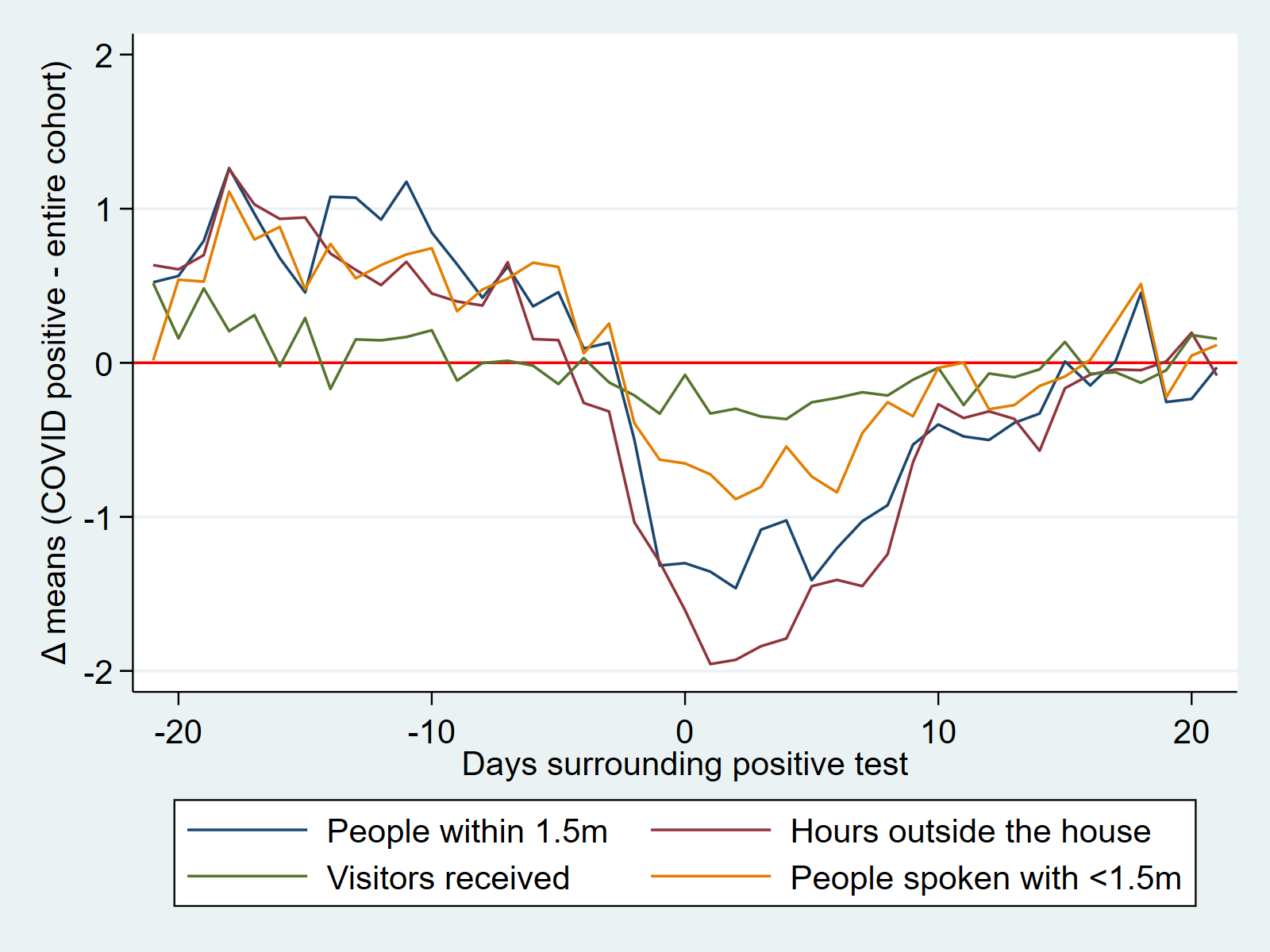

Supplement: S7 File — (ZIP) [file pone.0253566.s023.zip › sensitivity/noHCPRObehavetest.tif]

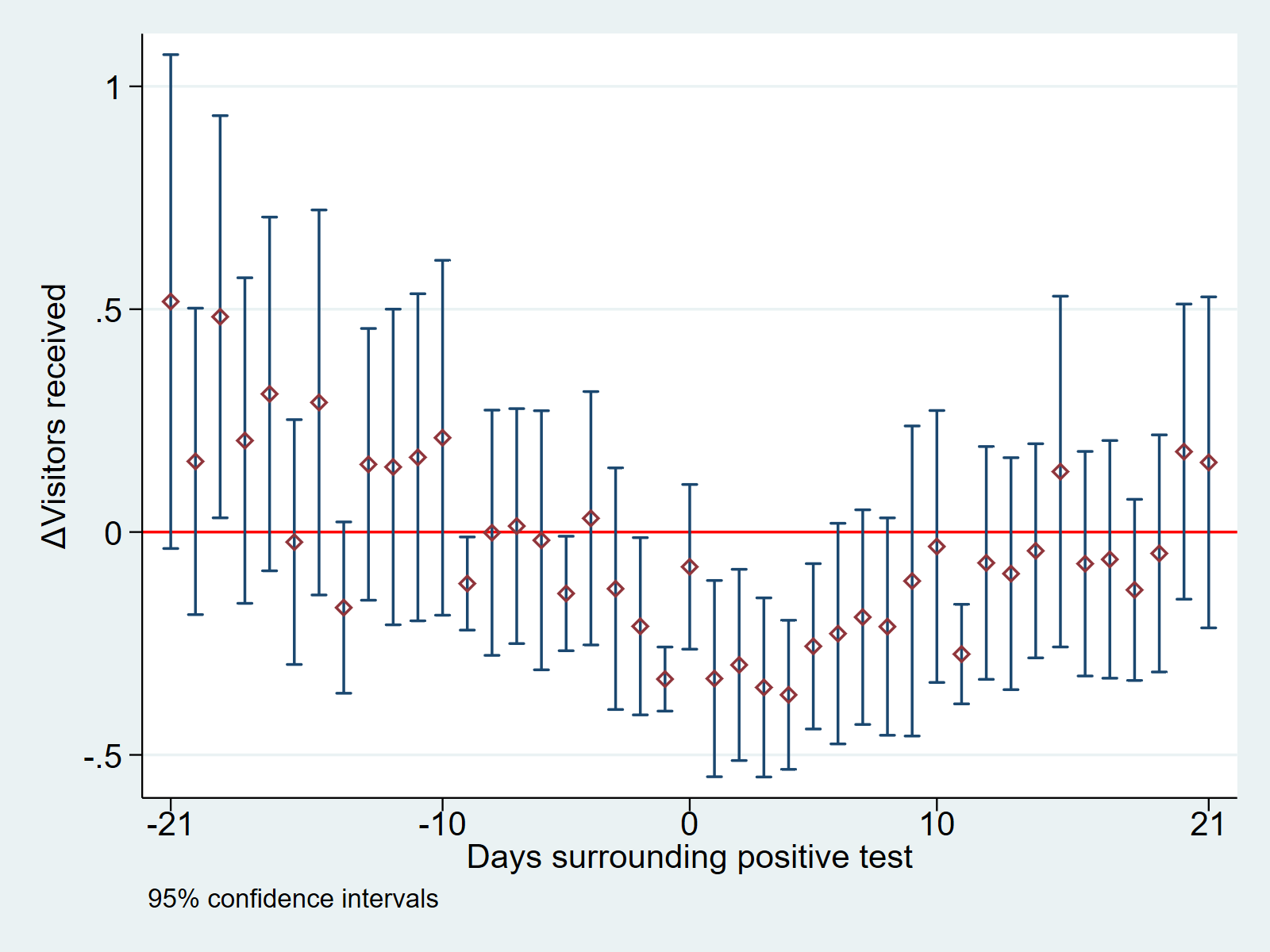

Supplement: S7 File — (ZIP) [file pone.0253566.s023.zip › sensitivity/noHCPRObezoek.tif]

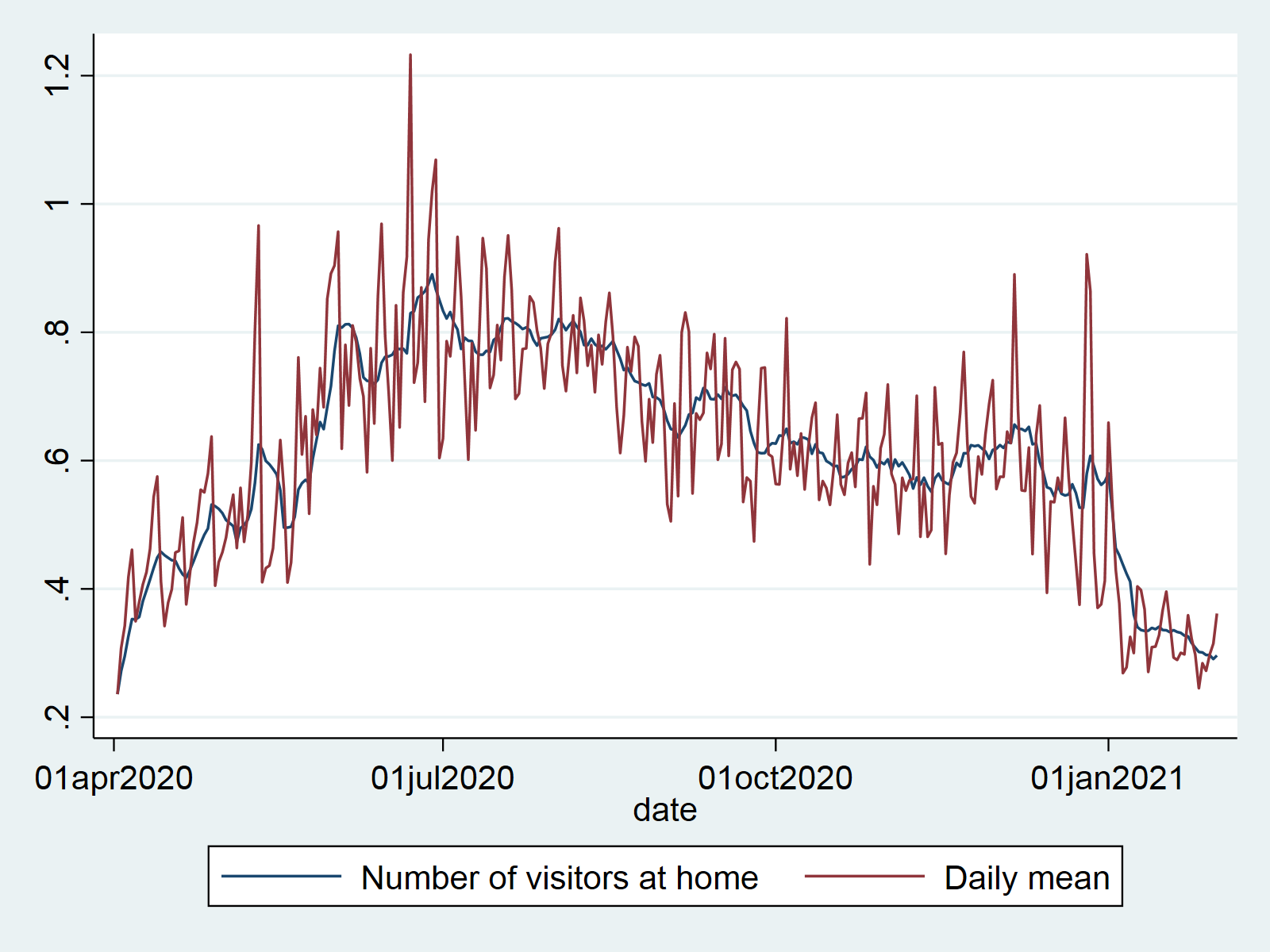

Supplement: S7 File — (ZIP) [file pone.0253566.s023.zip › sensitivity/noHCPROBezoektime.tif]

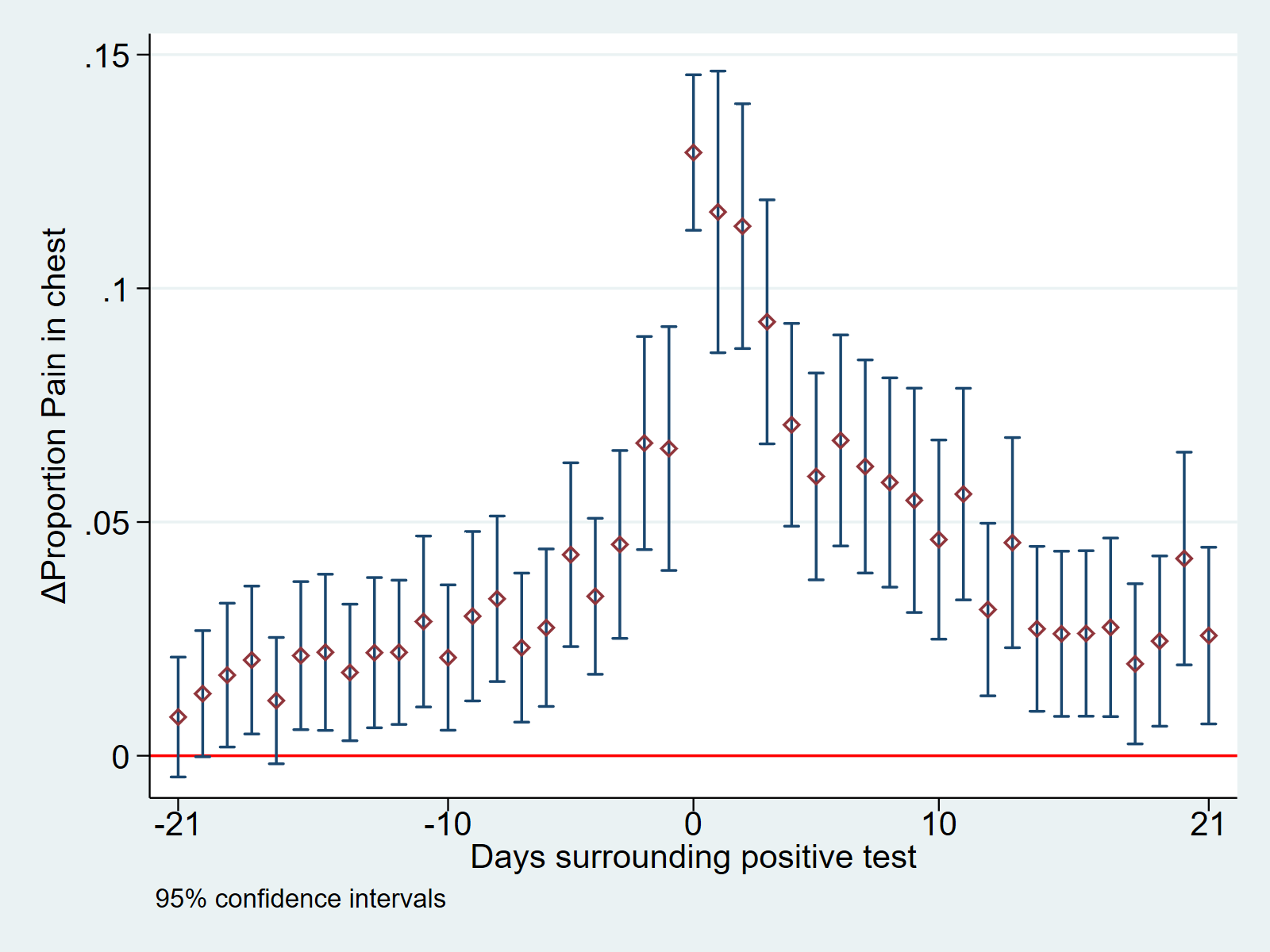

Supplement: S7 File — (ZIP) [file pone.0253566.s023.zip › sensitivity/noHCPROborstkas.tif]

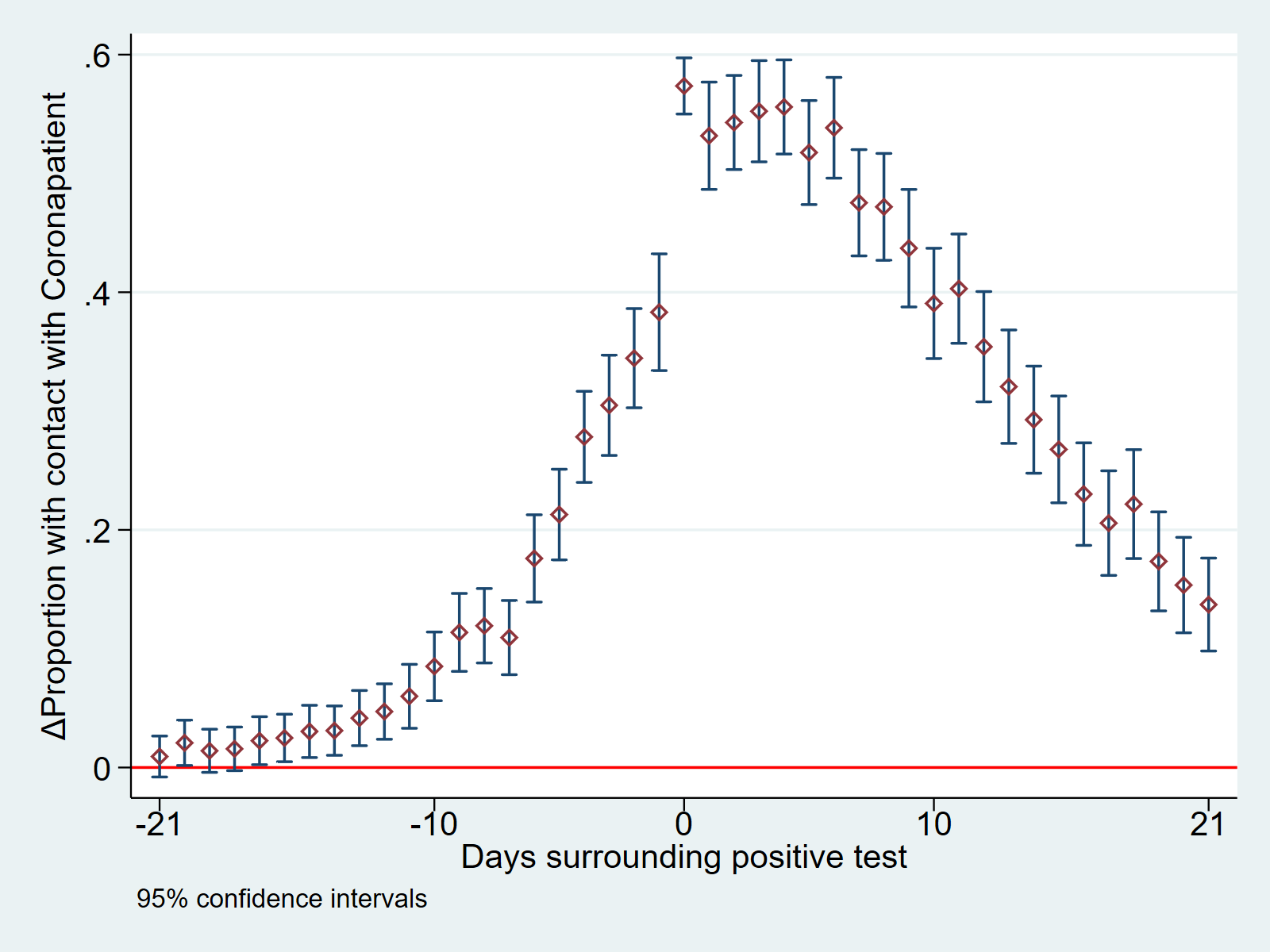

Supplement: S7 File — (ZIP) [file pone.0253566.s023.zip › sensitivity/noHCPROcontact.tif]

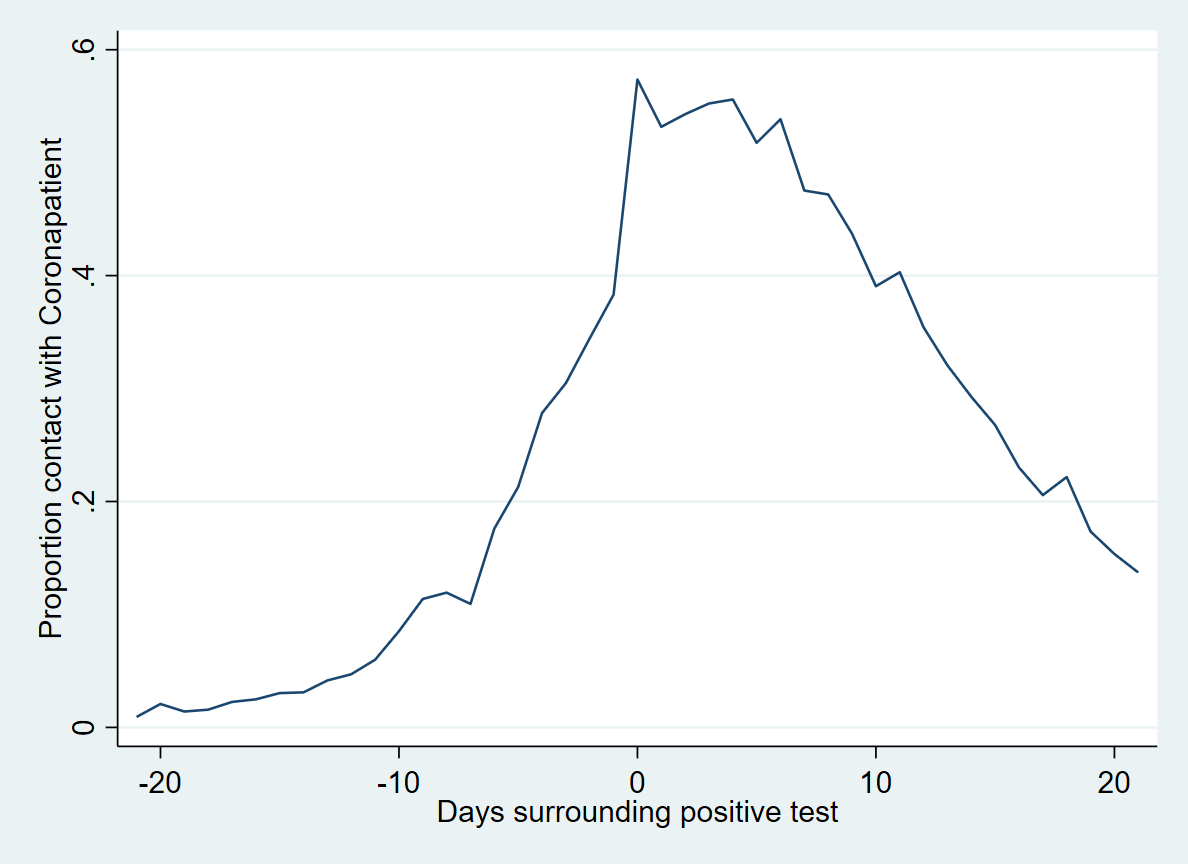

Supplement: S7 File — (ZIP) [file pone.0253566.s023.zip › sensitivity/noHCPROcontacttest.tif]

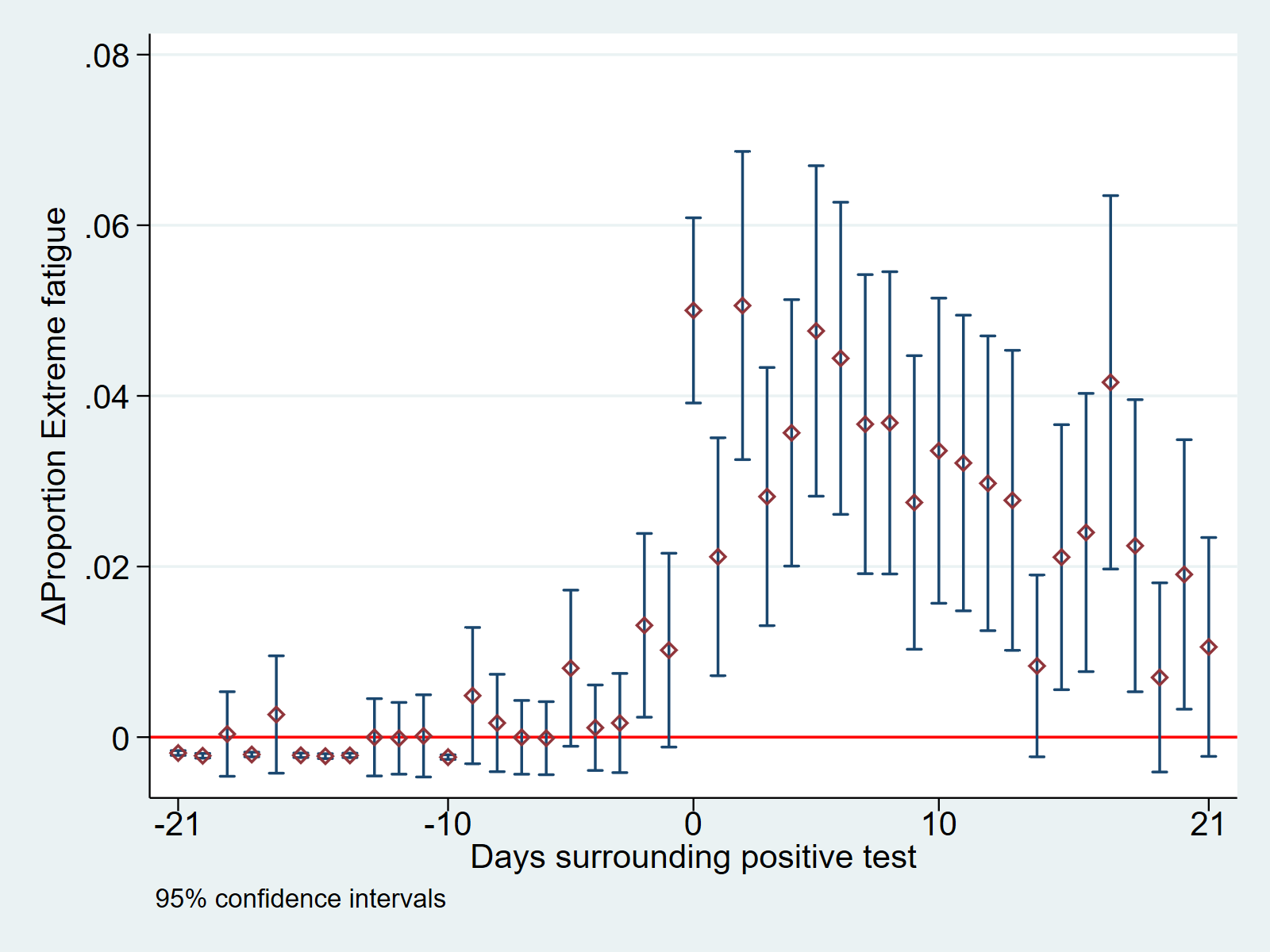

Supplement: S7 File — (ZIP) [file pone.0253566.s023.zip › sensitivity/noHCPROextreemvermoeidheid.tif]

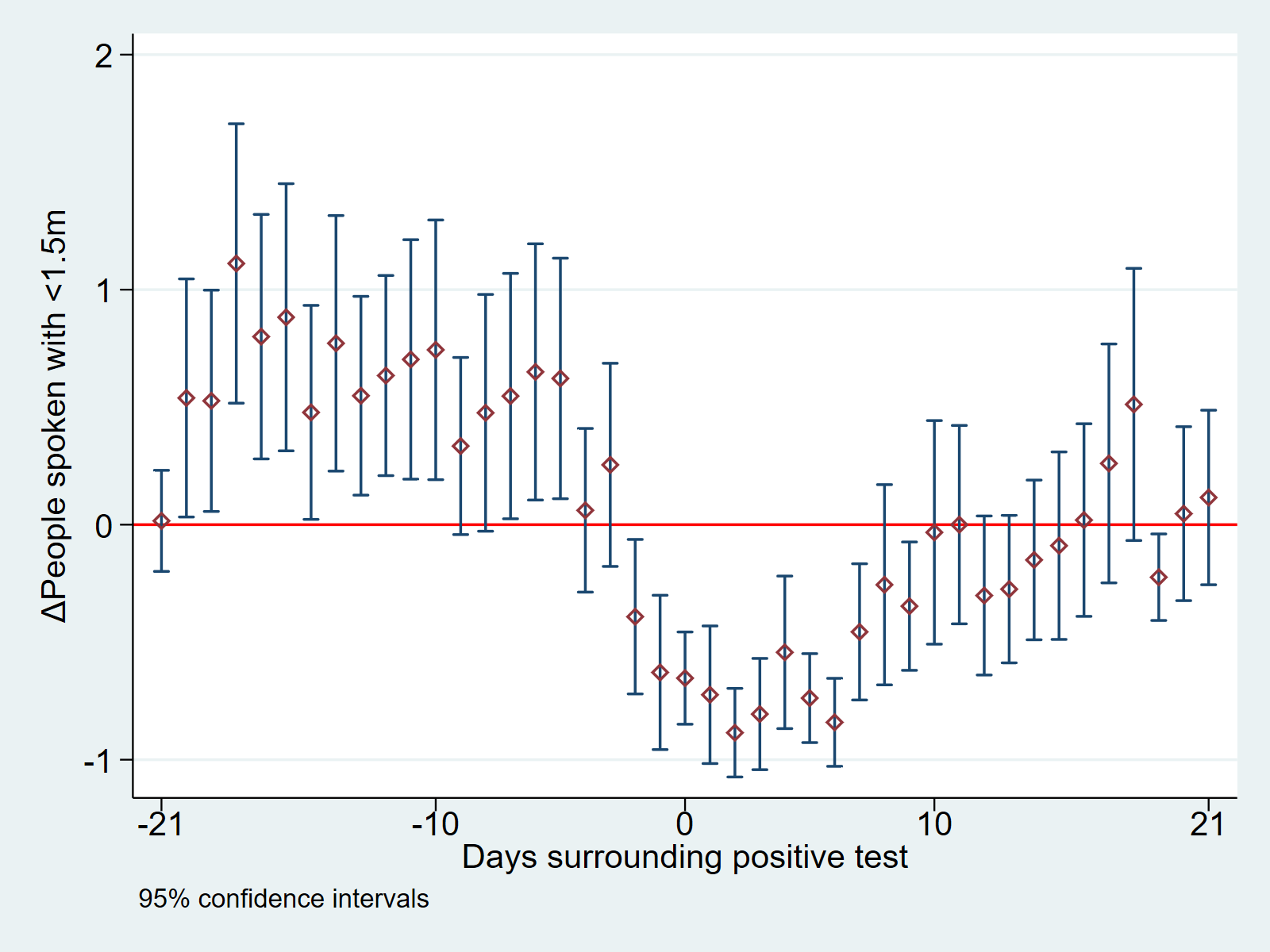

Supplement: S7 File — (ZIP) [file pone.0253566.s023.zip › sensitivity/noHCPROgesproken.tif]

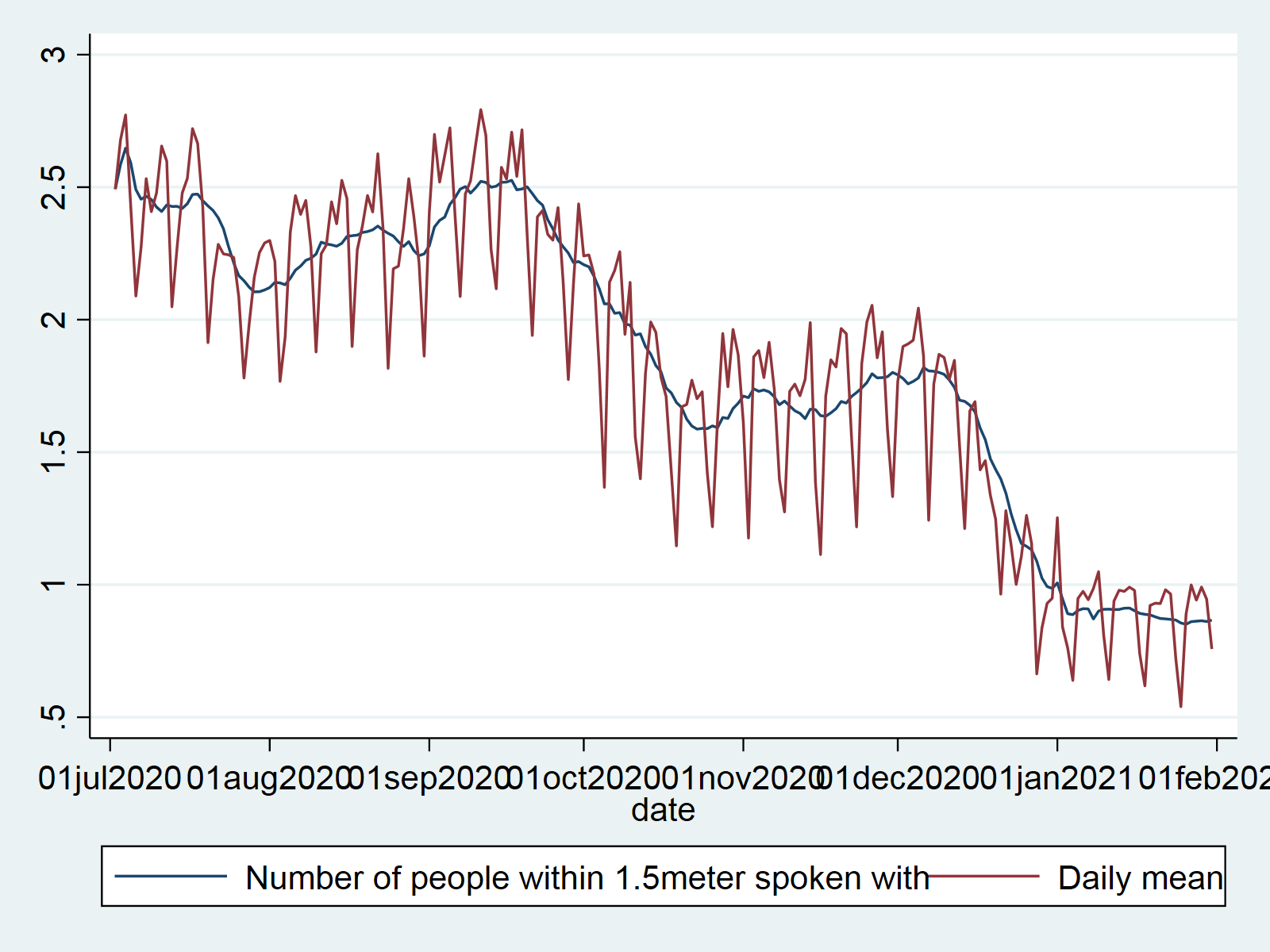

Supplement: S7 File — (ZIP) [file pone.0253566.s023.zip › sensitivity/noHCPROGesprokentime.tif]

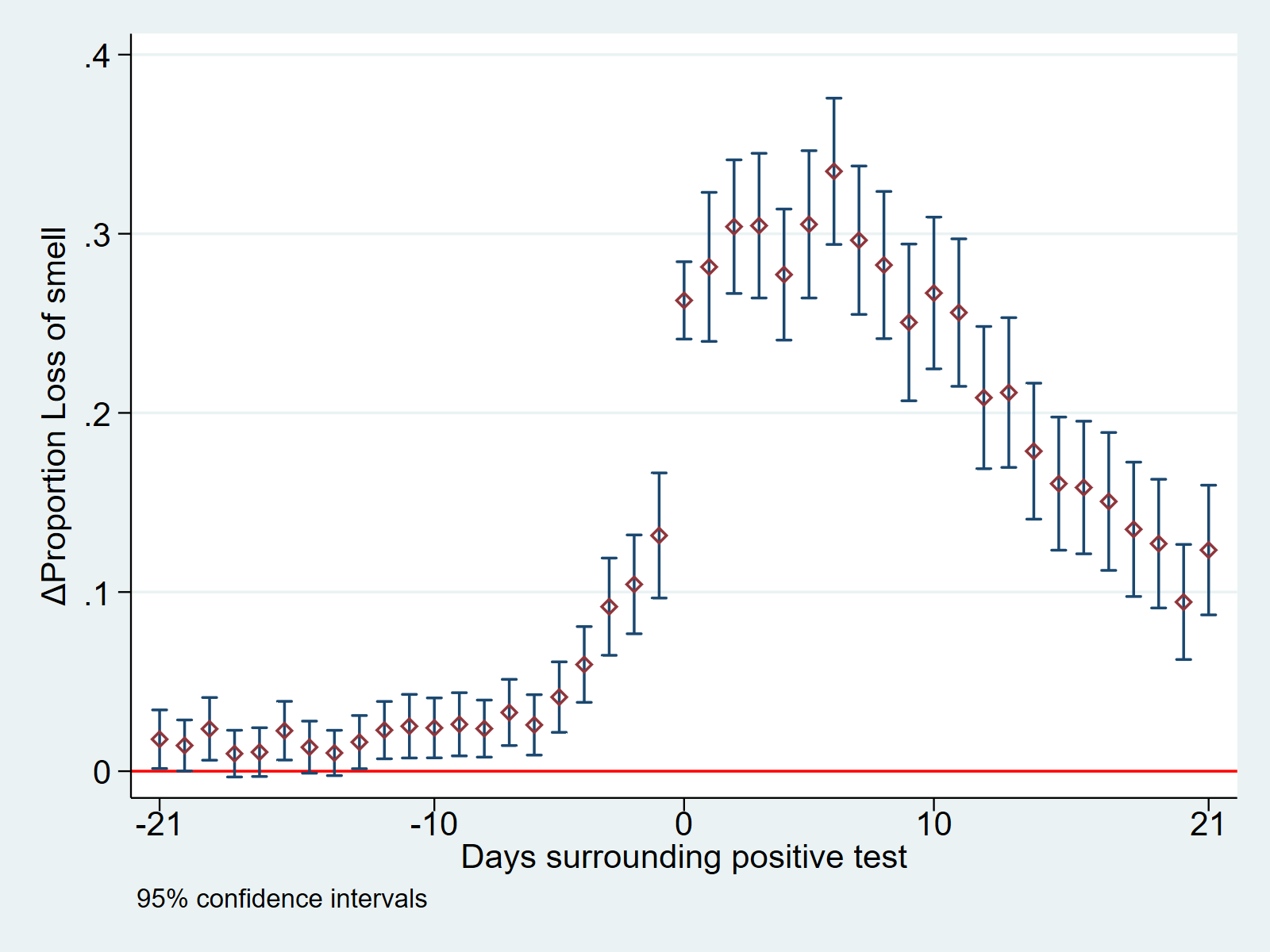

Supplement: S7 File — (ZIP) [file pone.0253566.s023.zip › sensitivity/noHCPROgeur.tif]

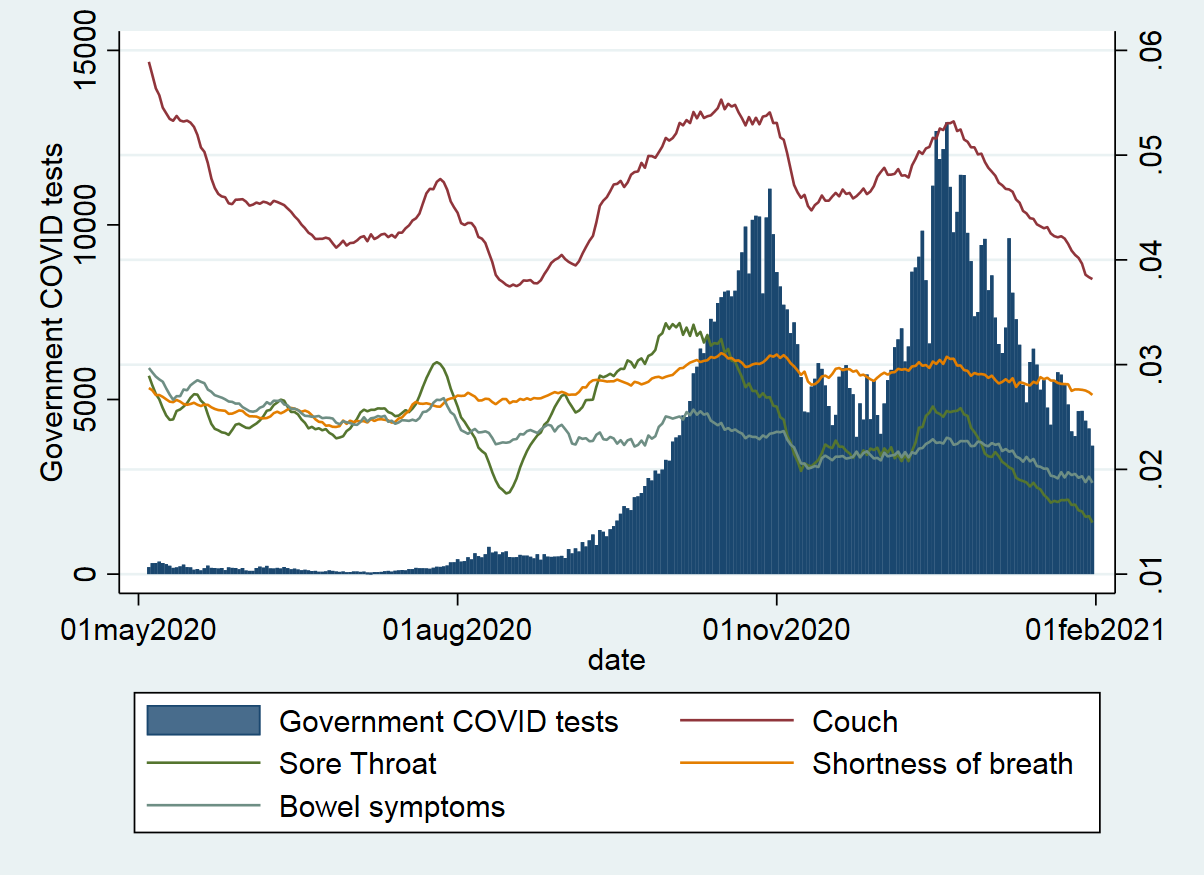

Supplement: S7 File — (ZIP) [file pone.0253566.s023.zip › sensitivity/noHCPROhighsypmtime.tif]

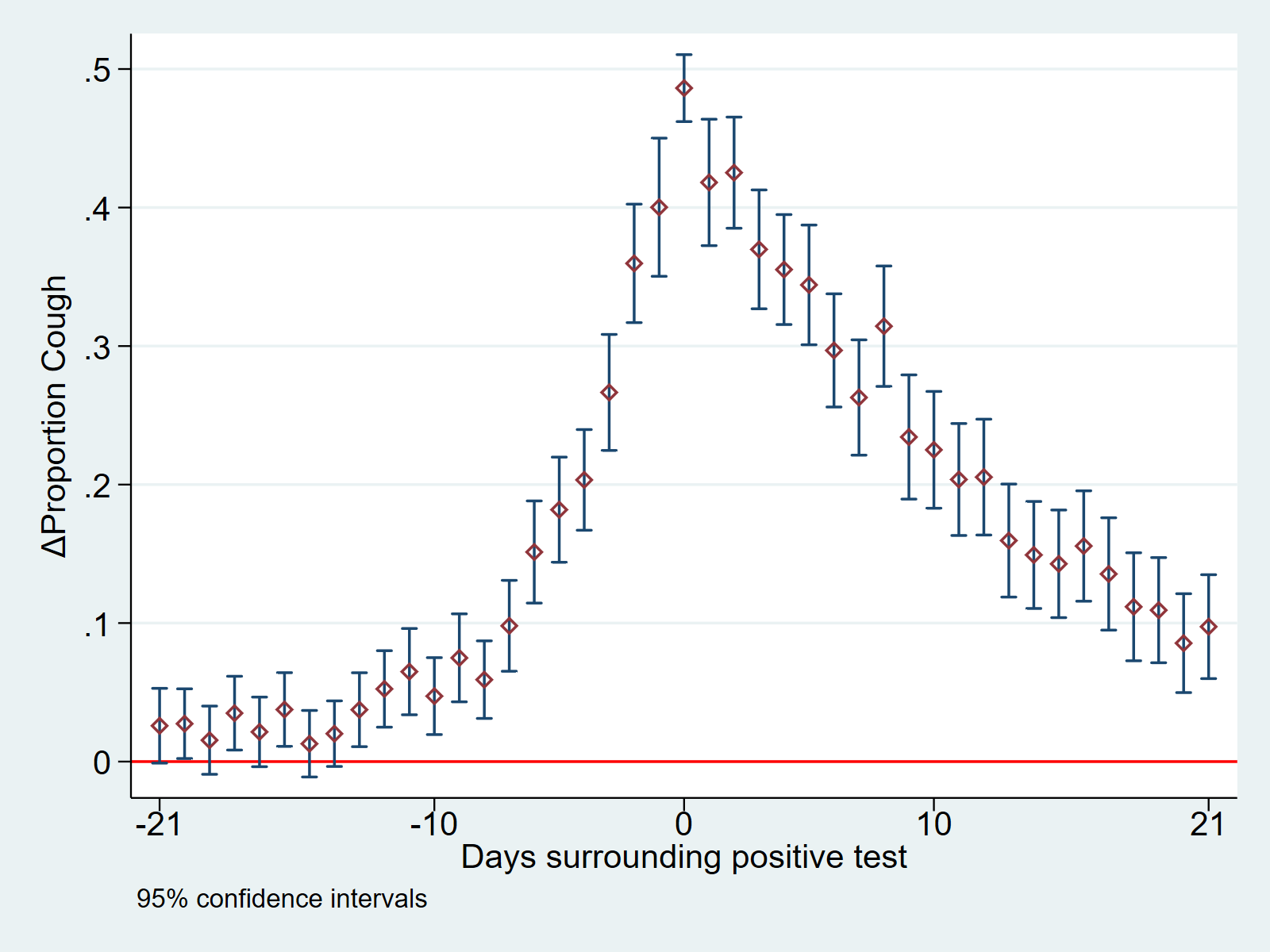

Supplement: S7 File — (ZIP) [file pone.0253566.s023.zip › sensitivity/noHCPROhoesten.tif]

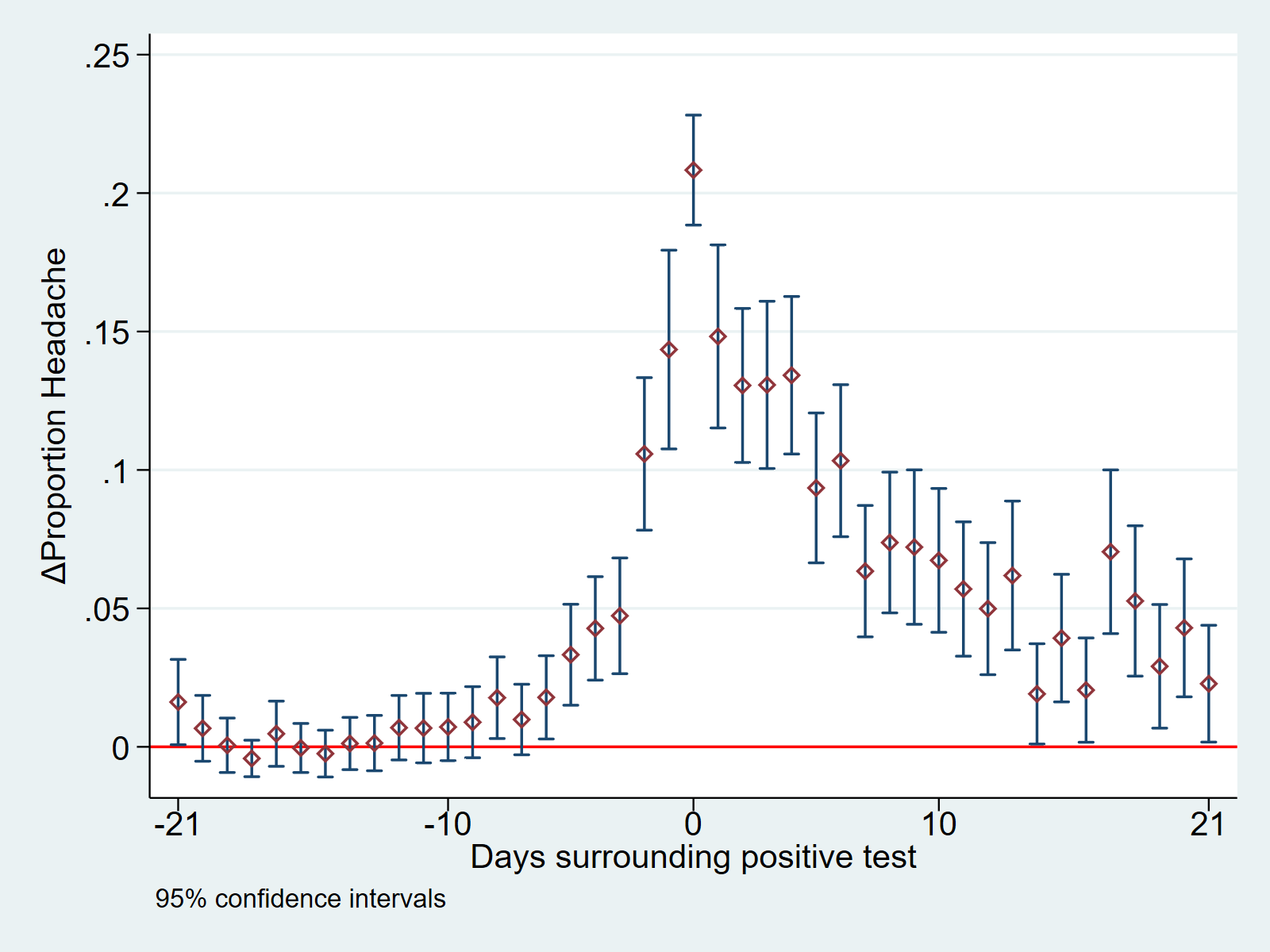

Supplement: S7 File — (ZIP) [file pone.0253566.s023.zip › sensitivity/noHCPROhoofdpijn.tif]

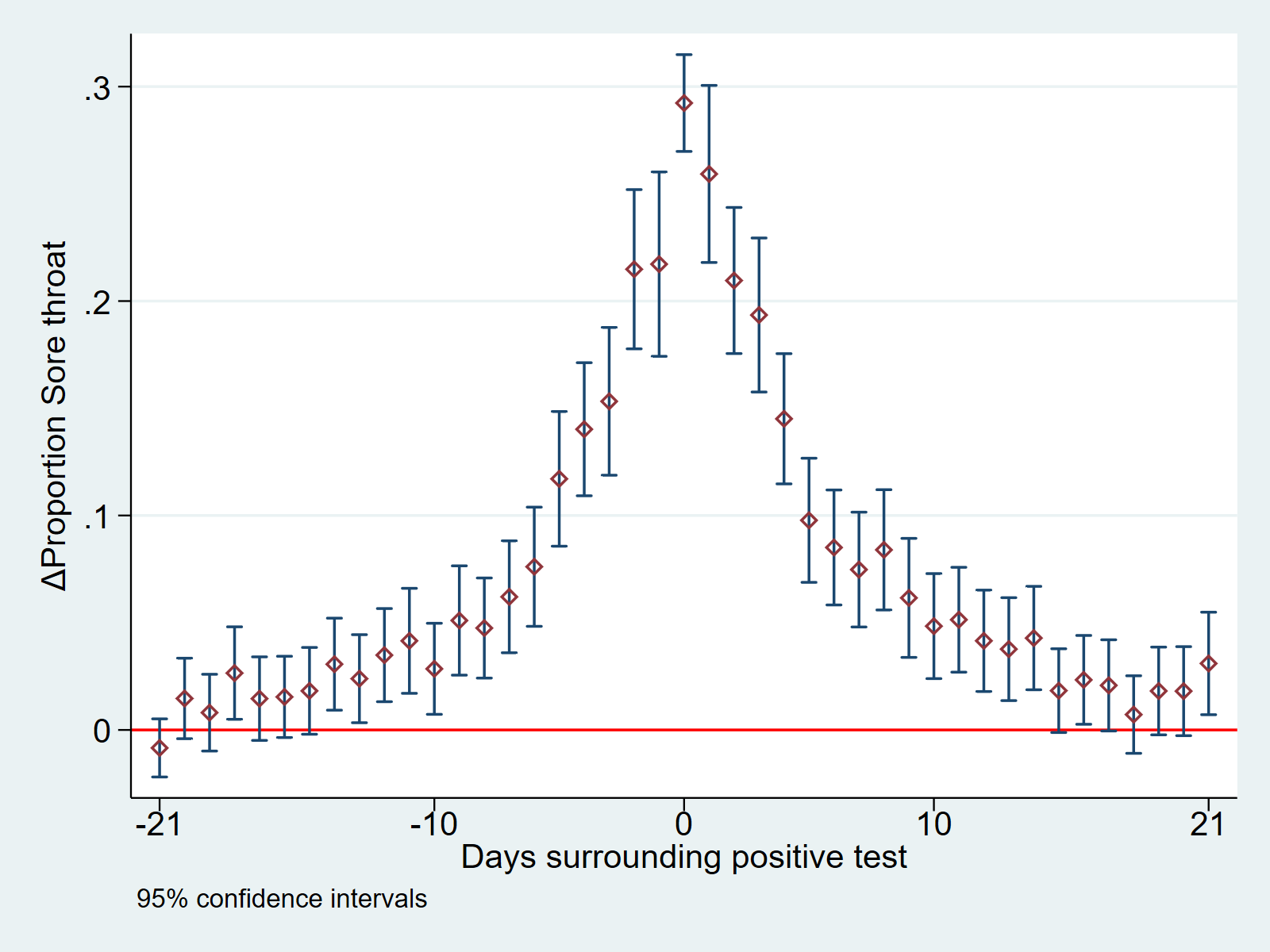

Supplement: S7 File — (ZIP) [file pone.0253566.s023.zip › sensitivity/noHCPROkeelpijn.tif]

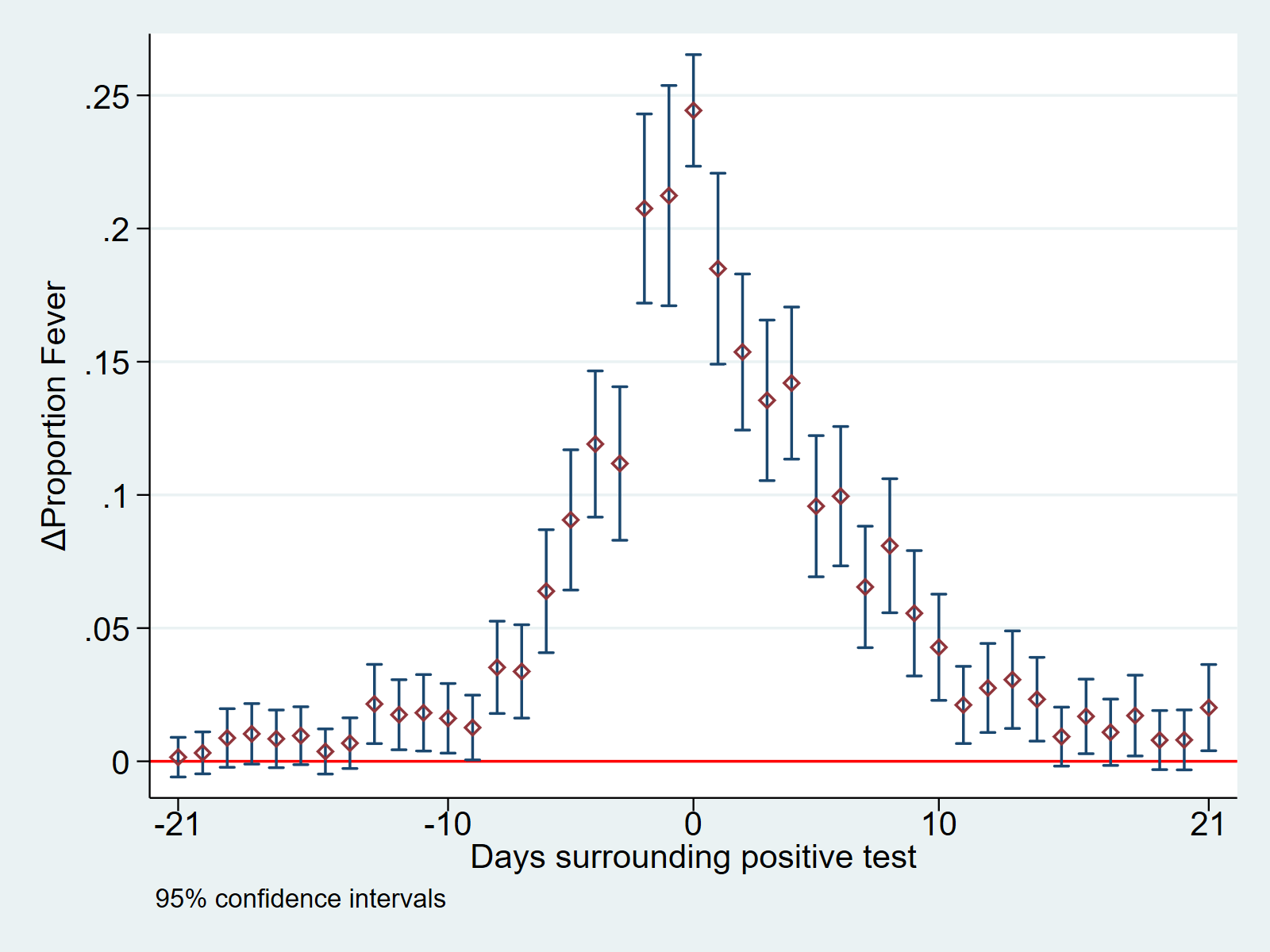

Supplement: S7 File — (ZIP) [file pone.0253566.s023.zip › sensitivity/noHCPROkoorts.tif]

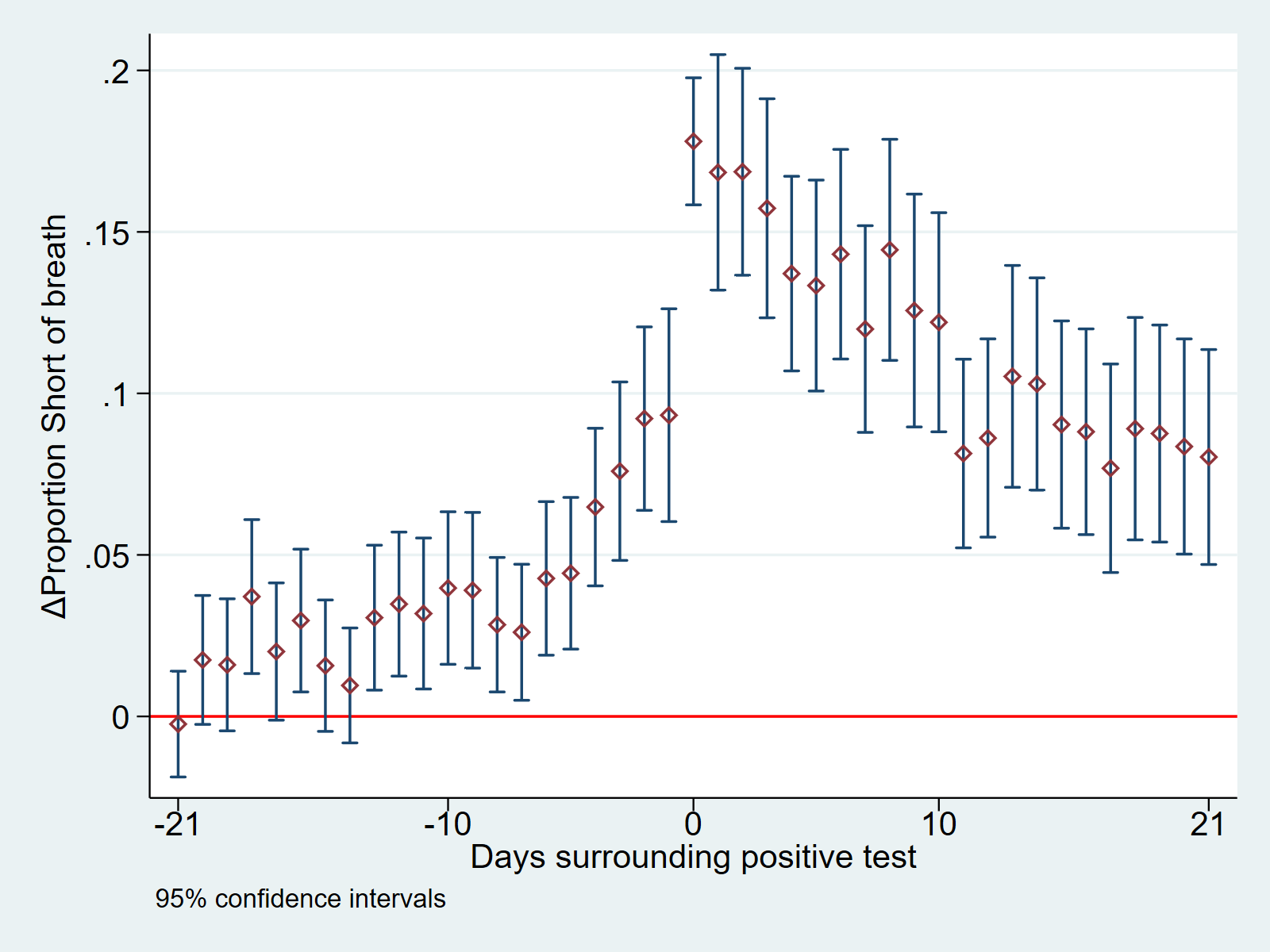

Supplement: S7 File — (ZIP) [file pone.0253566.s023.zip › sensitivity/noHCPROkortademig.tif]

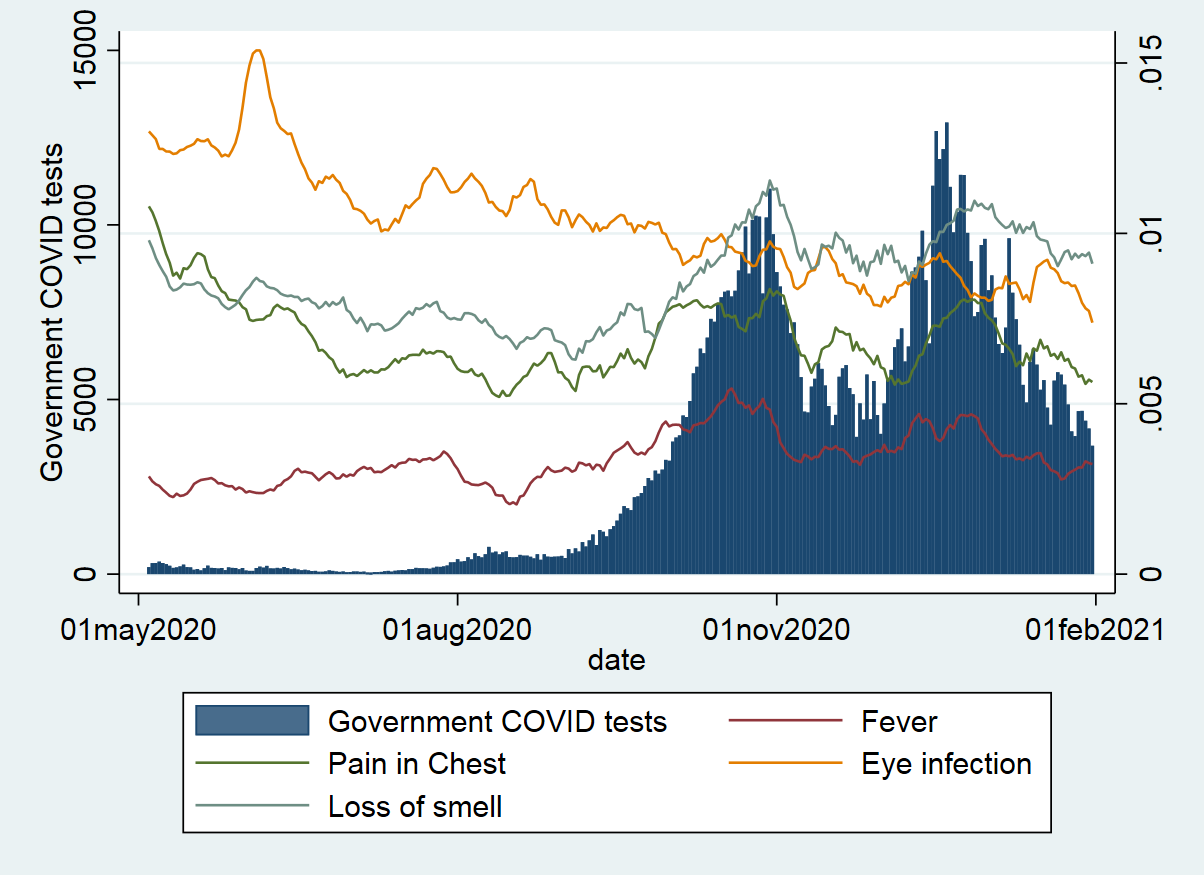

Supplement: S7 File — (ZIP) [file pone.0253566.s023.zip › sensitivity/noHCPROlowsypmtime.tif]

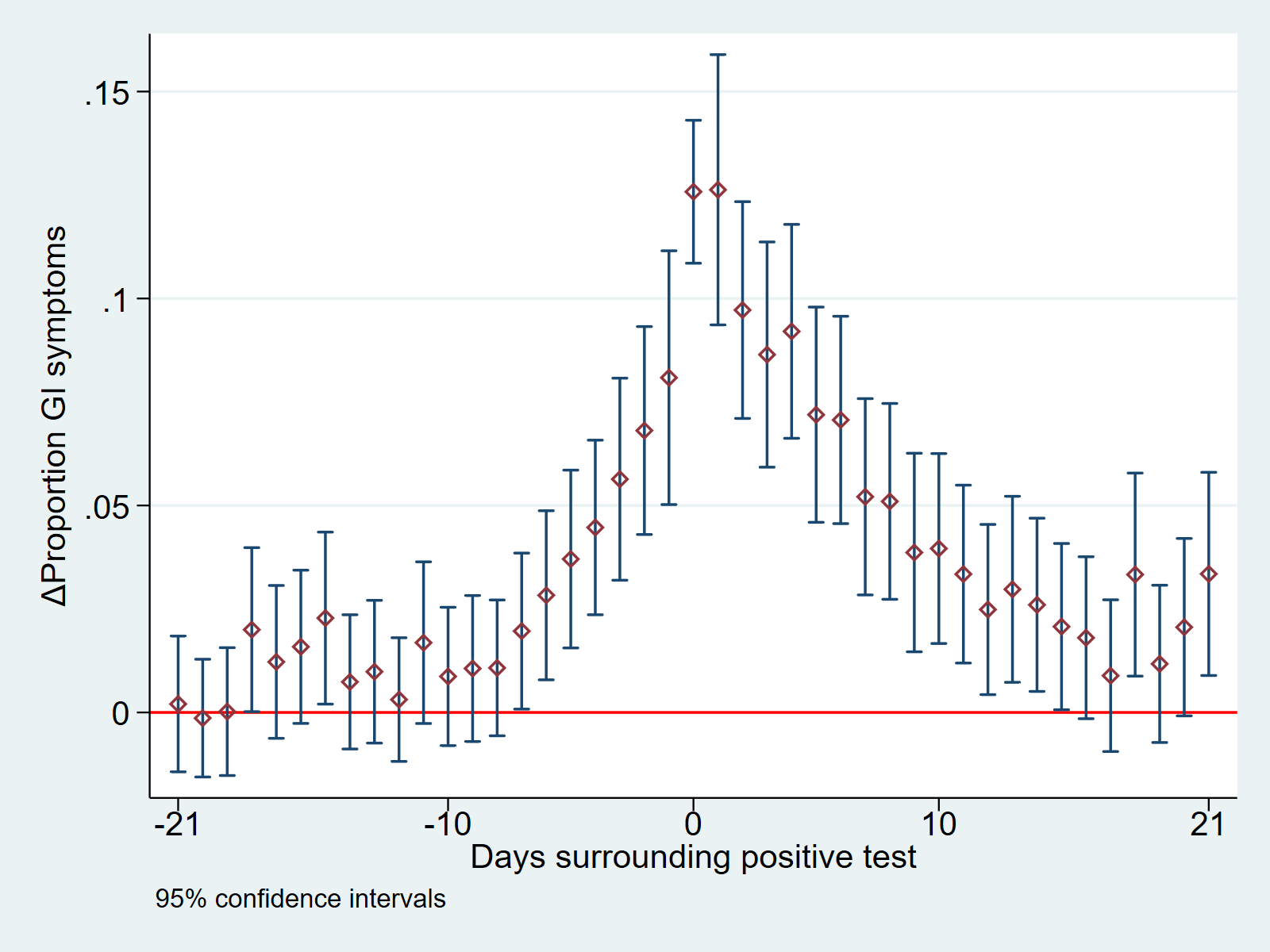

Supplement: S7 File — (ZIP) [file pone.0253566.s023.zip › sensitivity/noHCPROmaagdarm.tif]

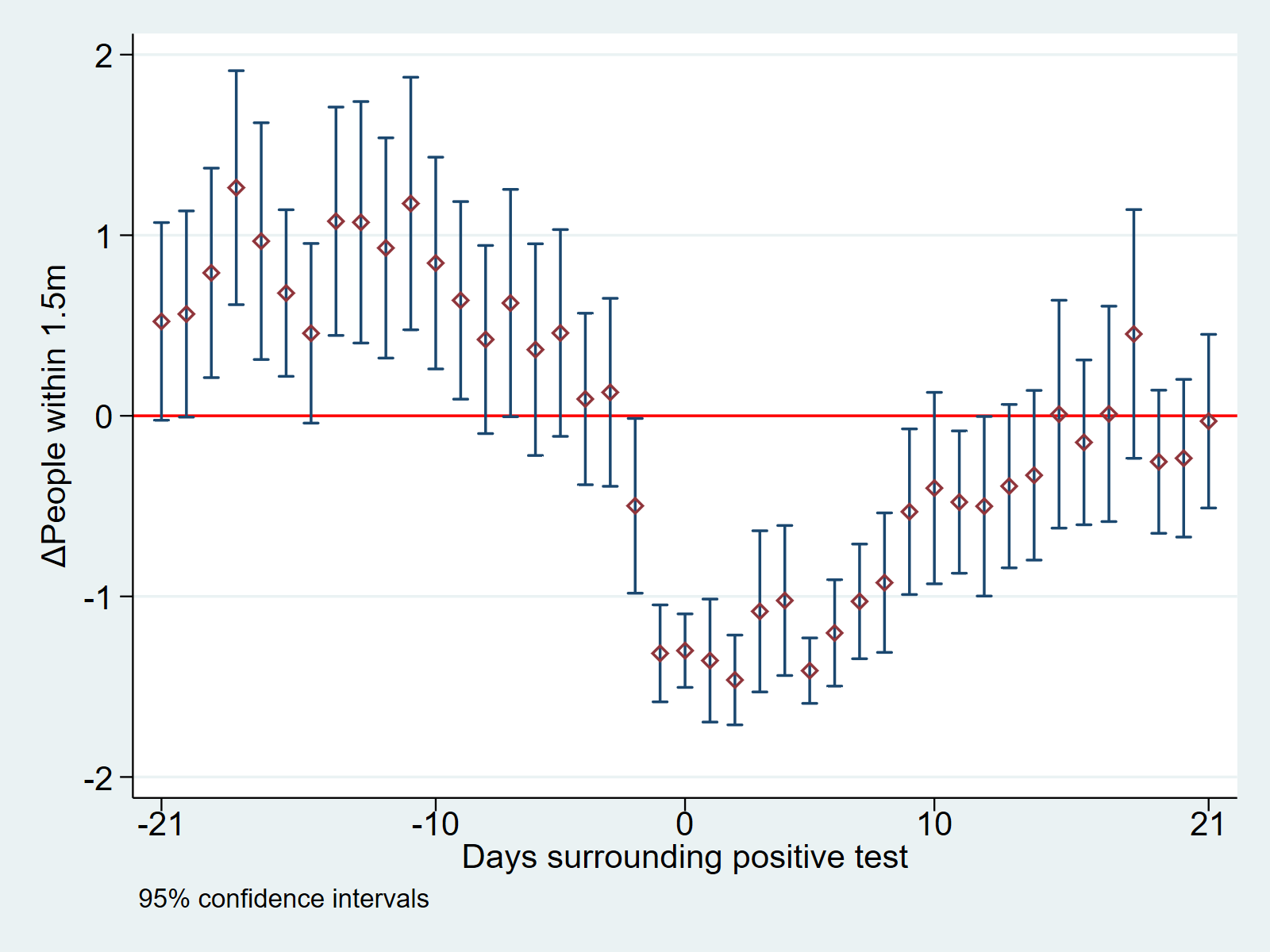

Supplement: S7 File — (ZIP) [file pone.0253566.s023.zip › sensitivity/noHCPROnabij.tif]

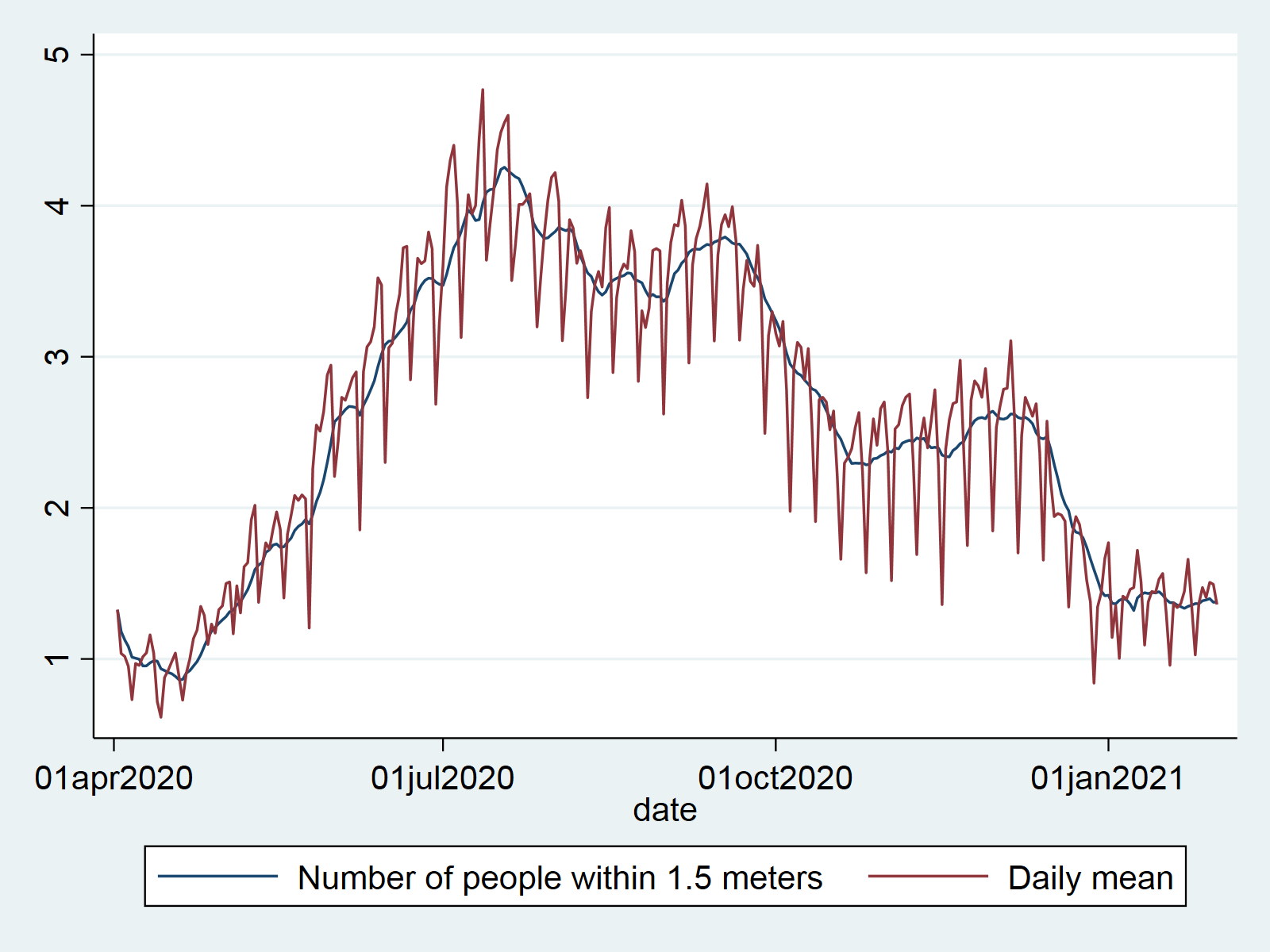

Supplement: S7 File — (ZIP) [file pone.0253566.s023.zip › sensitivity/noHCPRONabijtime.tif]

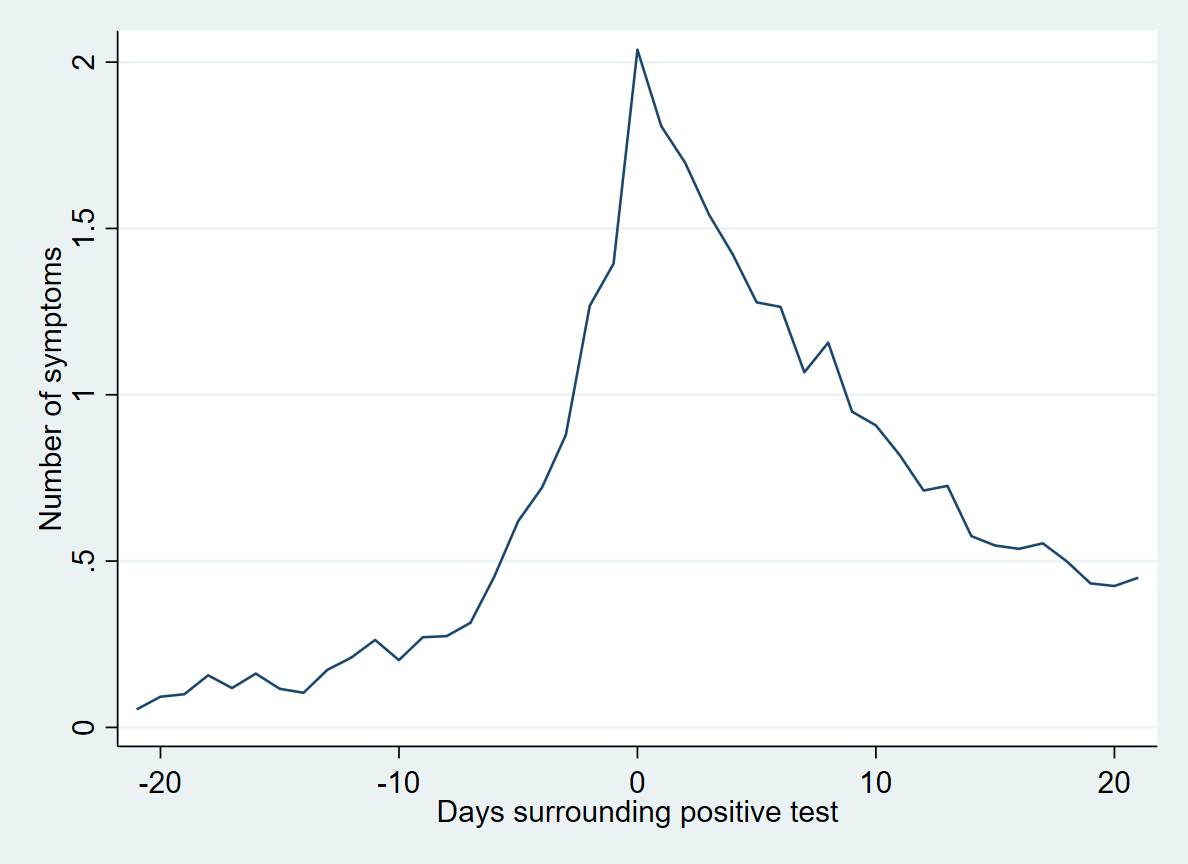

Supplement: S7 File — (ZIP) [file pone.0253566.s023.zip › sensitivity/noHCPROnsymptest.tif]

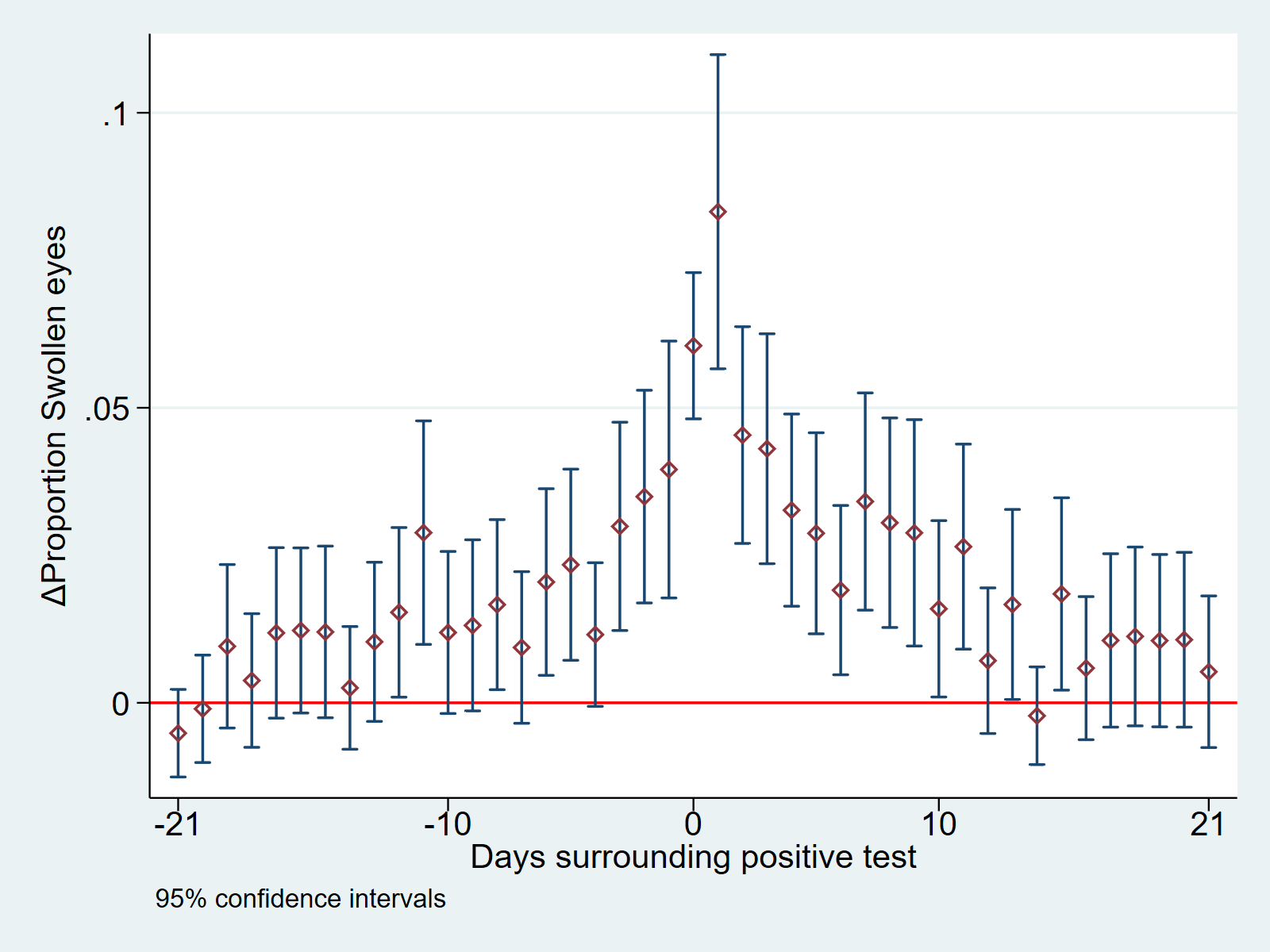

Supplement: S7 File — (ZIP) [file pone.0253566.s023.zip › sensitivity/noHCPROogen.tif]

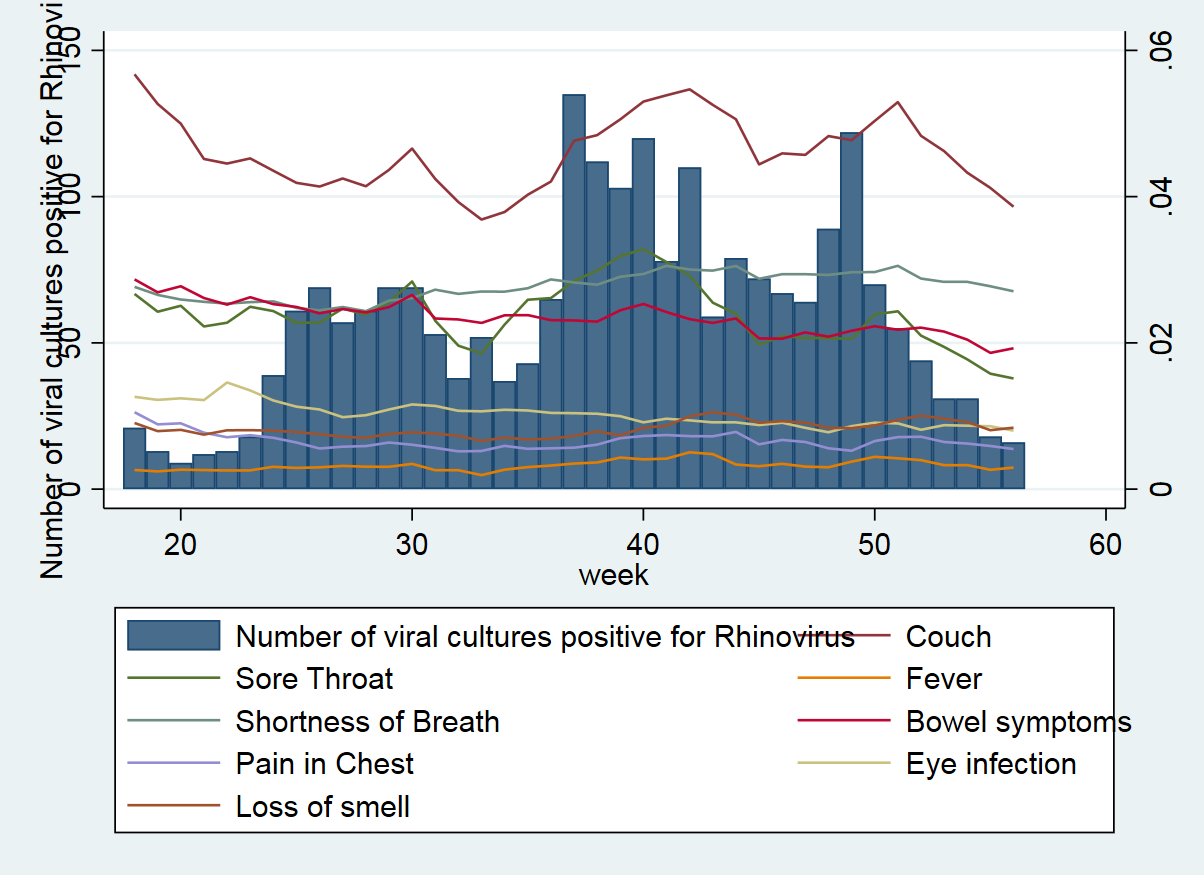

Supplement: S7 File — (ZIP) [file pone.0253566.s023.zip › sensitivity/noHCPROrhinovirus.tif]

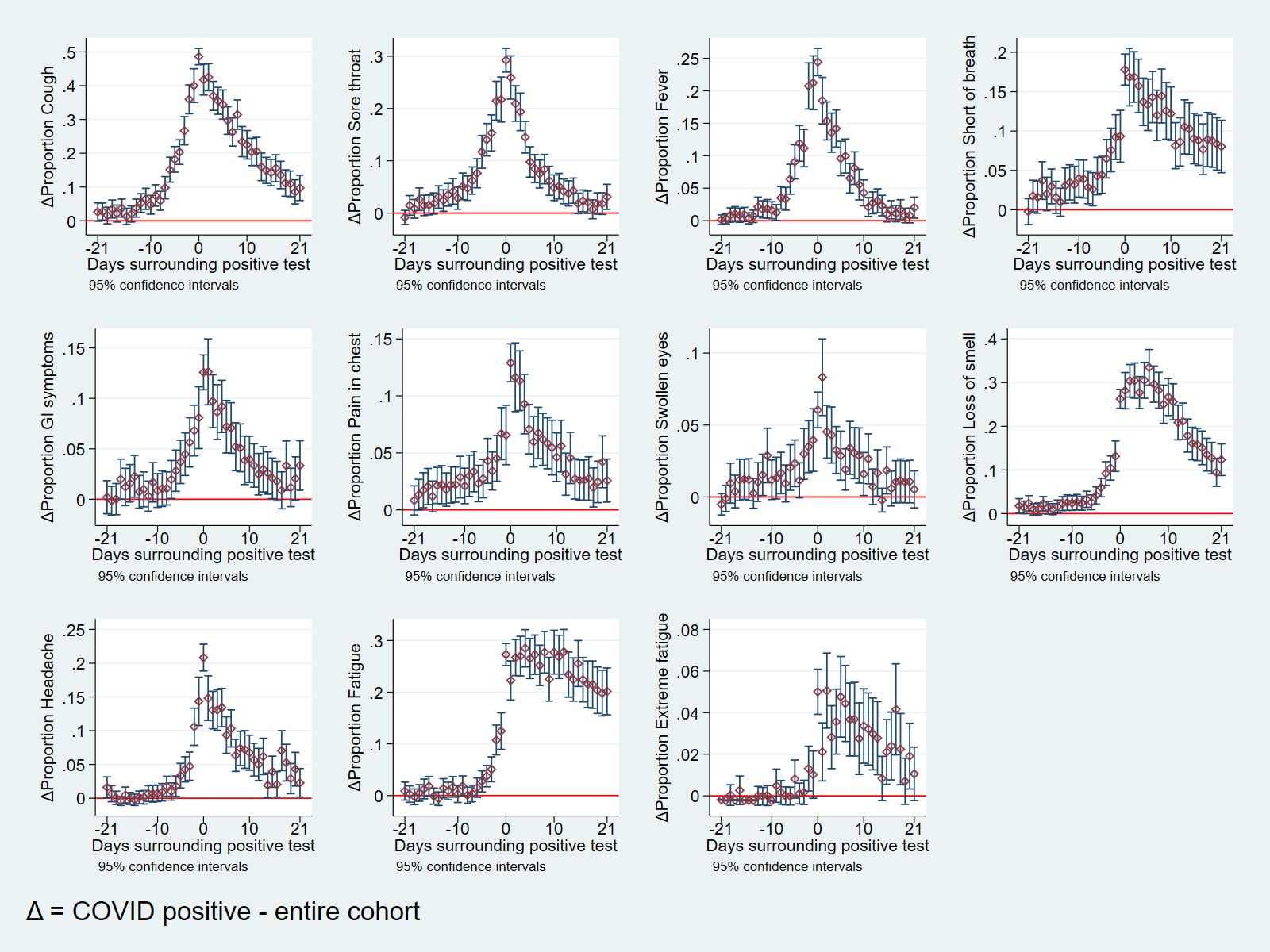

Supplement: S7 File — (ZIP) [file pone.0253566.s023.zip › sensitivity/noHCPROS6-S16AllSymp.tif]

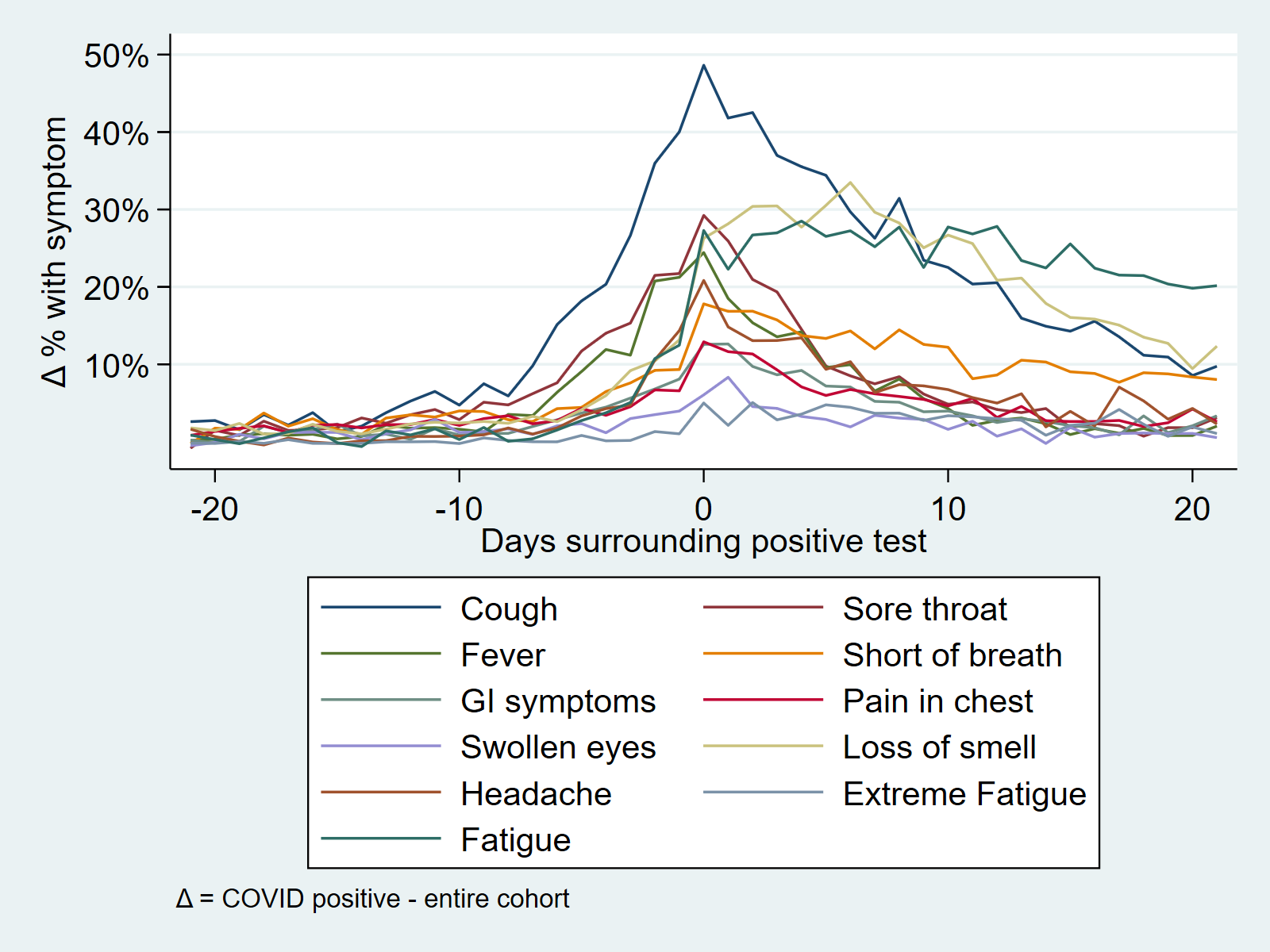

Supplement: S7 File — (ZIP) [file pone.0253566.s023.zip › sensitivity/noHCPROsymptomstest.tif]

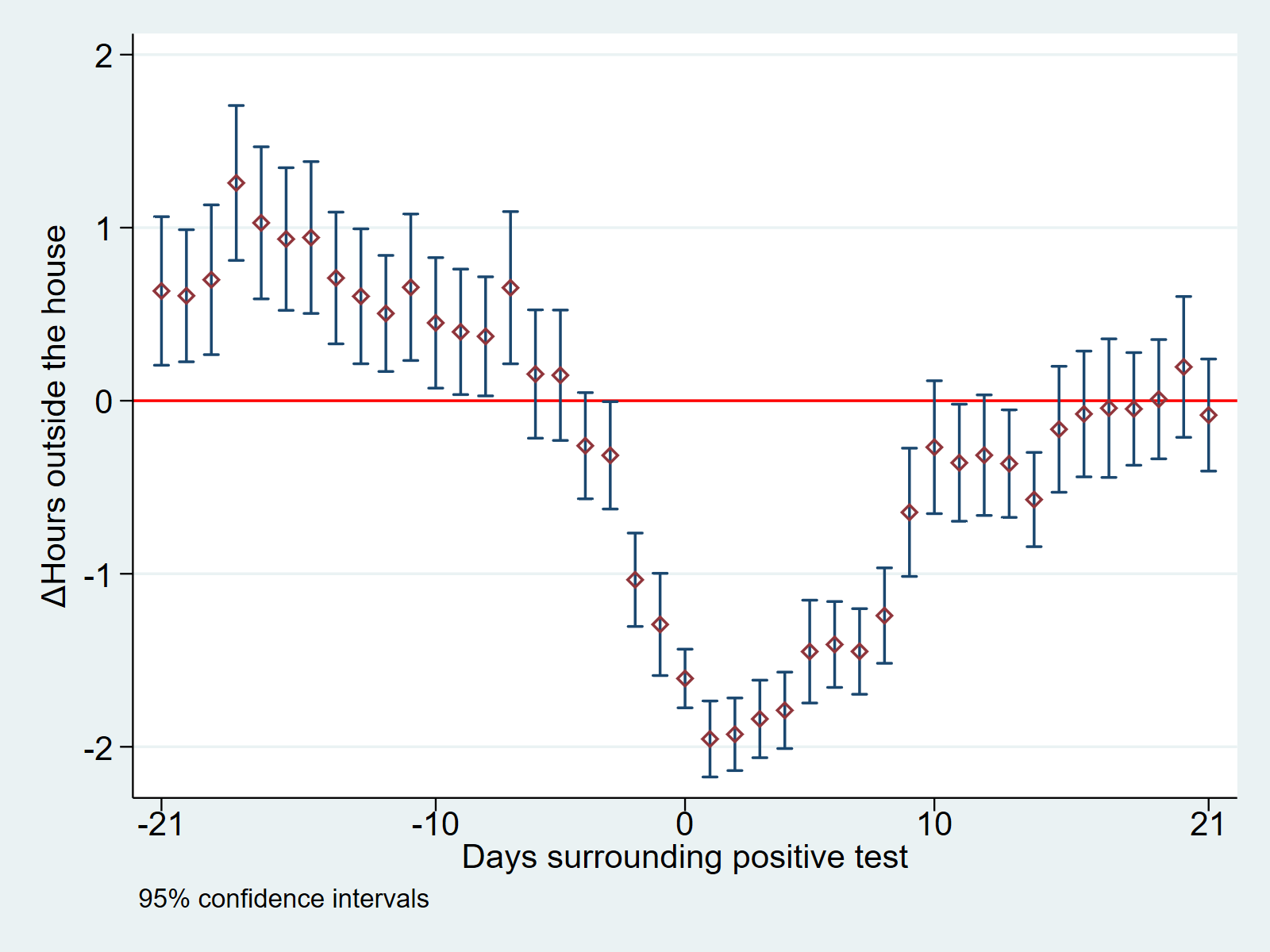

Supplement: S7 File — (ZIP) [file pone.0253566.s023.zip › sensitivity/noHCPROuithuis.tif]

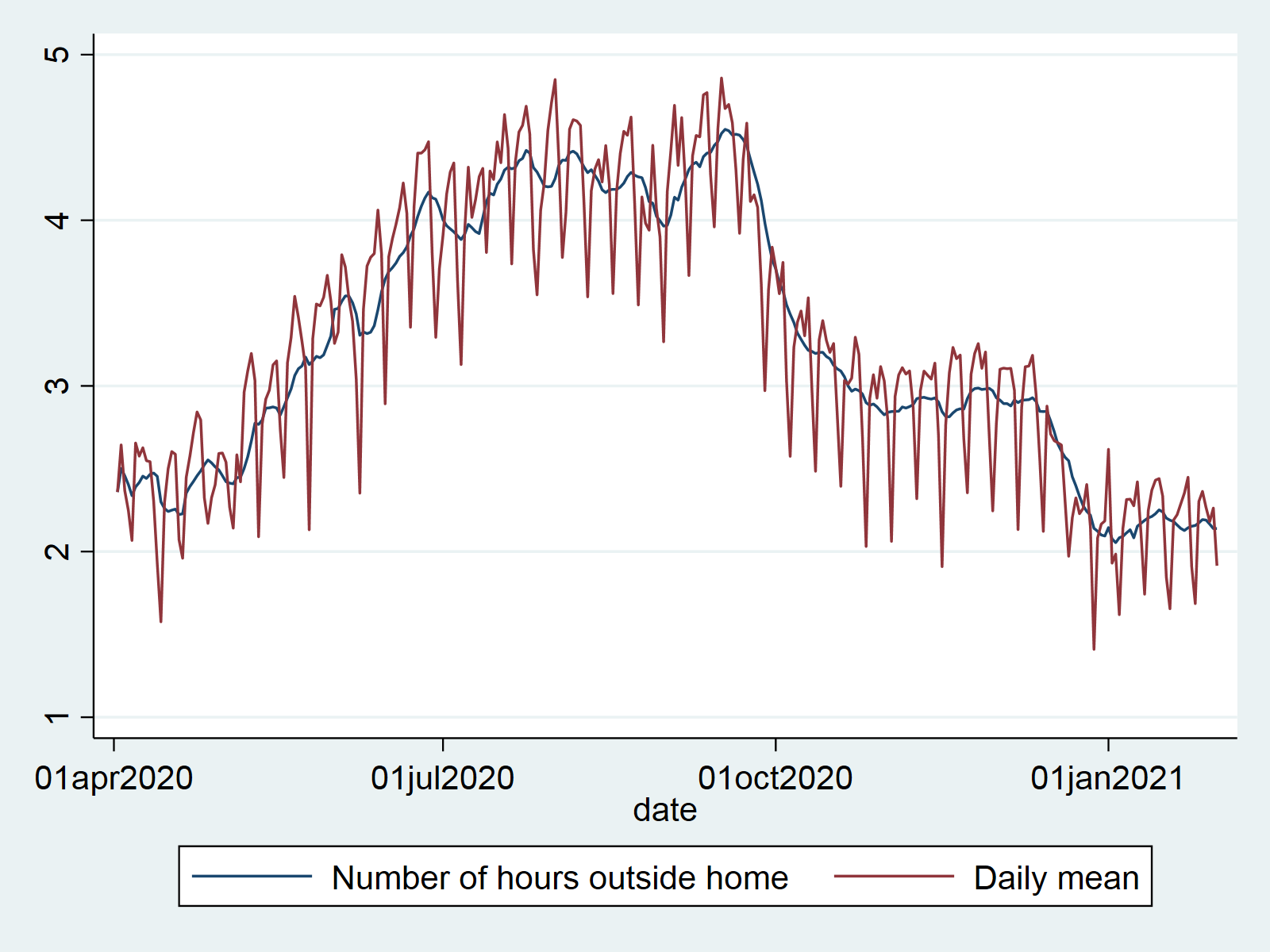

Supplement: S7 File — (ZIP) [file pone.0253566.s023.zip › sensitivity/noHCPROUithuistime.tif]

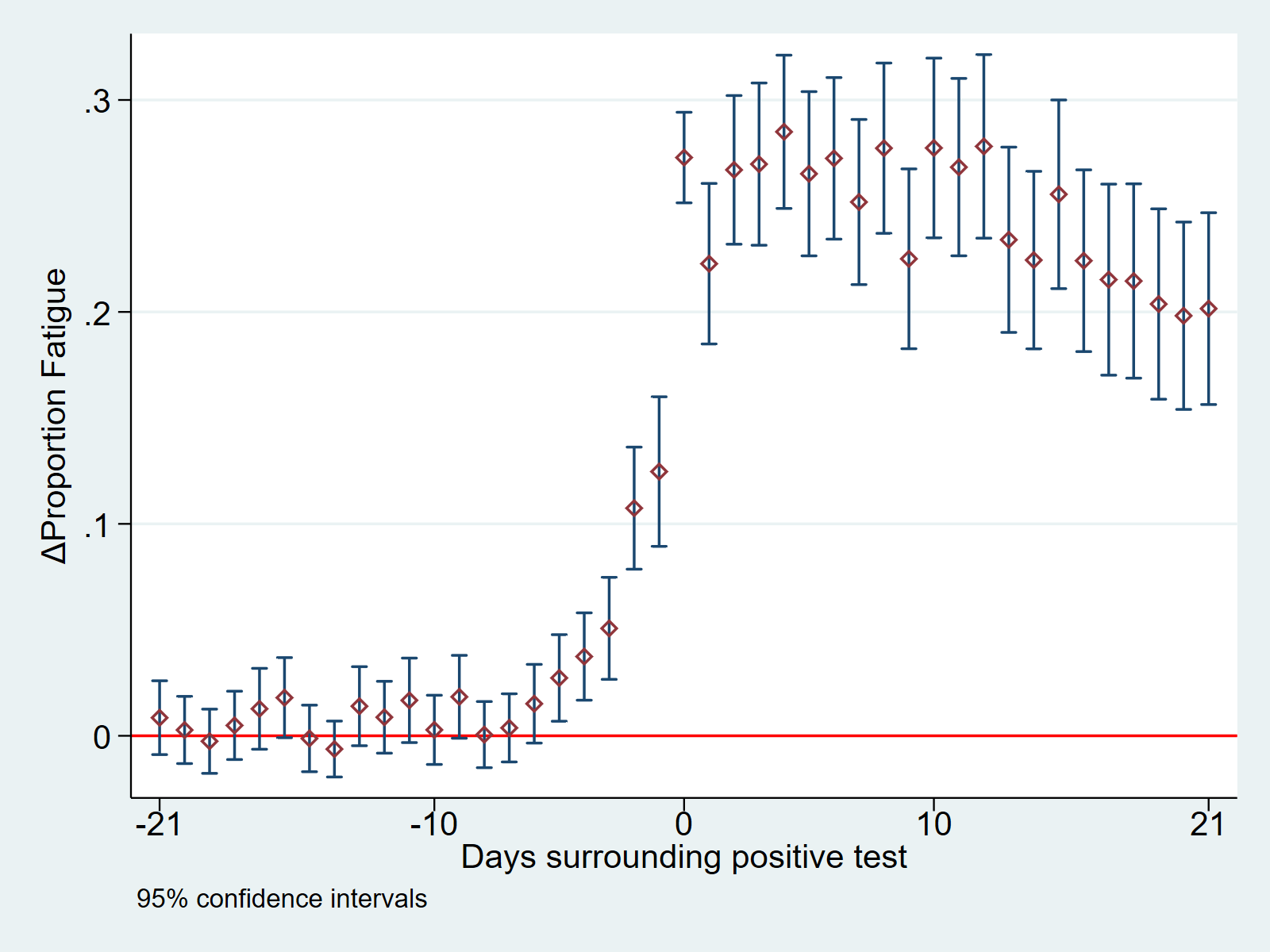

Supplement: S7 File — (ZIP) [file pone.0253566.s023.zip › sensitivity/noHCPROvermoeidheid.tif]

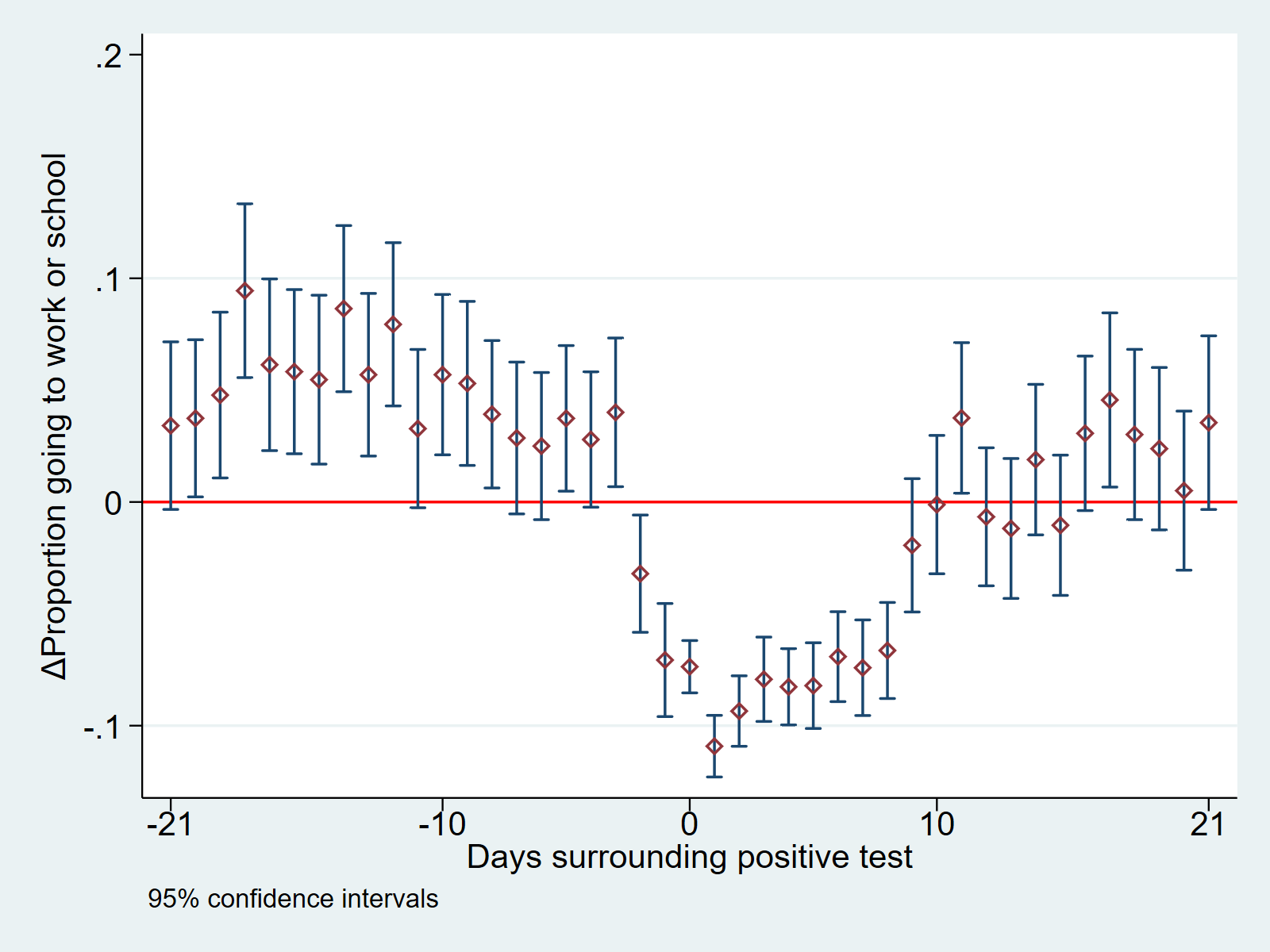

Supplement: S7 File — (ZIP) [file pone.0253566.s023.zip › sensitivity/noHCPROwerk.tif]

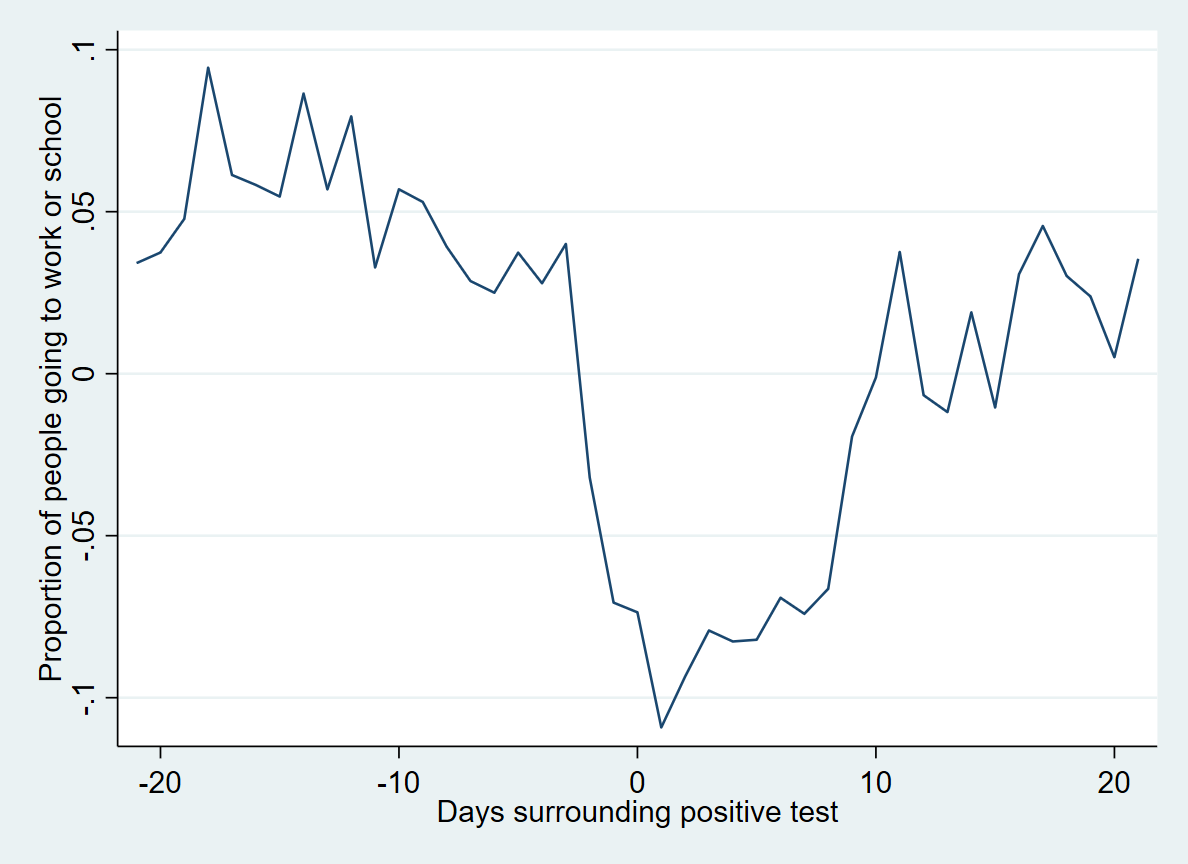

Supplement: S7 File — (ZIP) [file pone.0253566.s023.zip › sensitivity/noHCPROwerktest.tif]

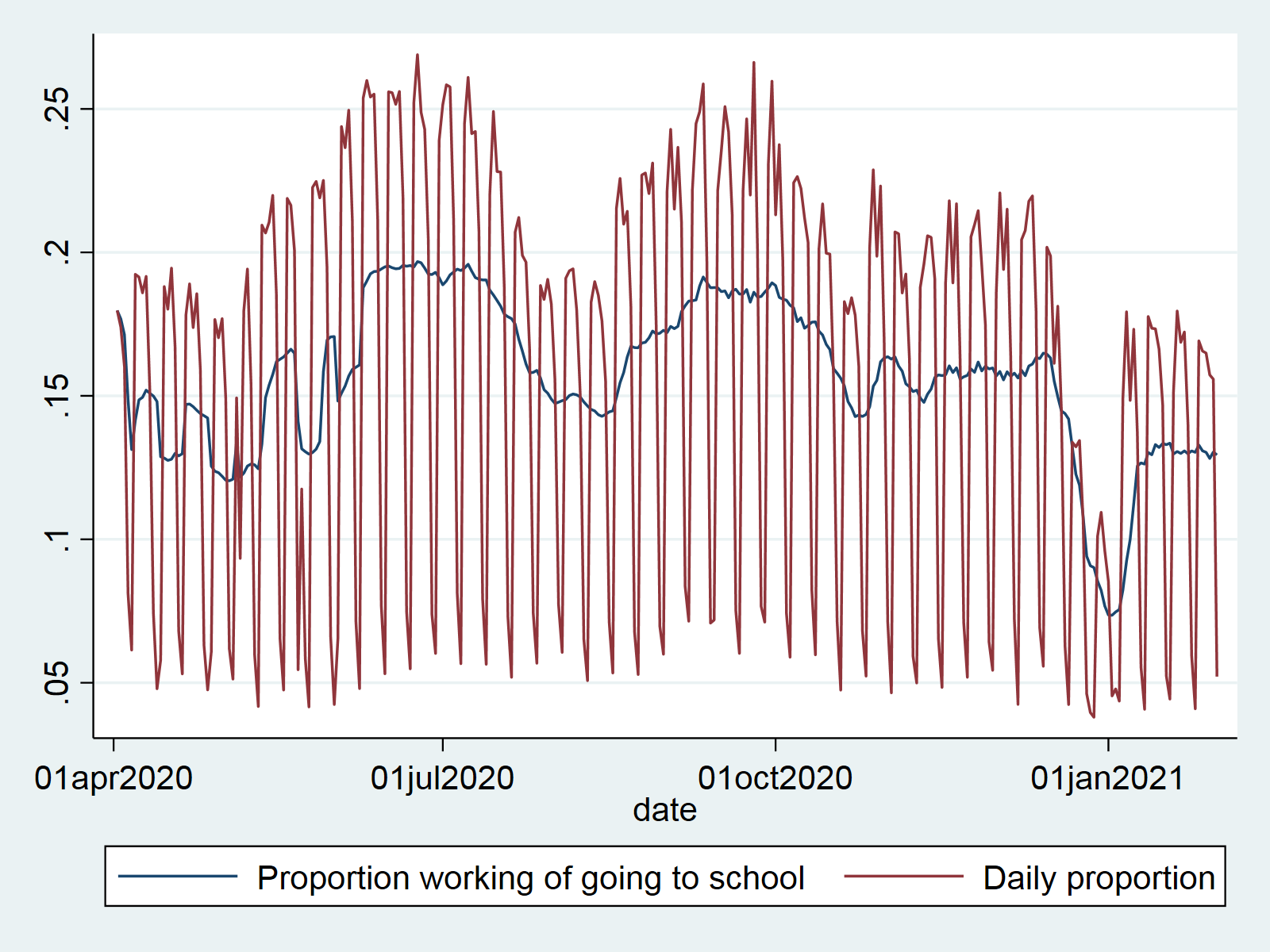

Supplement: S7 File — (ZIP) [file pone.0253566.s023.zip › sensitivity/noHCPROWerktime.tif]

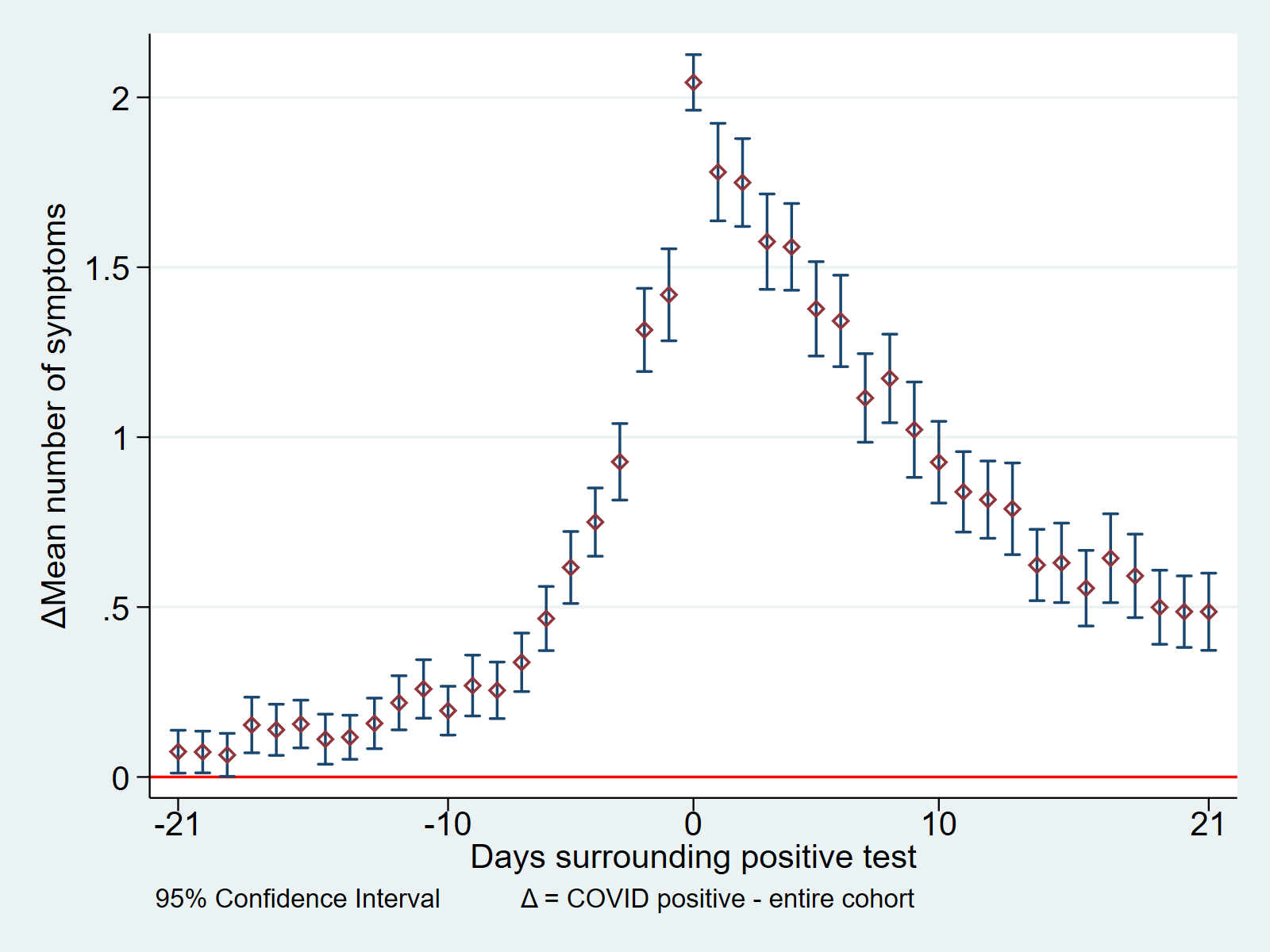

Supplement: S7 File — (ZIP) [file pone.0253566.s023.zip › sensitivity/noLoyalaantalsymp.tif]

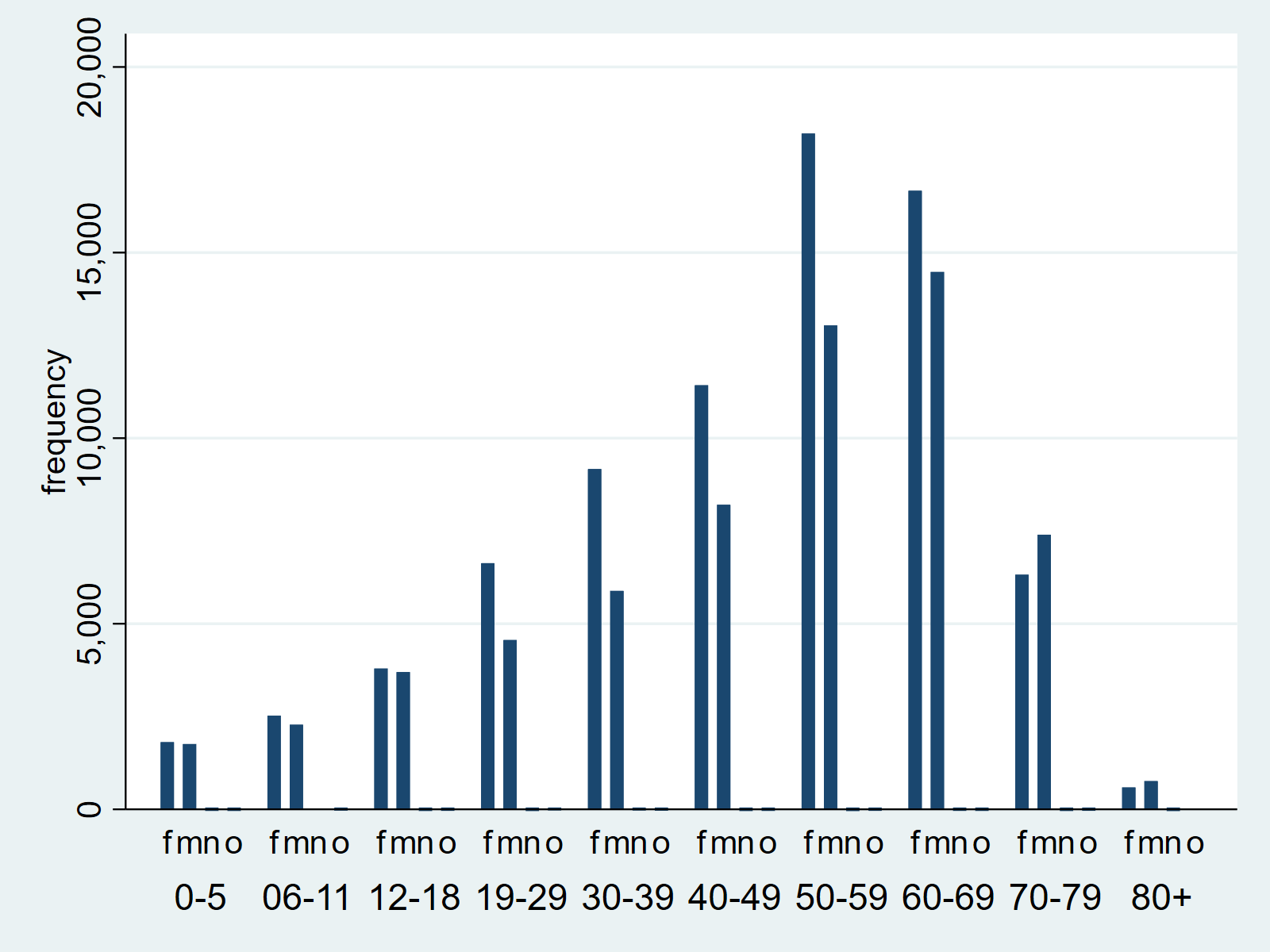

Supplement: S7 File — (ZIP) [file pone.0253566.s023.zip › sensitivity/noLoyalagegenderobs.tif]

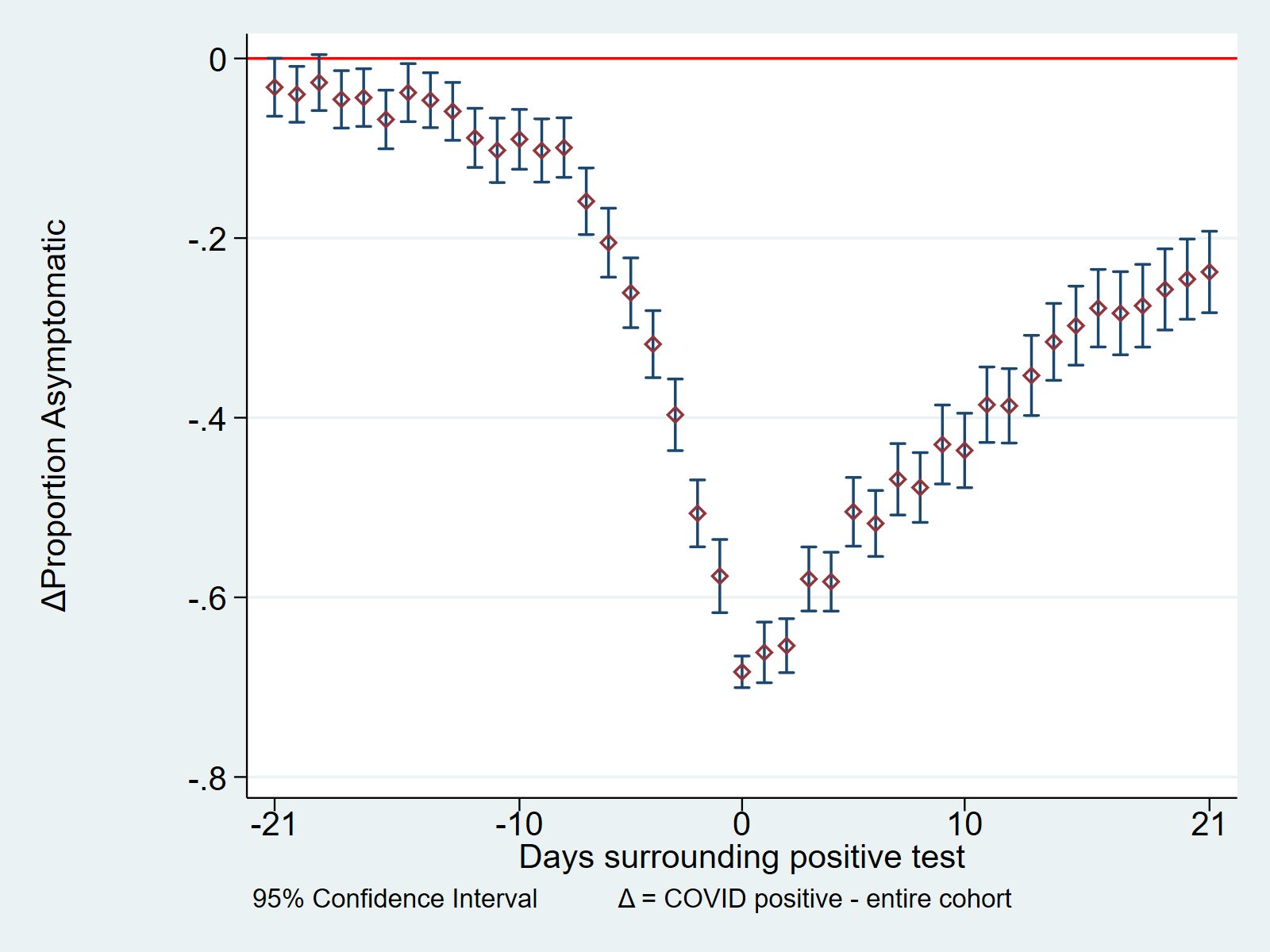

Supplement: S7 File — (ZIP) [file pone.0253566.s023.zip › sensitivity/noLoyalasymp.tif]

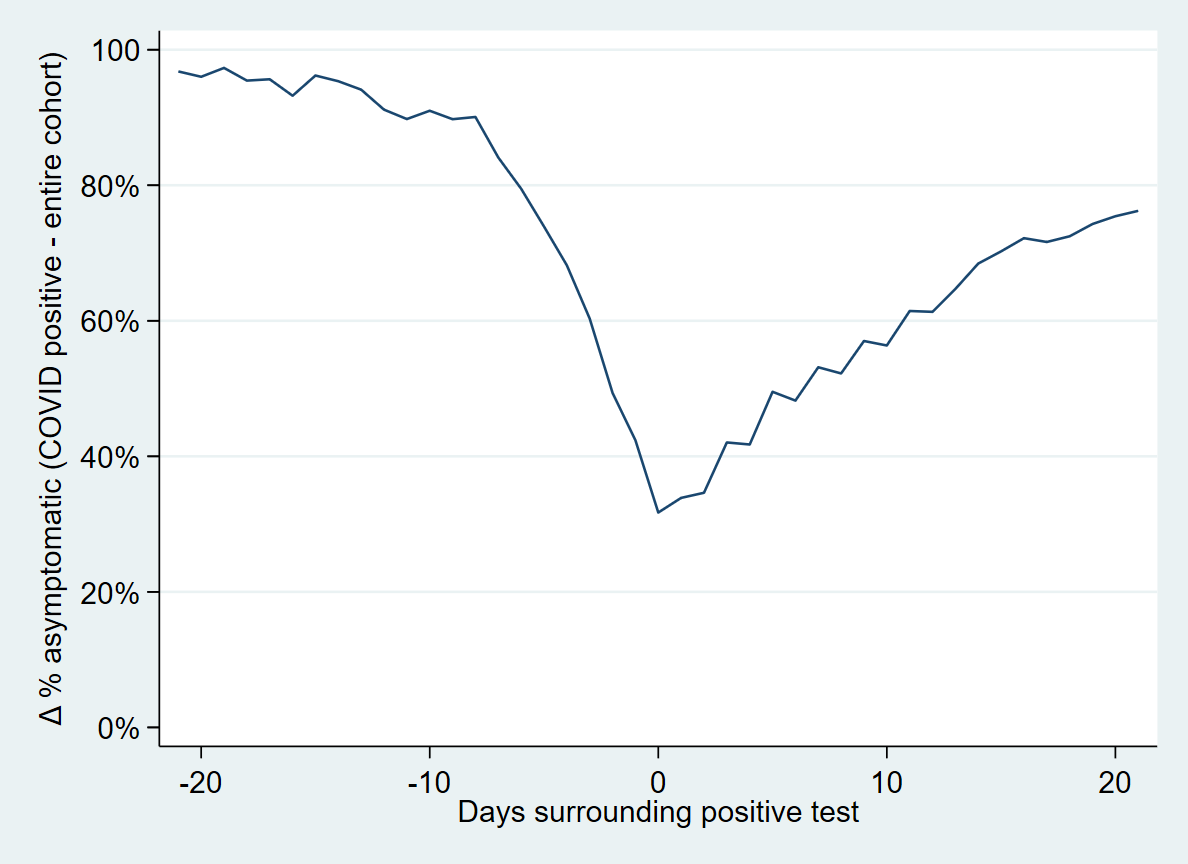

Supplement: S7 File — (ZIP) [file pone.0253566.s023.zip › sensitivity/noLoyalasymptest.tif]

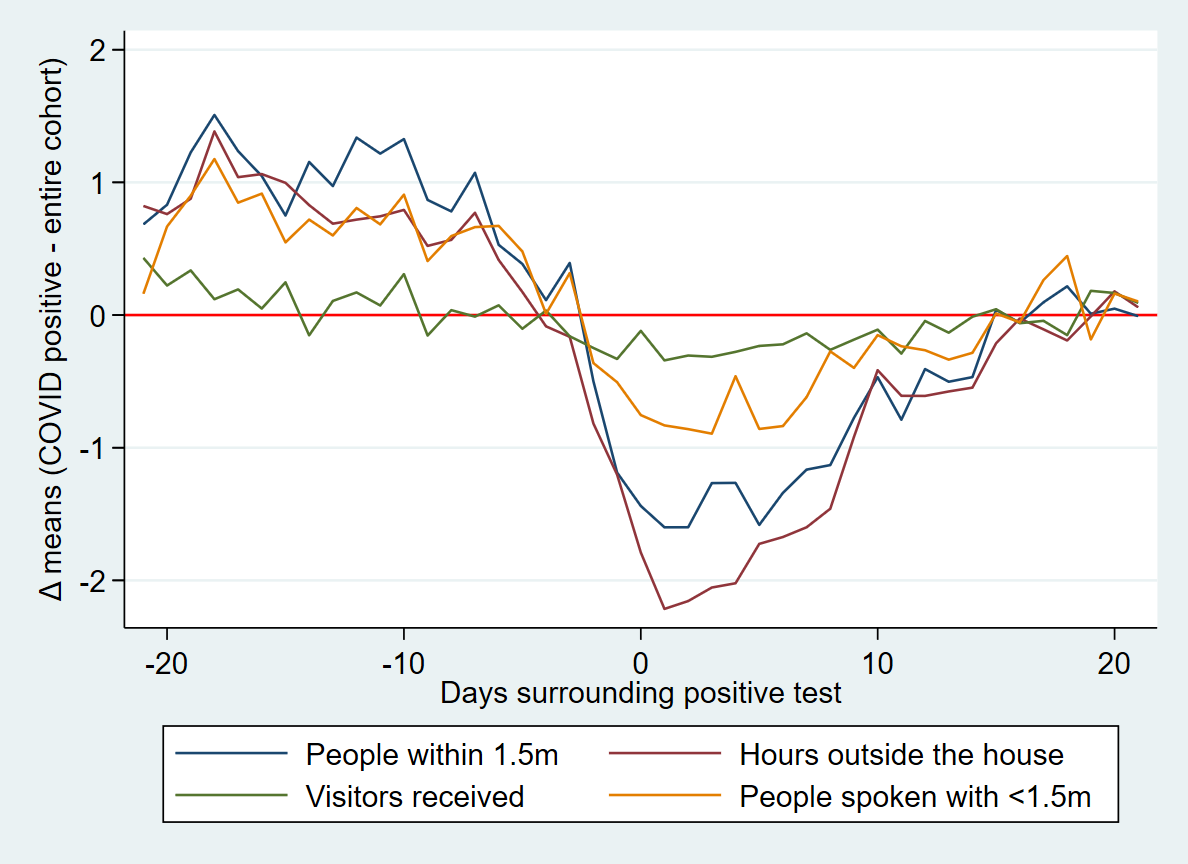

Supplement: S7 File — (ZIP) [file pone.0253566.s023.zip › sensitivity/noLoyalbehavetest.tif]

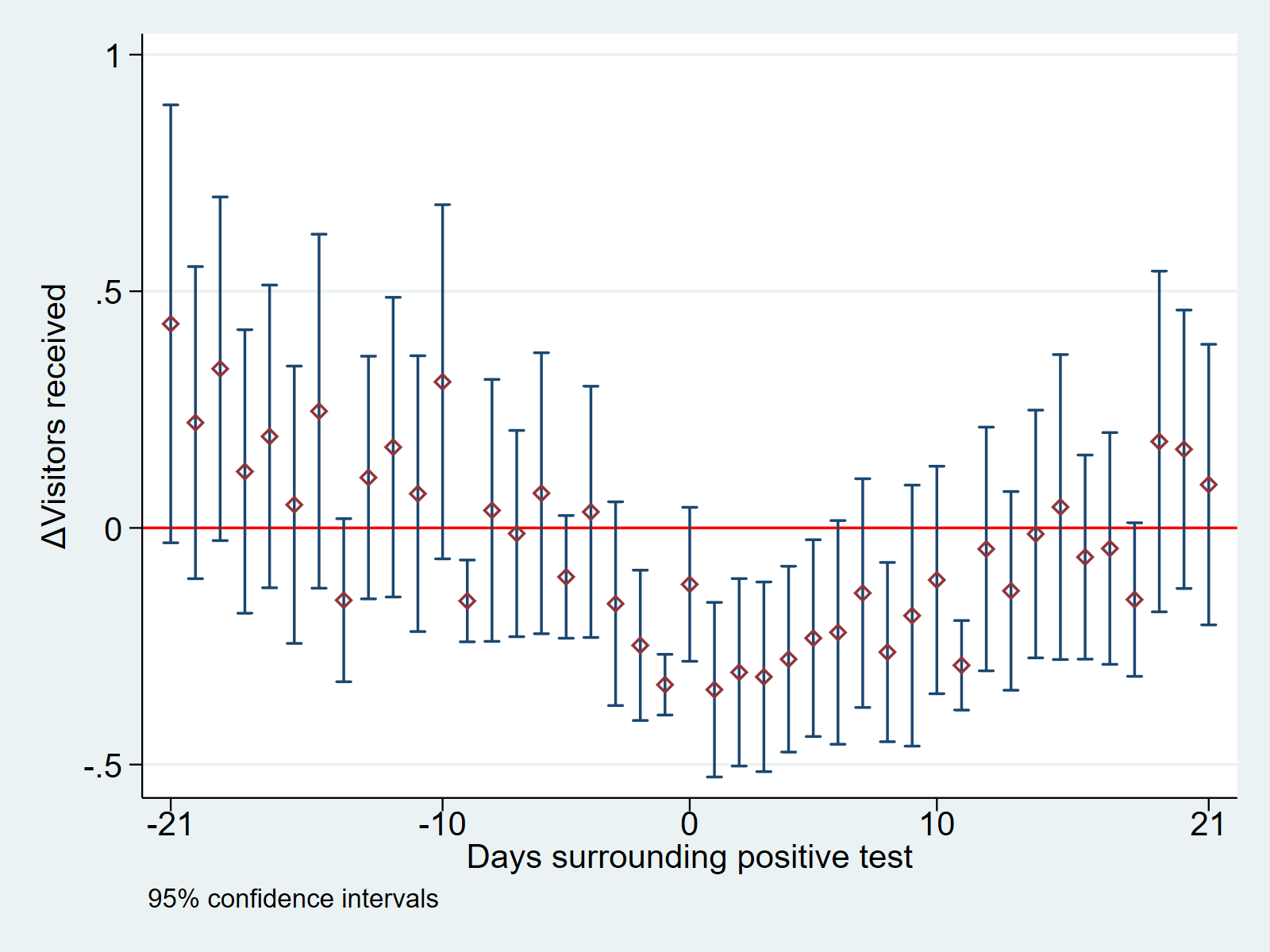

Supplement: S7 File — (ZIP) [file pone.0253566.s023.zip › sensitivity/noLoyalbezoek.tif]

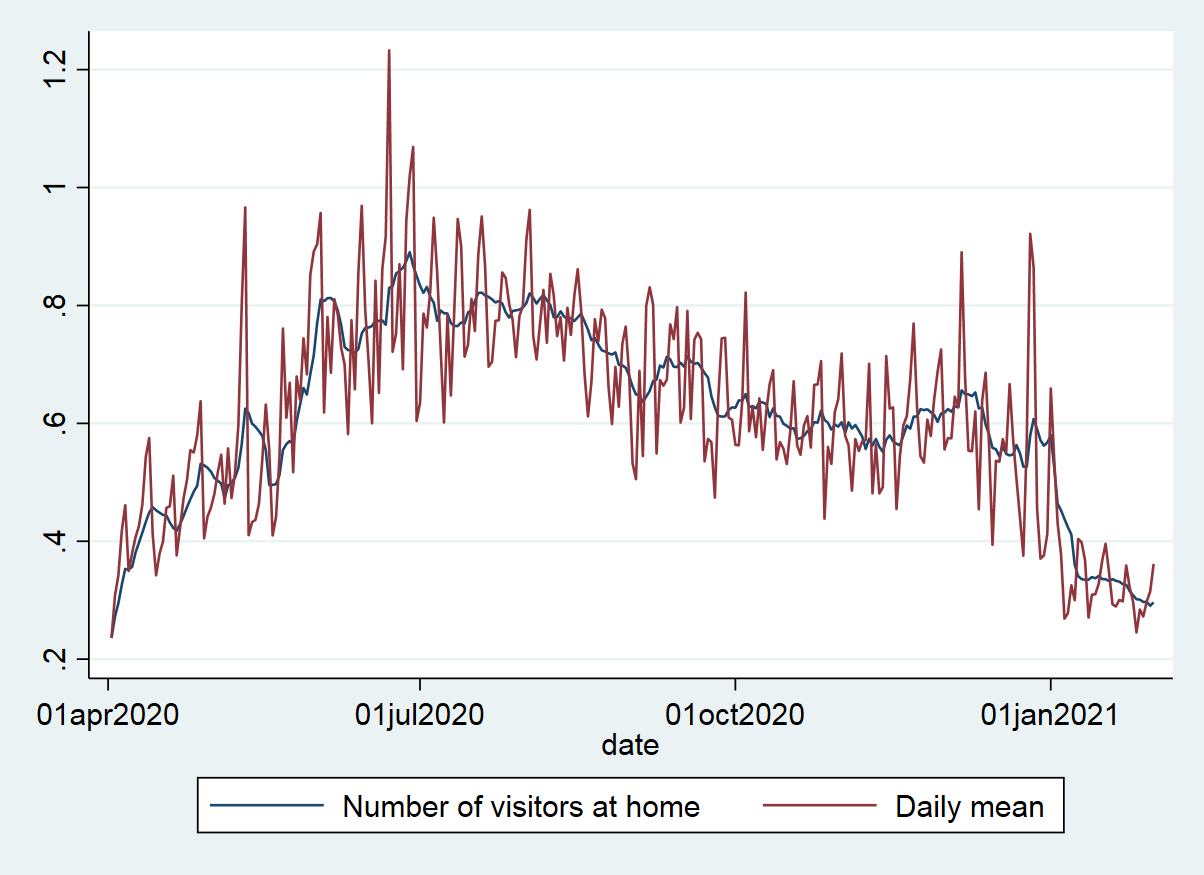

Supplement: S7 File — (ZIP) [file pone.0253566.s023.zip › sensitivity/noLoyalBezoektime.tif]

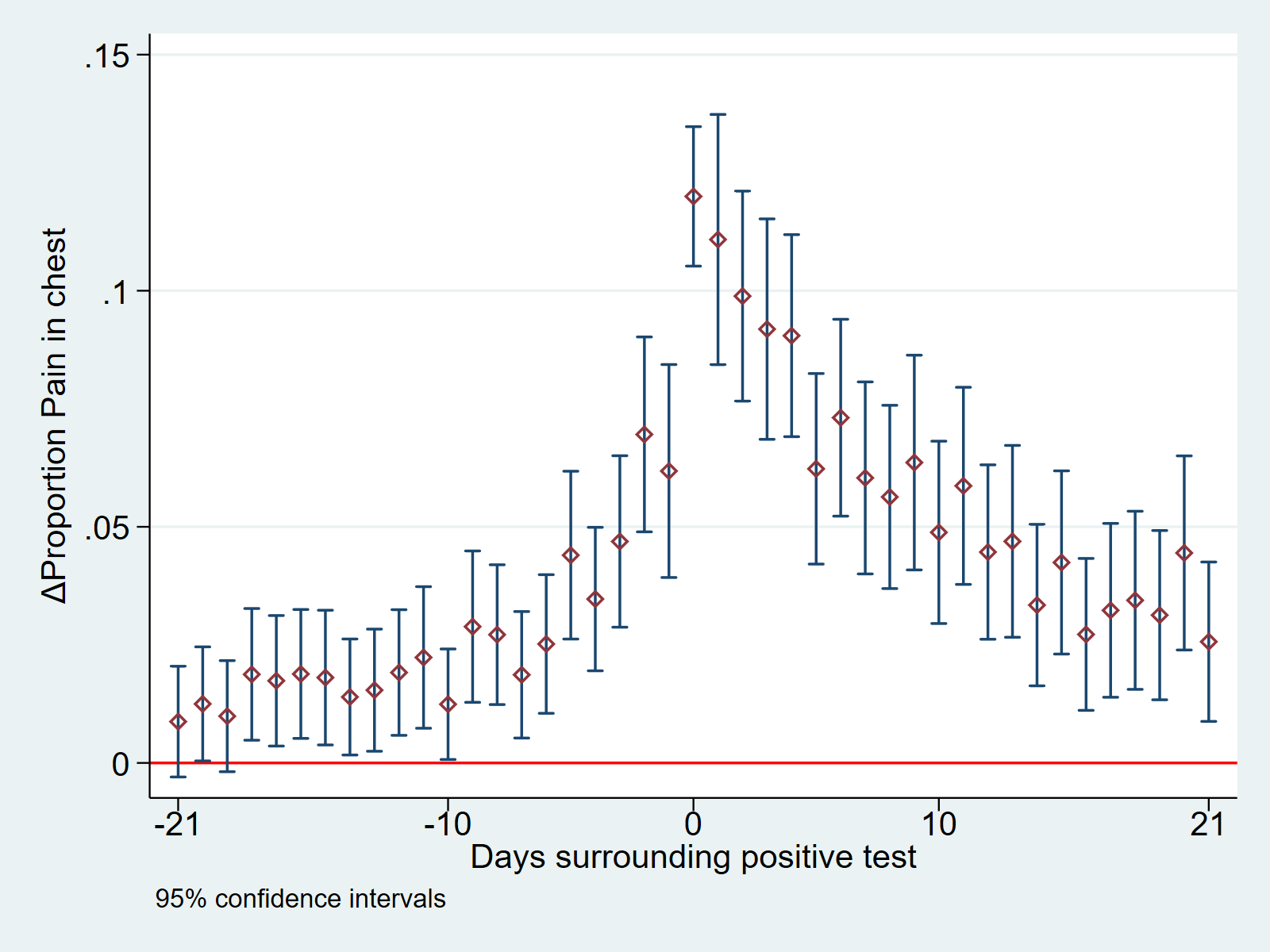

Supplement: S7 File — (ZIP) [file pone.0253566.s023.zip › sensitivity/noLoyalborstkas.tif]

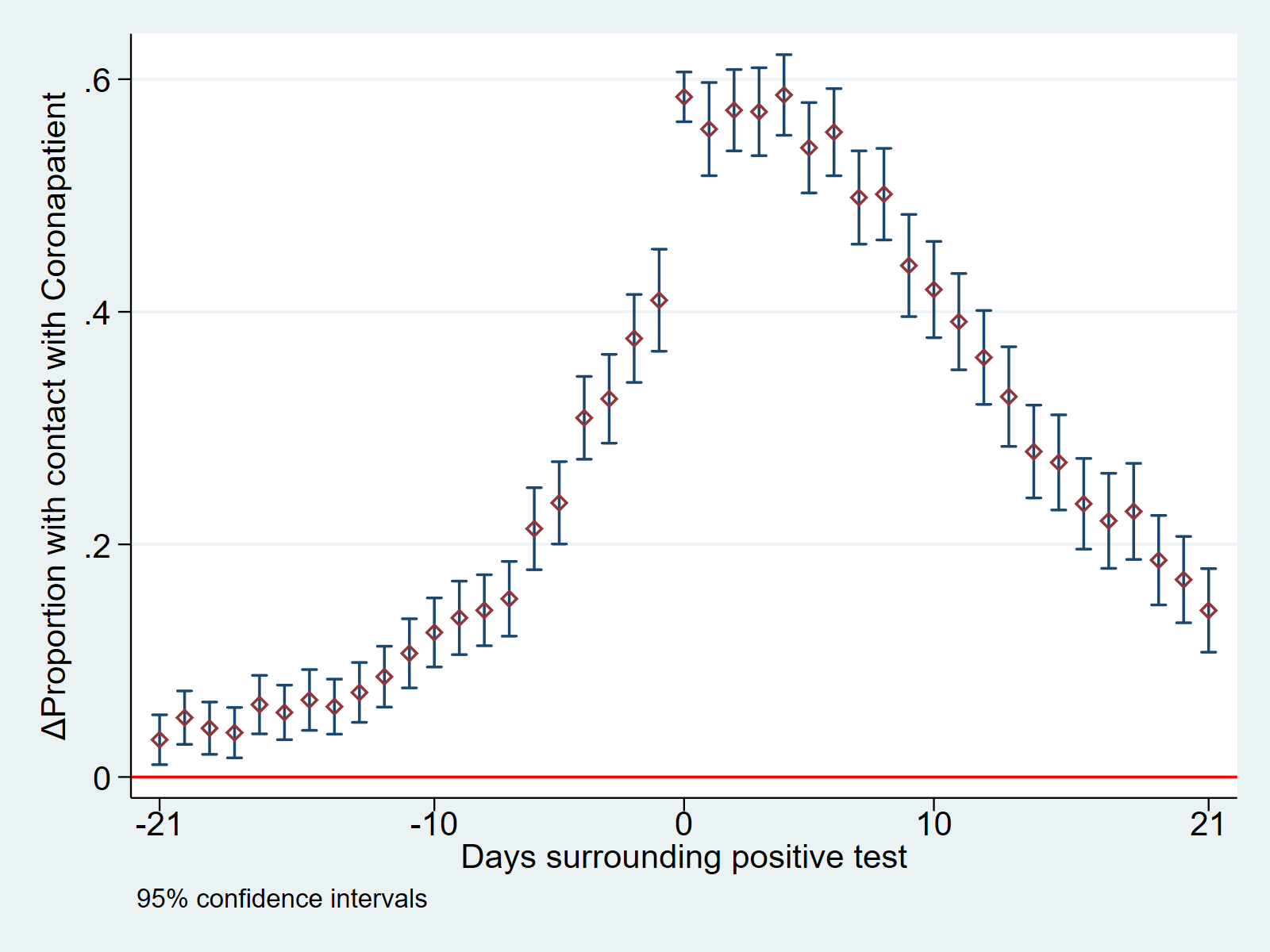

Supplement: S7 File — (ZIP) [file pone.0253566.s023.zip › sensitivity/noLoyalcontact.tif]

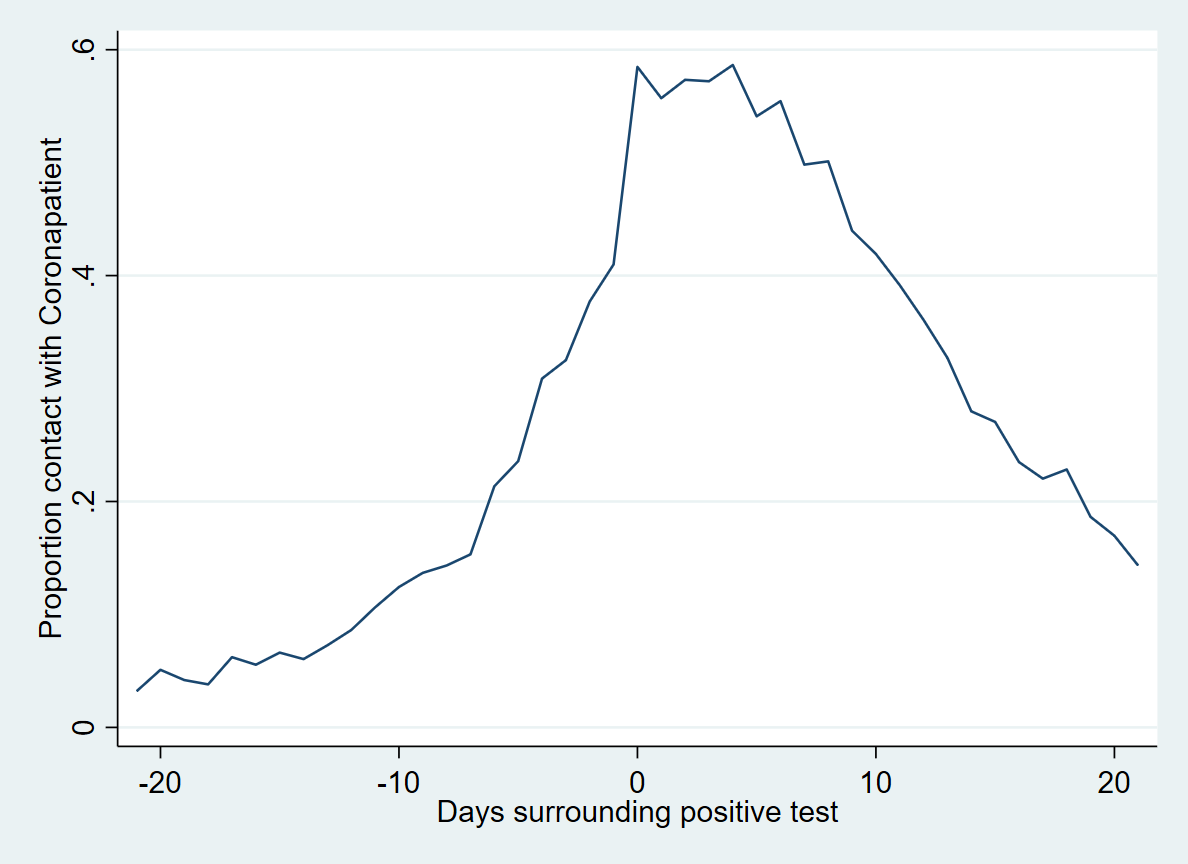

Supplement: S7 File — (ZIP) [file pone.0253566.s023.zip › sensitivity/noLoyalcontacttest.tif]

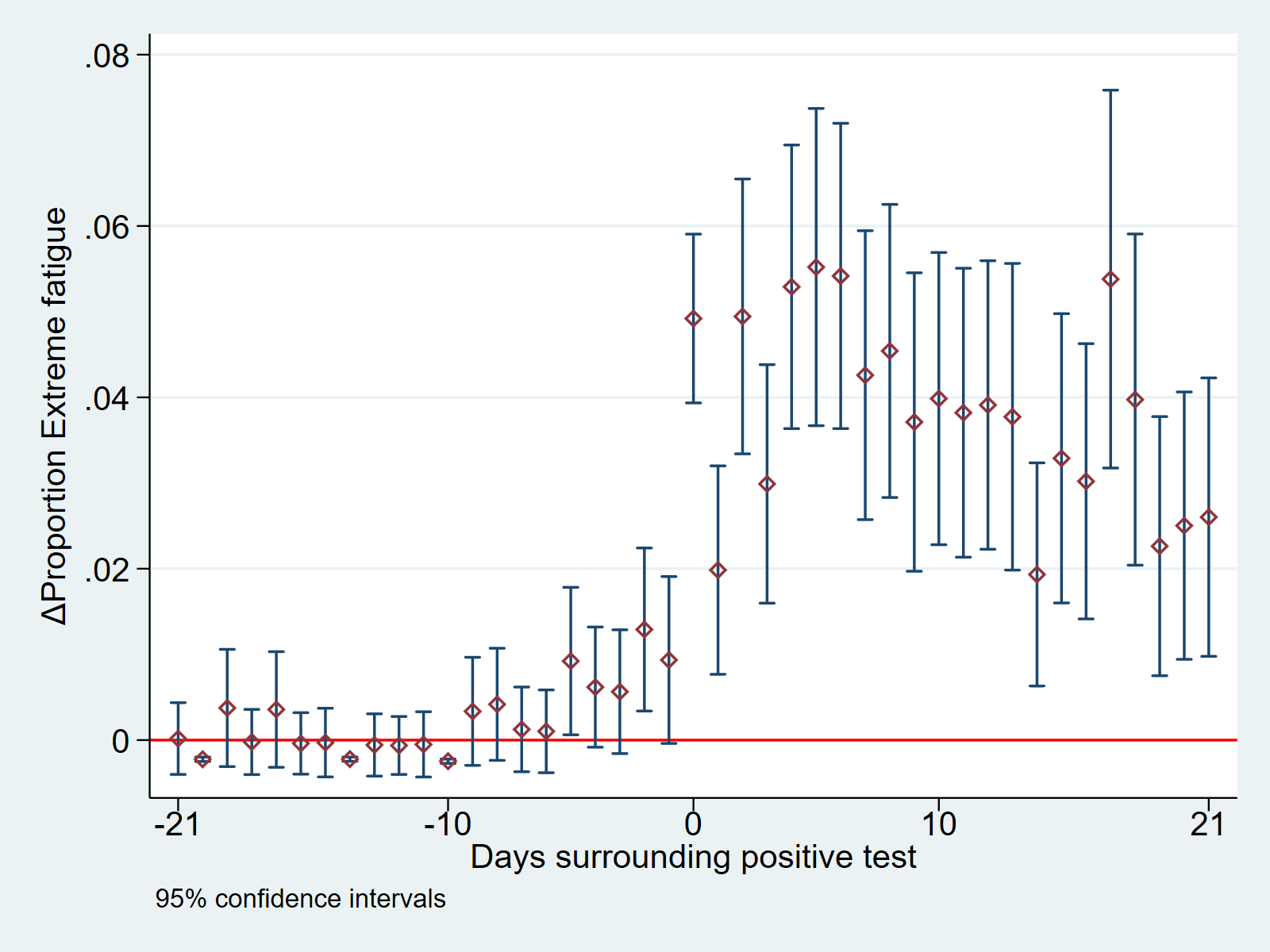

Supplement: S7 File — (ZIP) [file pone.0253566.s023.zip › sensitivity/noLoyalextreemvermoeidheid.tif]

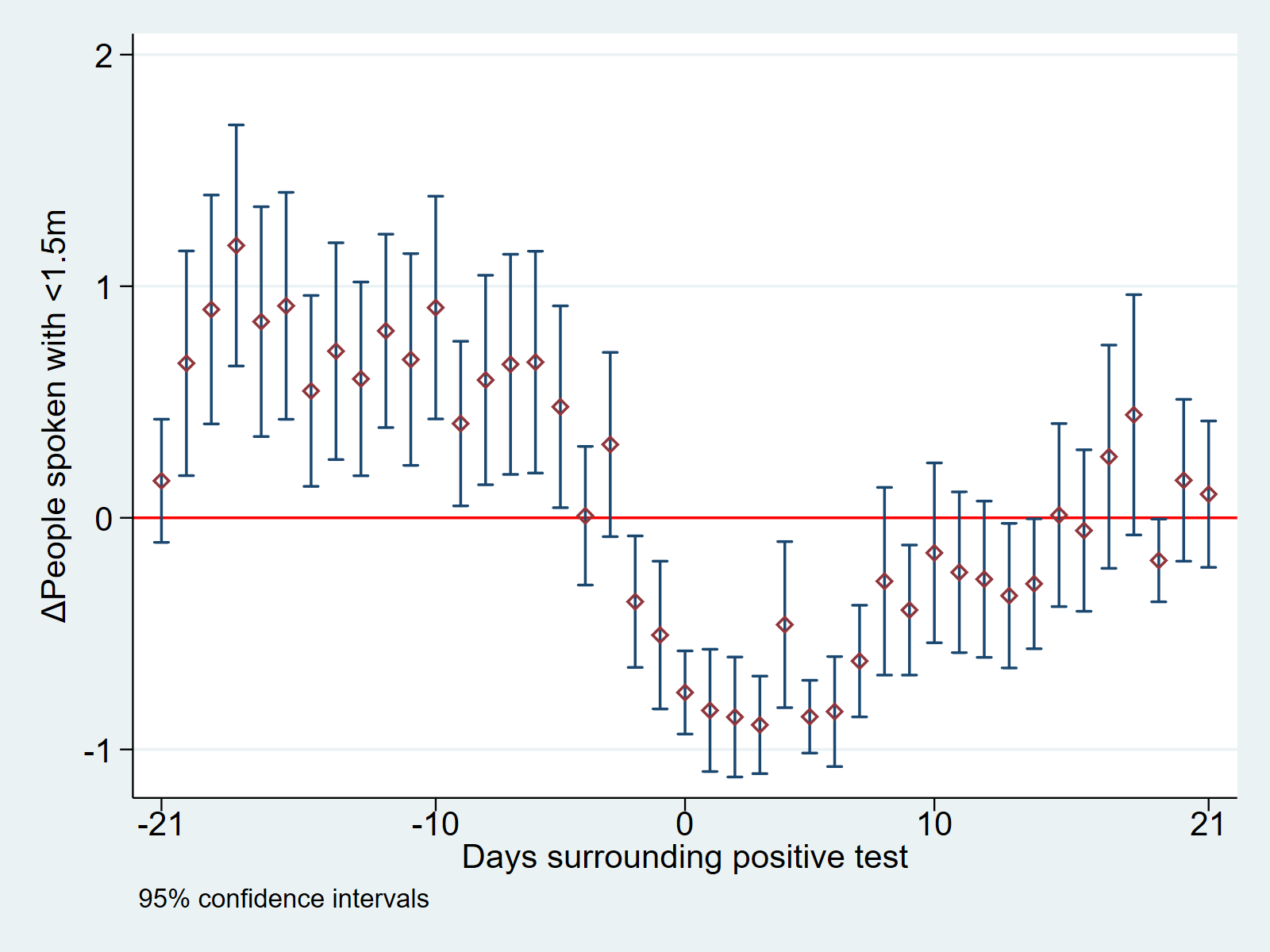

Supplement: S7 File — (ZIP) [file pone.0253566.s023.zip › sensitivity/noLoyalgesproken.tif]

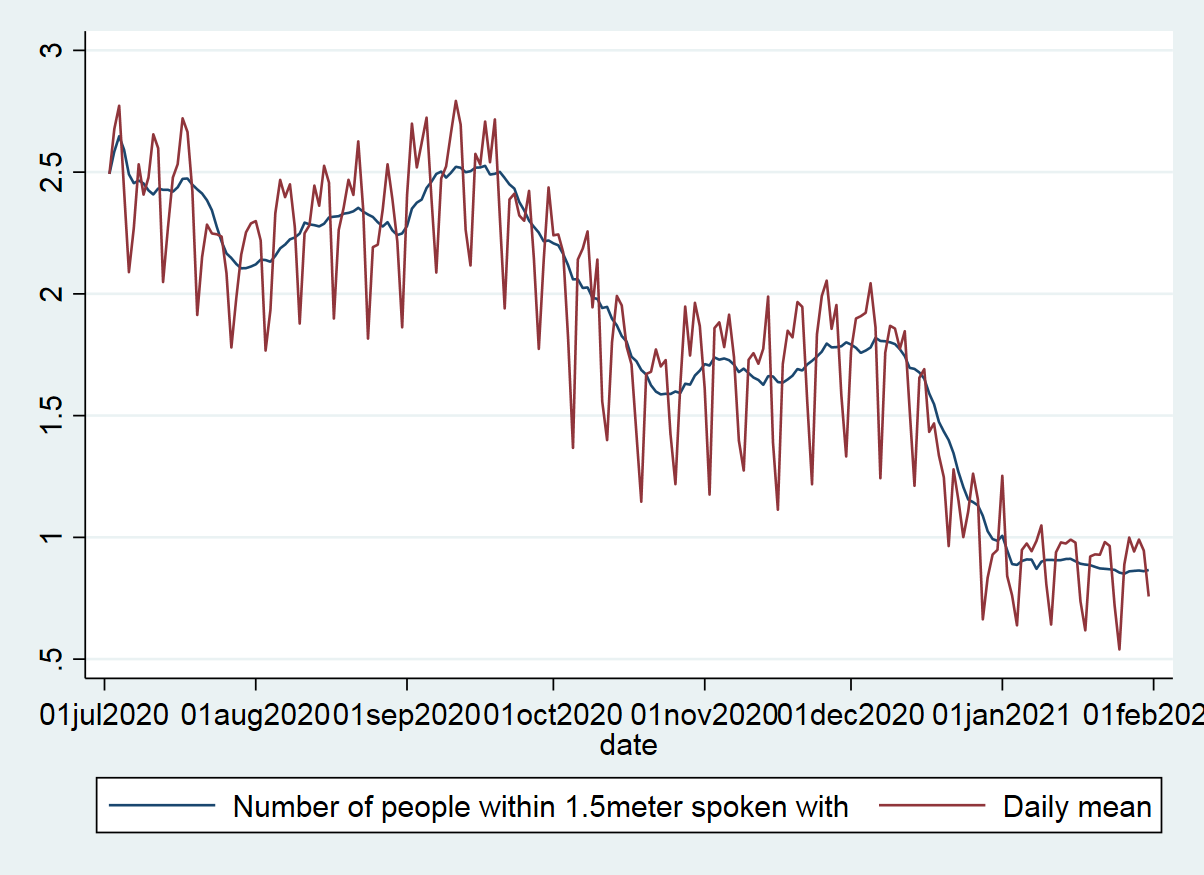

Supplement: S7 File — (ZIP) [file pone.0253566.s023.zip › sensitivity/noLoyalGesprokentime.tif]

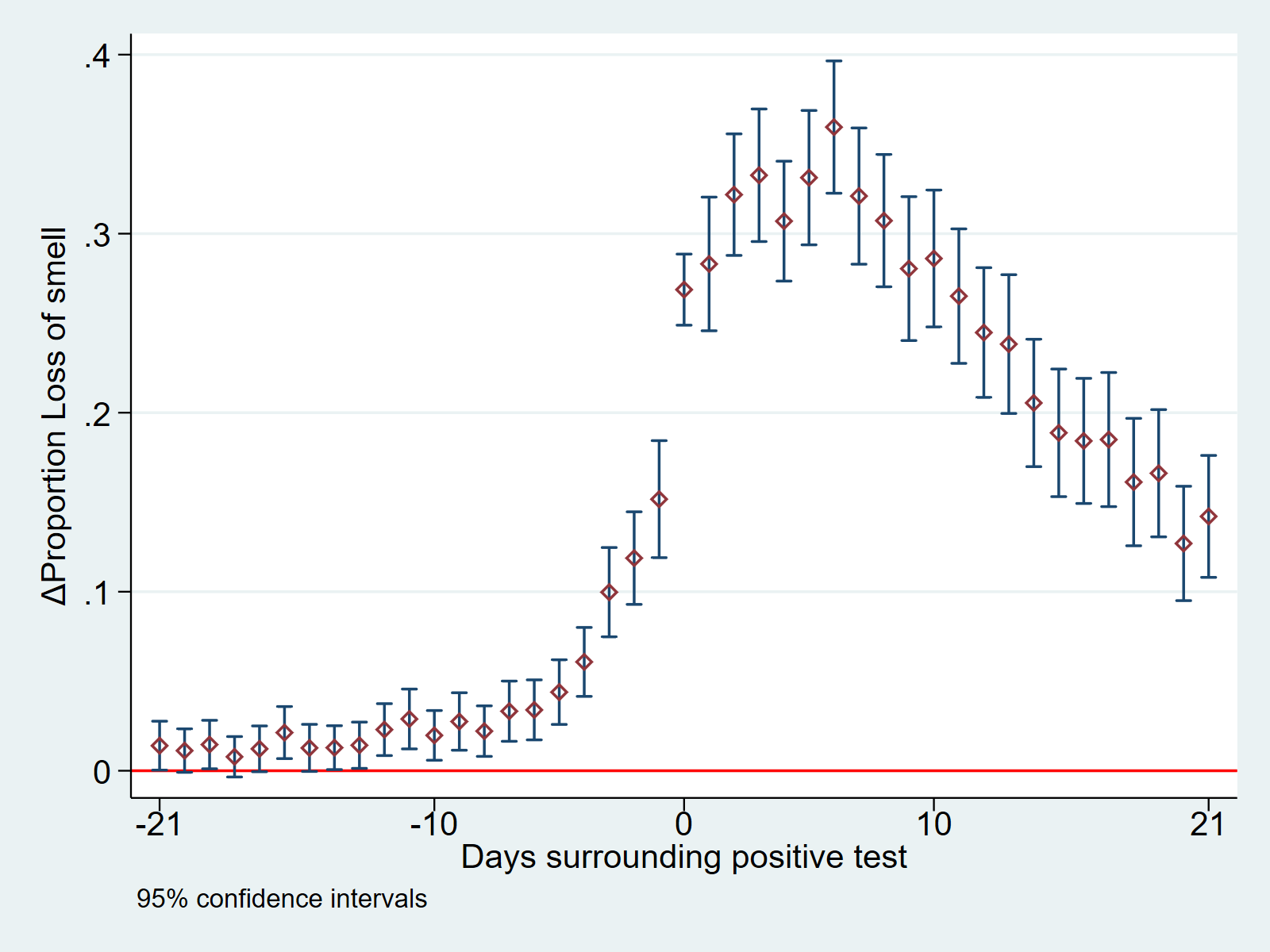

Supplement: S7 File — (ZIP) [file pone.0253566.s023.zip › sensitivity/noLoyalgeur.tif]

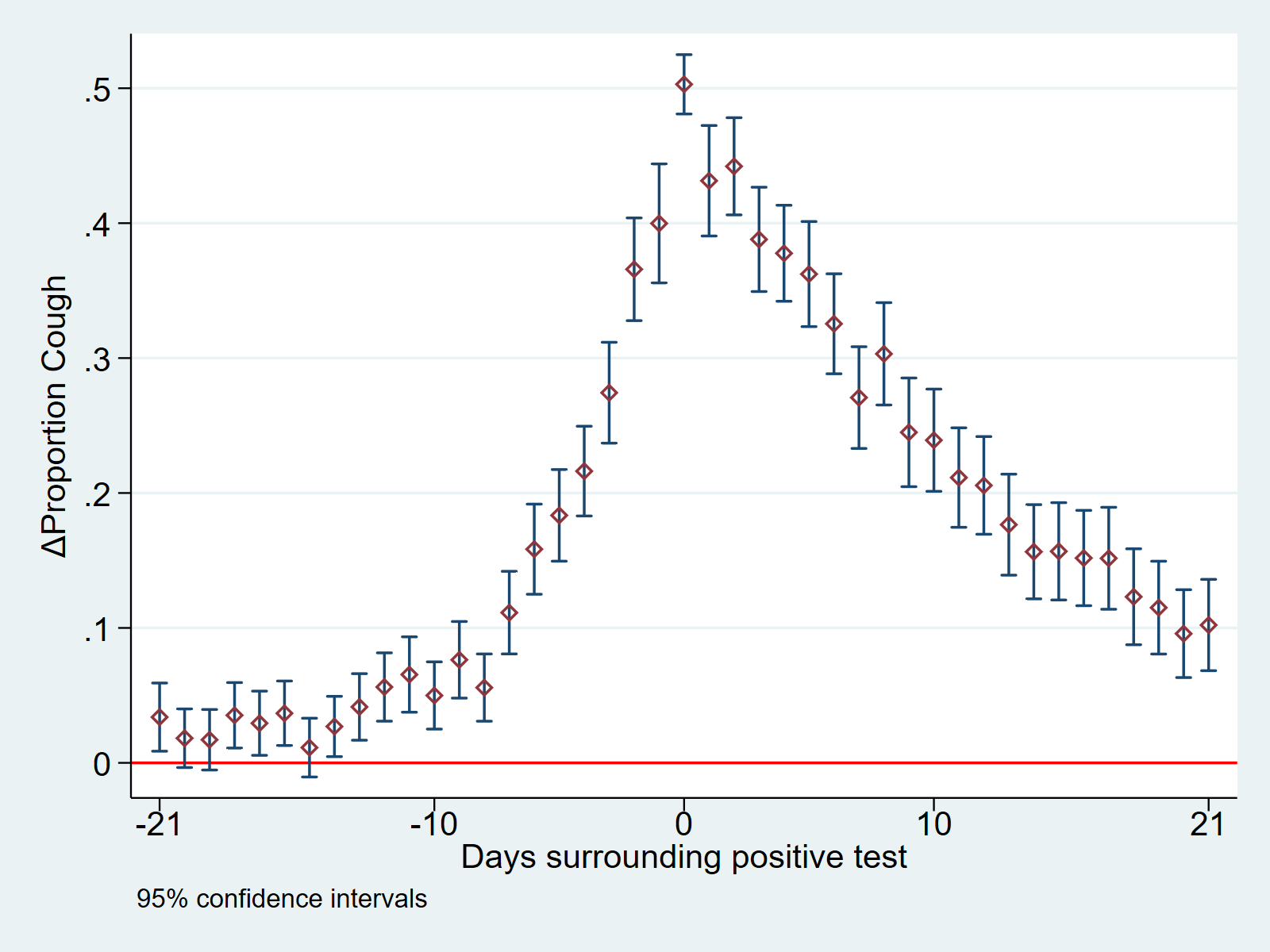

Supplement: S7 File — (ZIP) [file pone.0253566.s023.zip › sensitivity/noLoyalhoesten.tif]

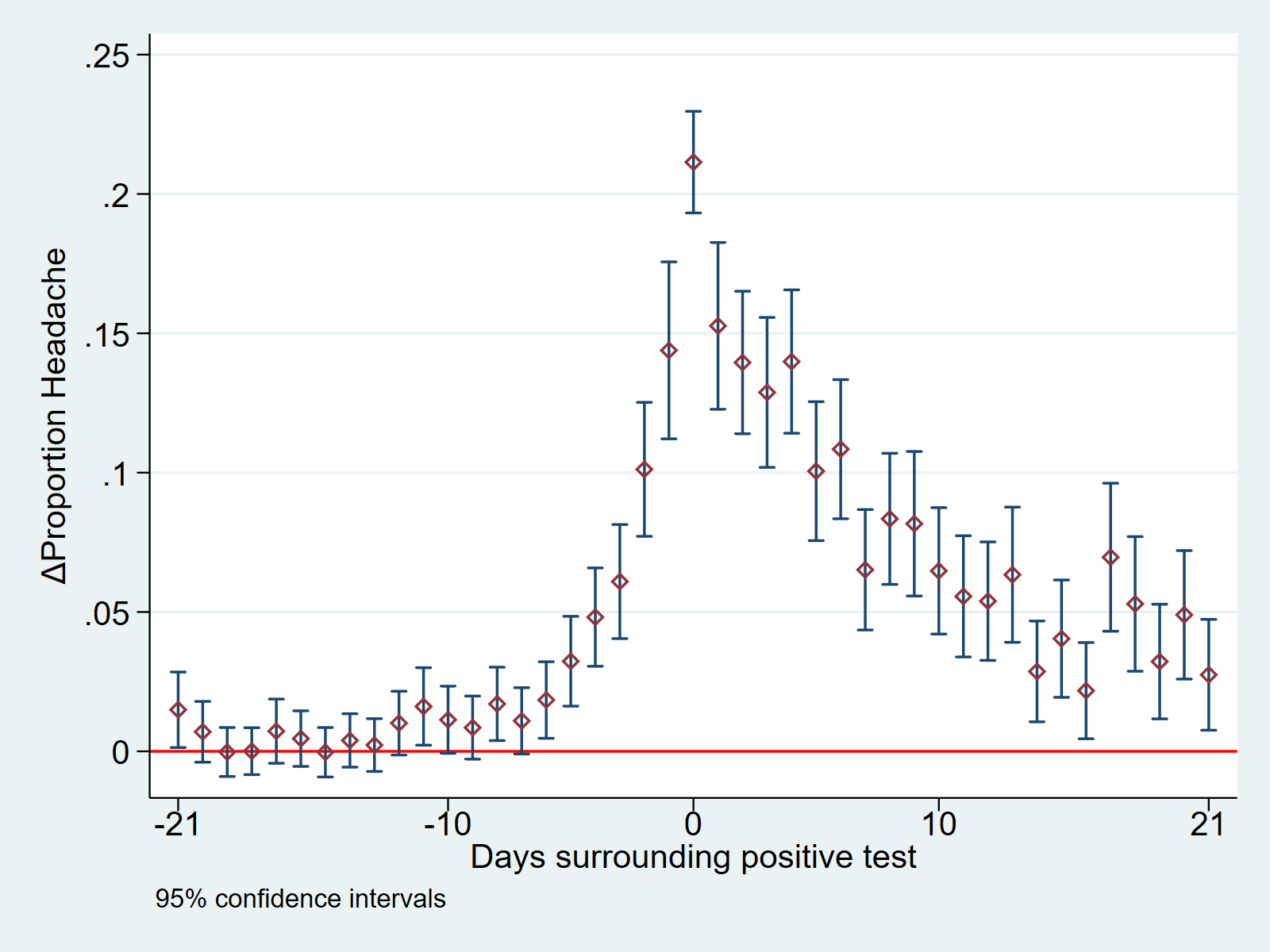

Supplement: S7 File — (ZIP) [file pone.0253566.s023.zip › sensitivity/noLoyalhoofdpijn.tif]

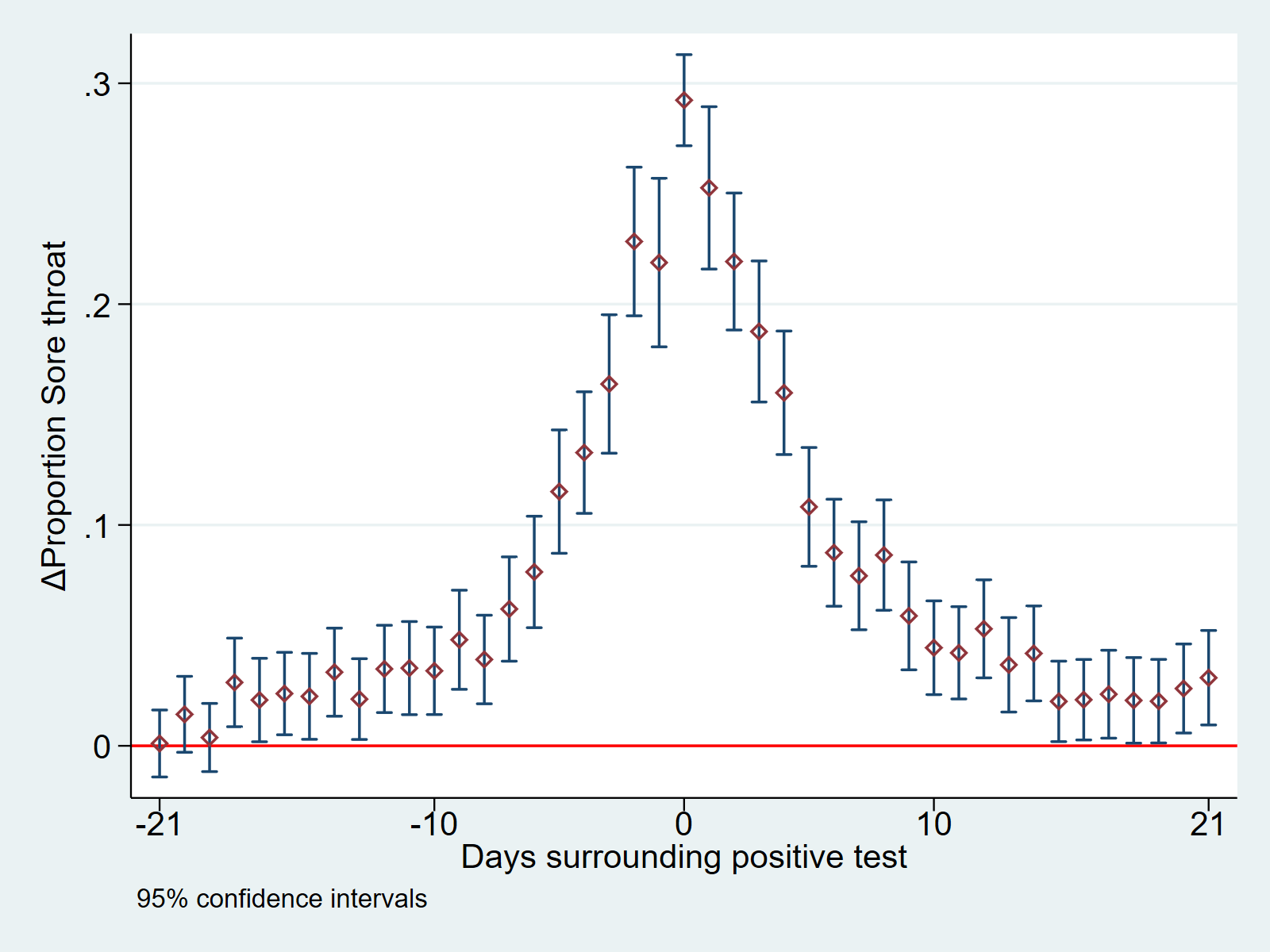

Supplement: S7 File — (ZIP) [file pone.0253566.s023.zip › sensitivity/noLoyalkeelpijn.tif]

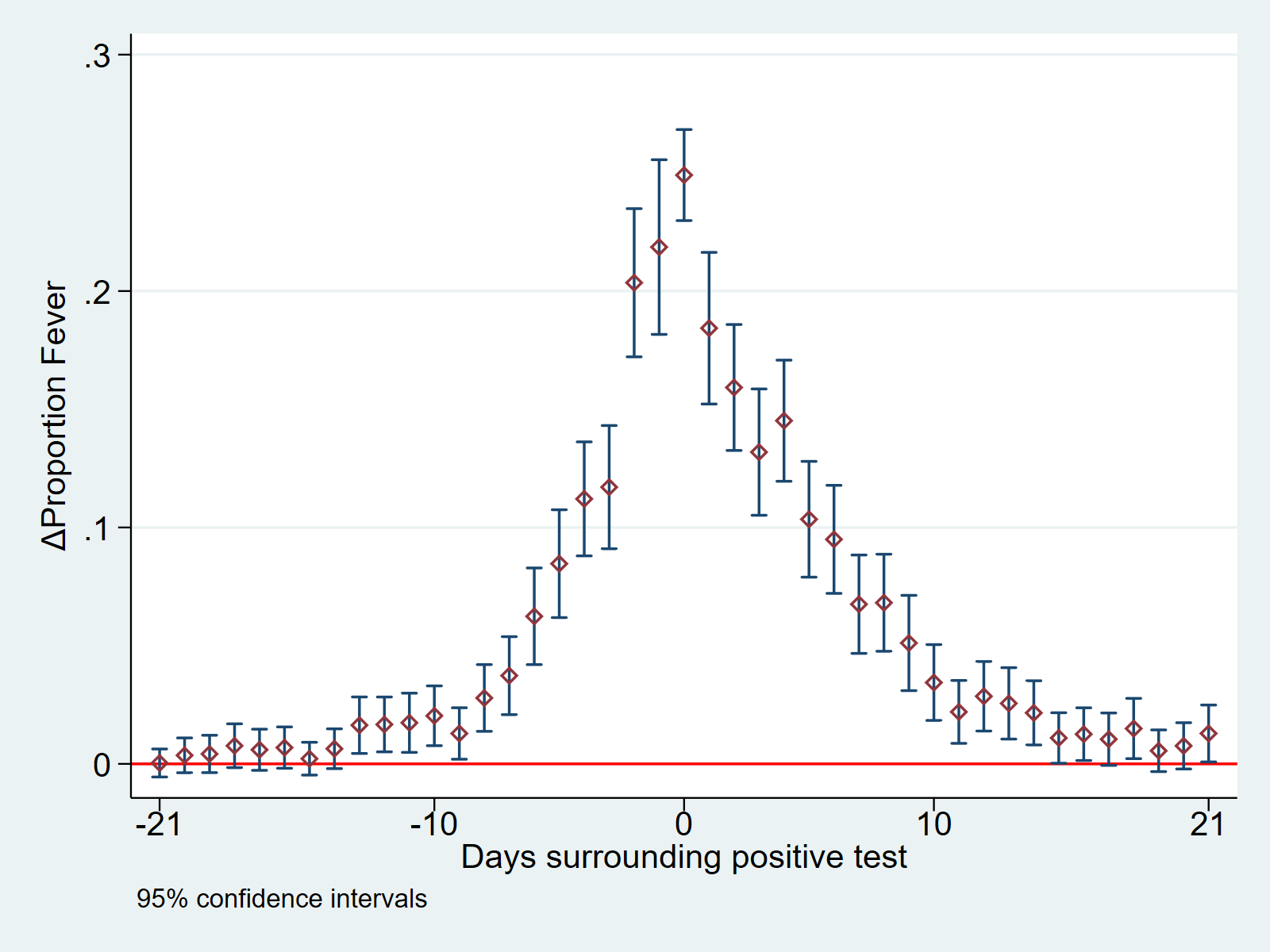

Supplement: S7 File — (ZIP) [file pone.0253566.s023.zip › sensitivity/noLoyalkoorts.tif]

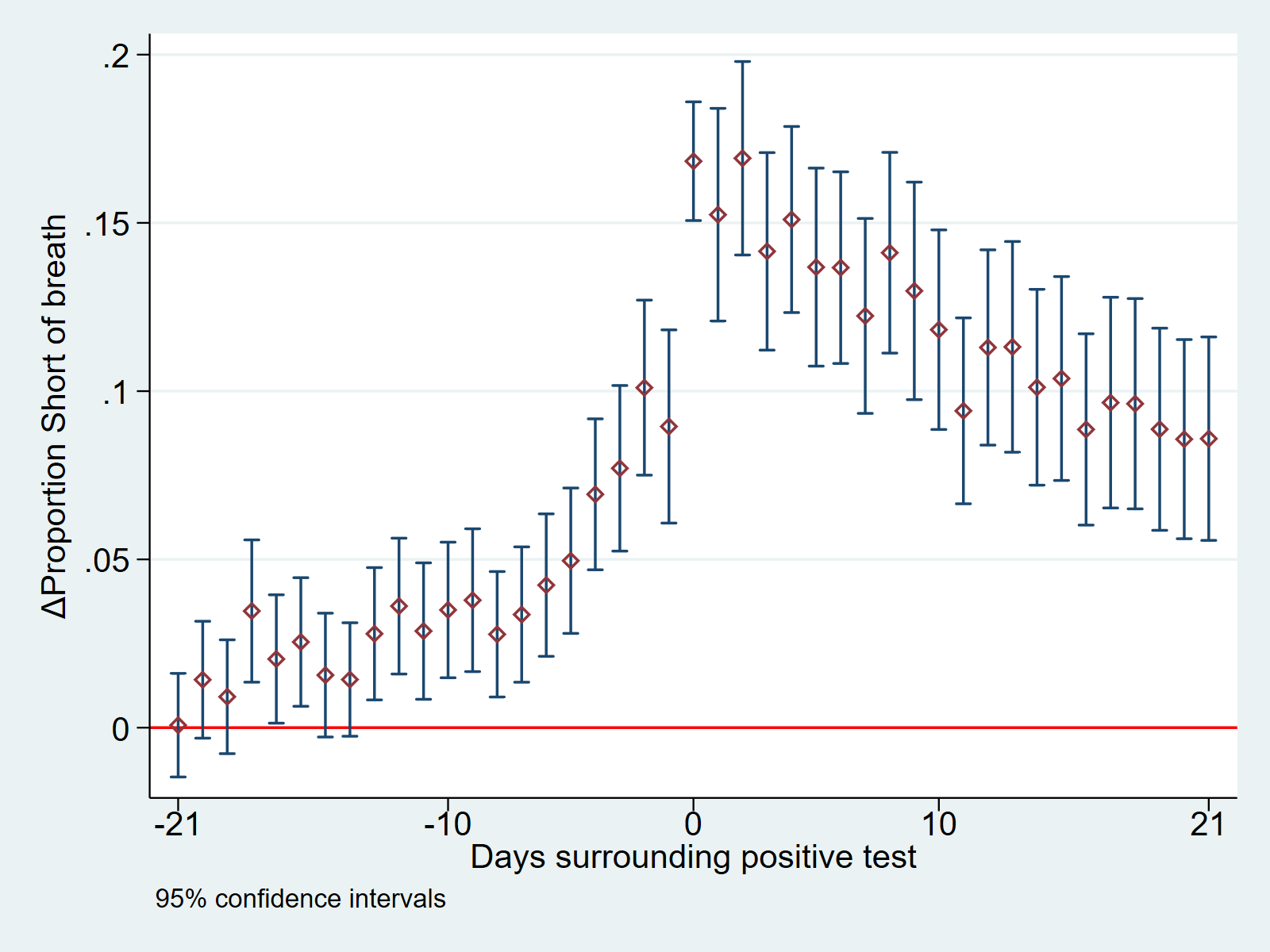

Supplement: S7 File — (ZIP) [file pone.0253566.s023.zip › sensitivity/noLoyalkortademig.tif]

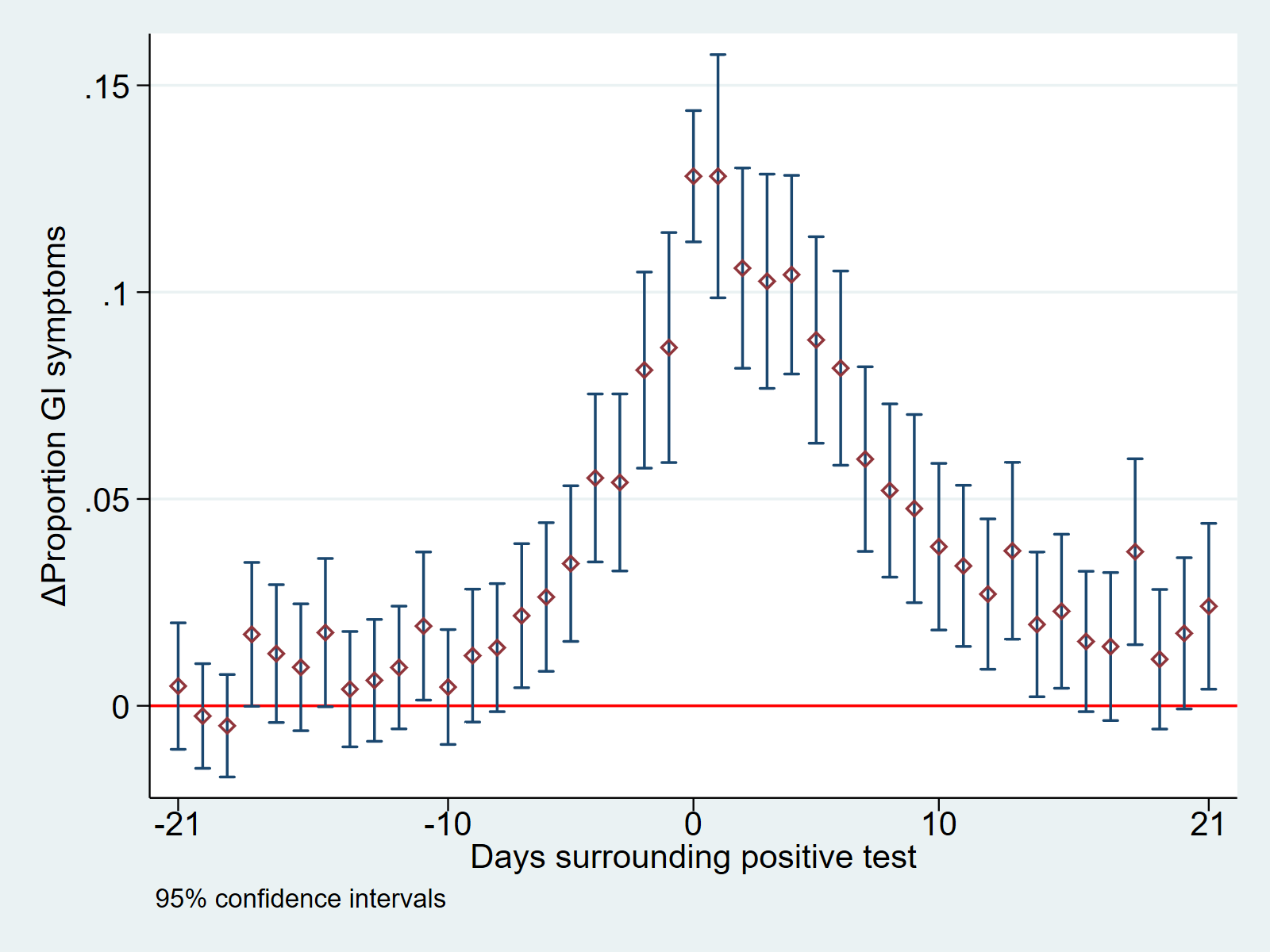

Supplement: S7 File — (ZIP) [file pone.0253566.s023.zip › sensitivity/noLoyalmaagdarm.tif]

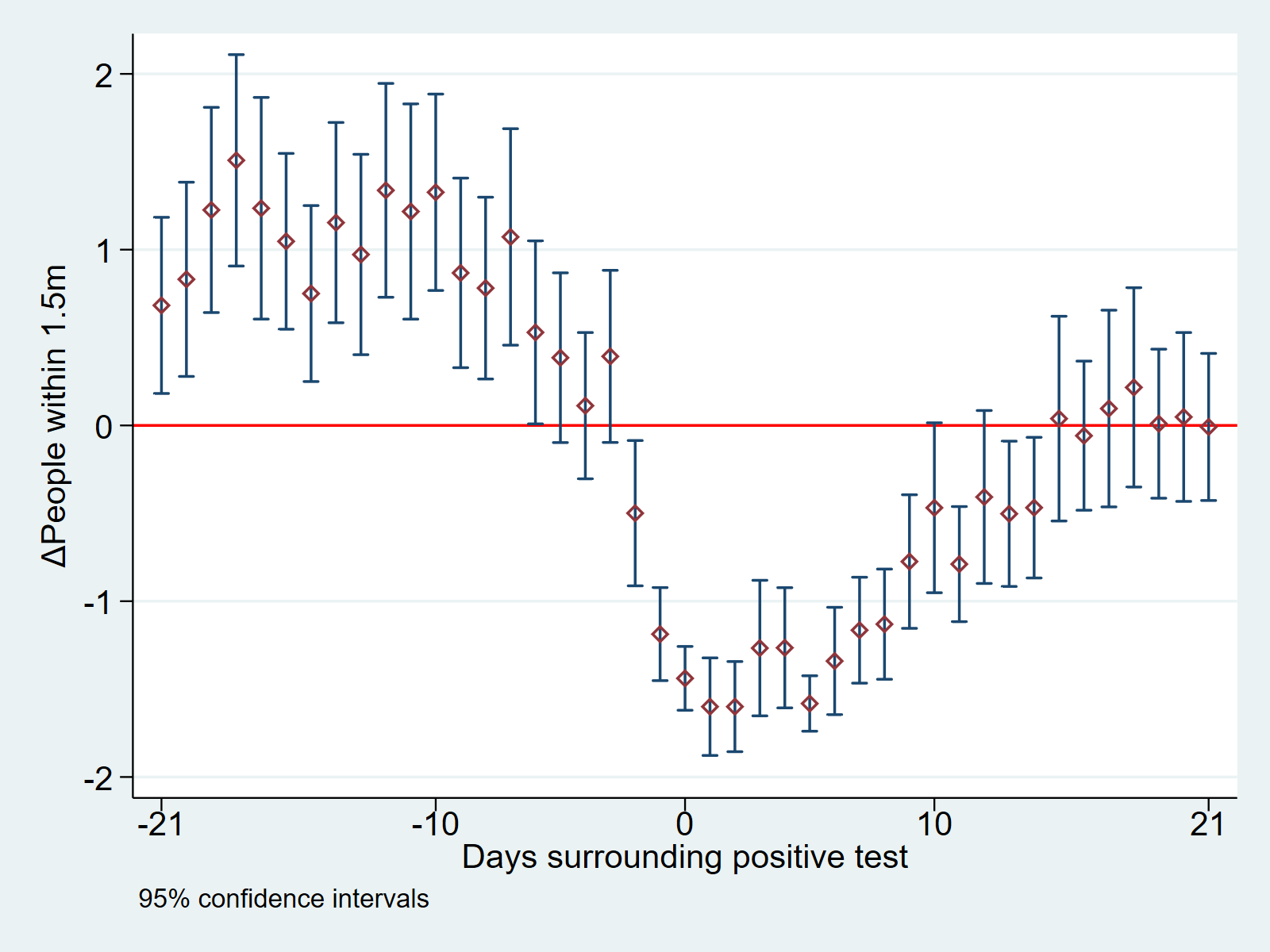

Supplement: S7 File — (ZIP) [file pone.0253566.s023.zip › sensitivity/noLoyalnabij.tif]

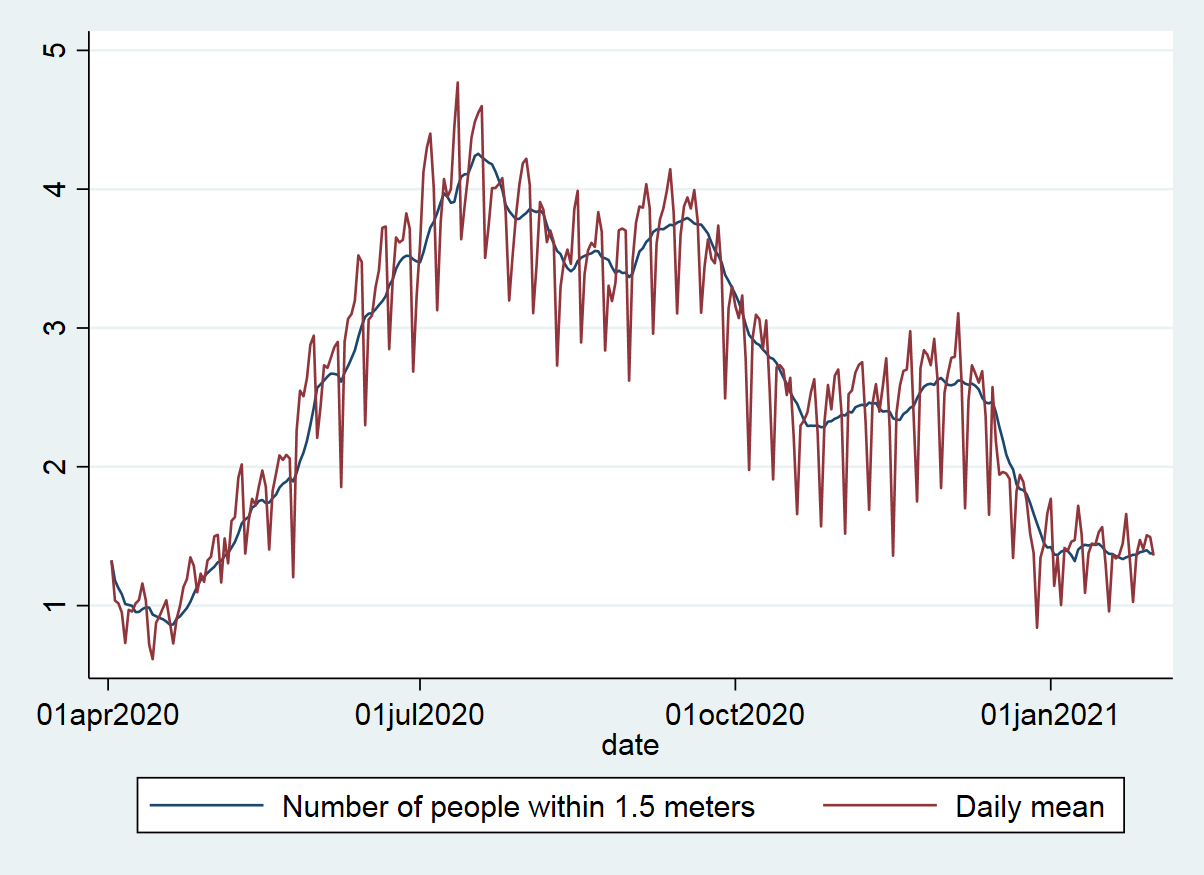

Supplement: S7 File — (ZIP) [file pone.0253566.s023.zip › sensitivity/noLoyalNabijtime.tif]

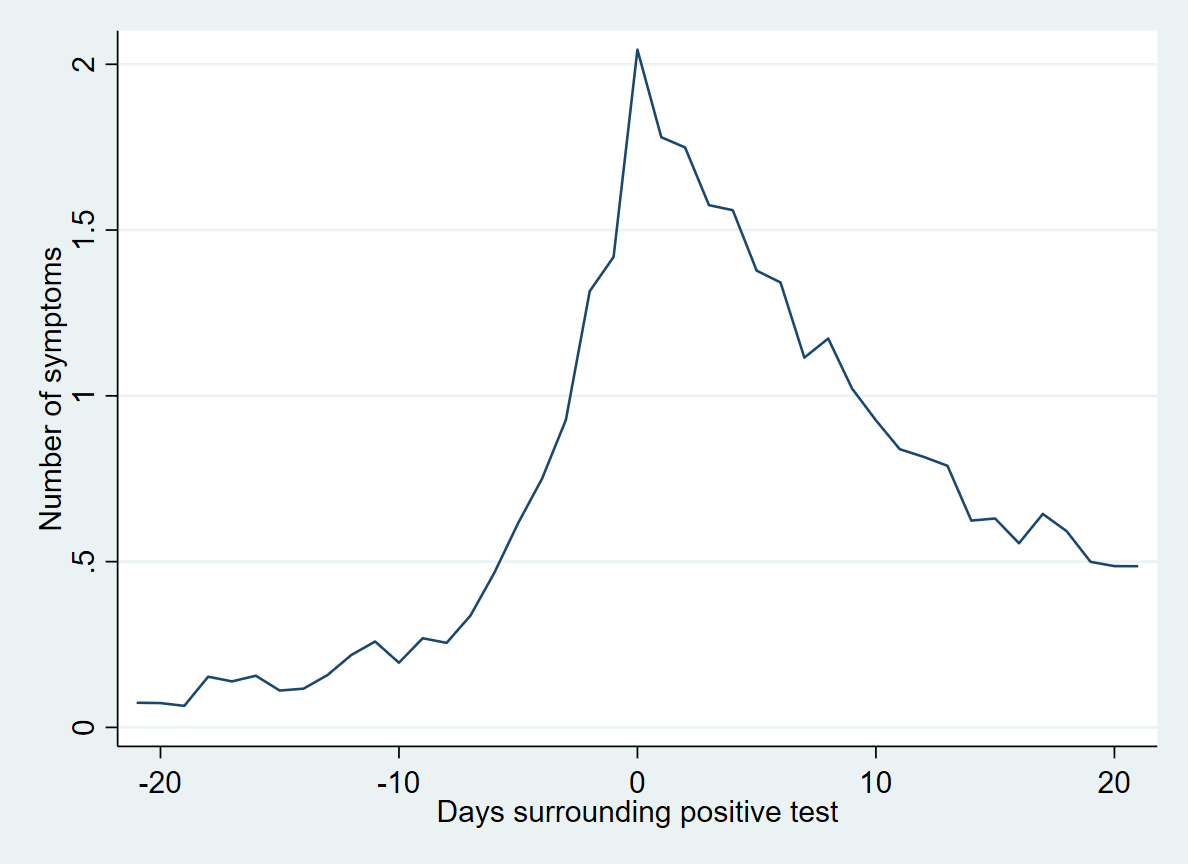

Supplement: S7 File — (ZIP) [file pone.0253566.s023.zip › sensitivity/noLoyalnsymptest.tif]

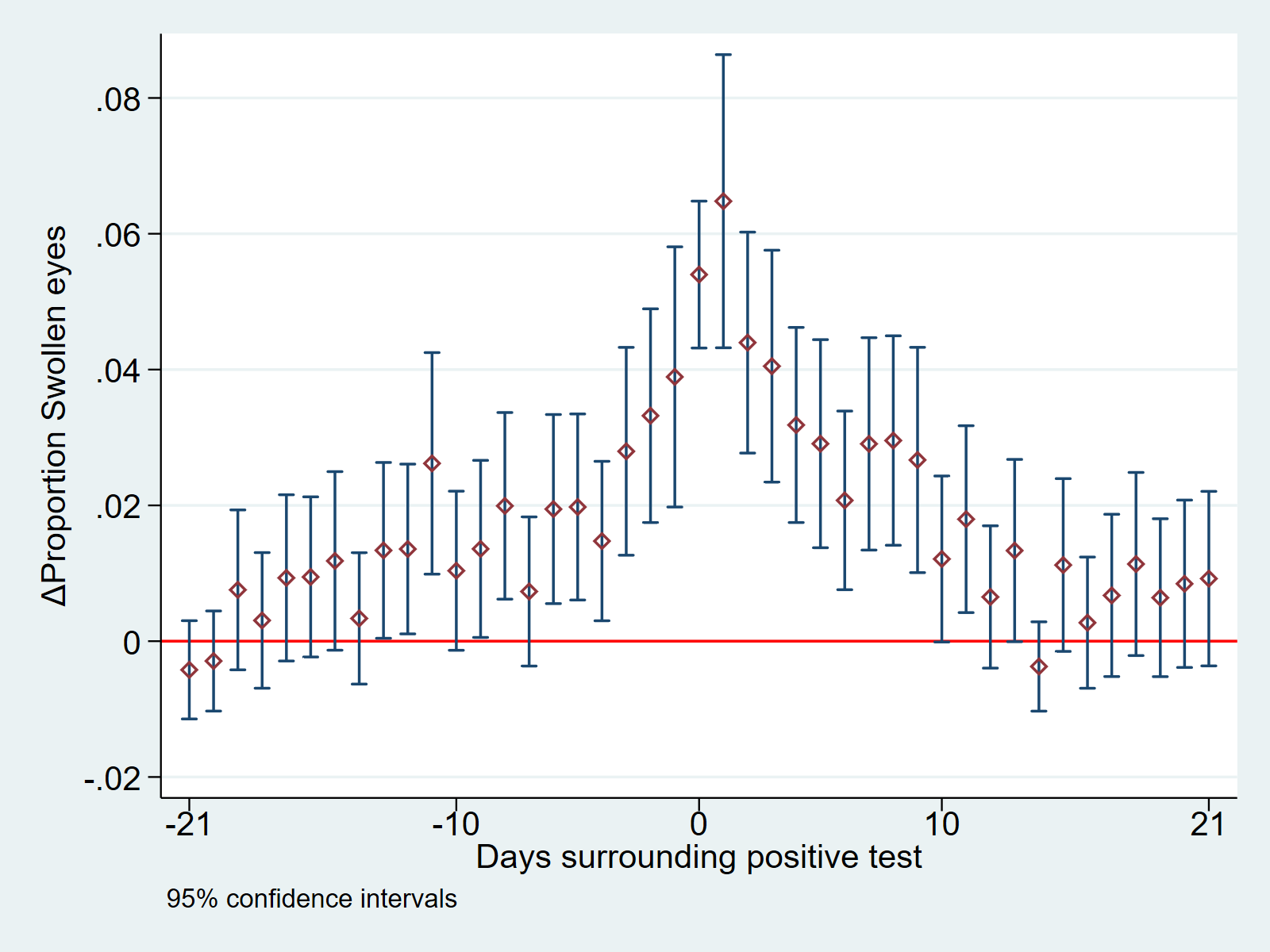

Supplement: S7 File — (ZIP) [file pone.0253566.s023.zip › sensitivity/noLoyalogen.tif]

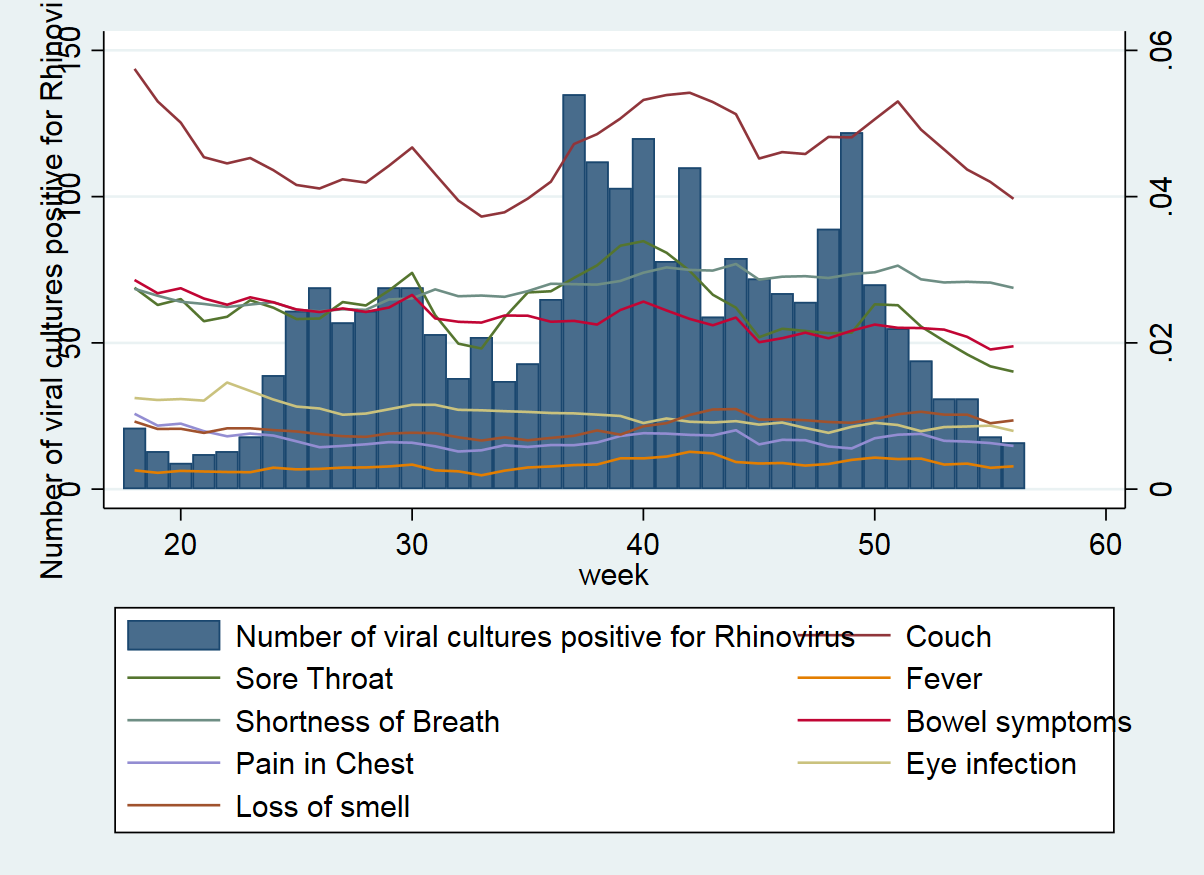

Supplement: S7 File — (ZIP) [file pone.0253566.s023.zip › sensitivity/noLoyalrhinovirus.tif]

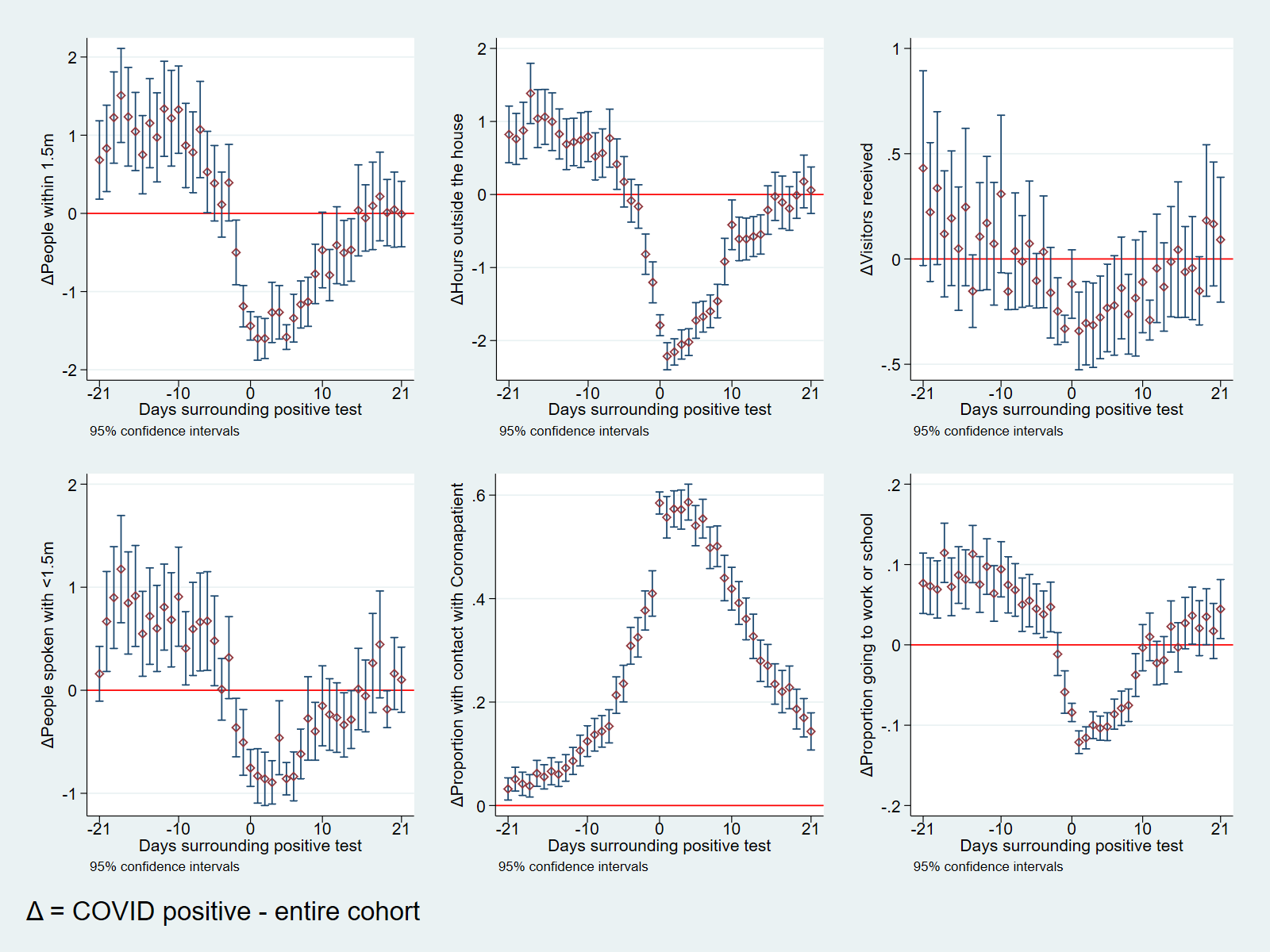

Supplement: S7 File — (ZIP) [file pone.0253566.s023.zip › sensitivity/noLoyalS19S25Behave.tif]

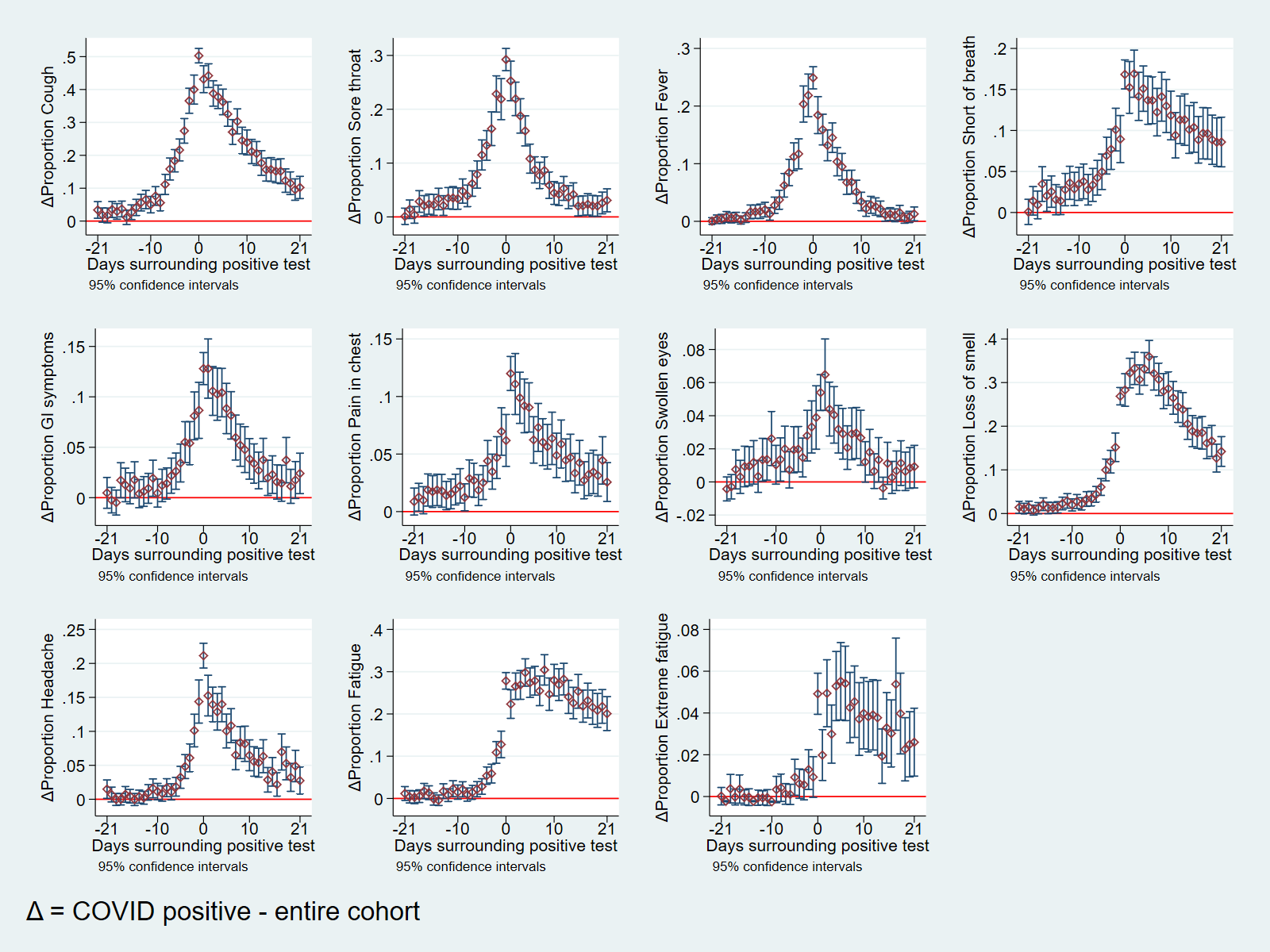

Supplement: S7 File — (ZIP) [file pone.0253566.s023.zip › sensitivity/noLoyalS6-S16AllSymp.tif]

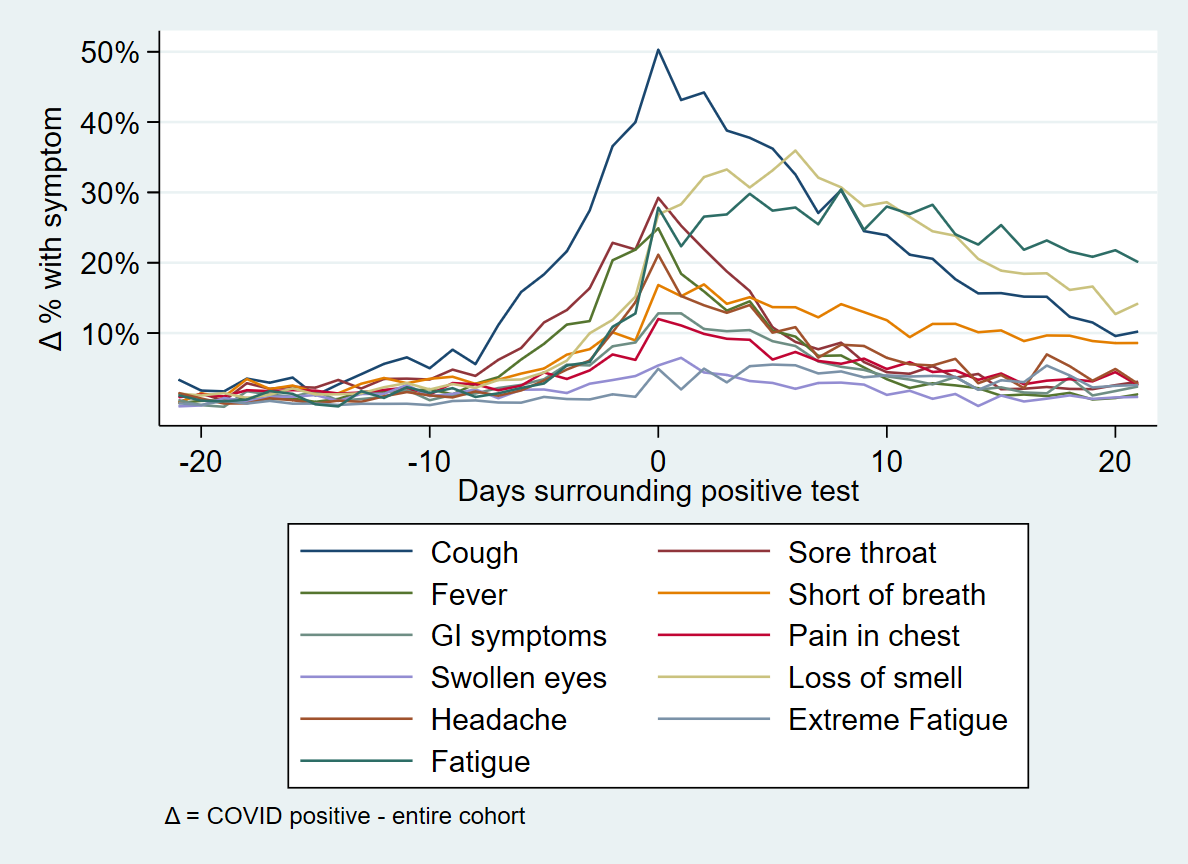

Supplement: S7 File — (ZIP) [file pone.0253566.s023.zip › sensitivity/noLoyalsymptomstest.tif]

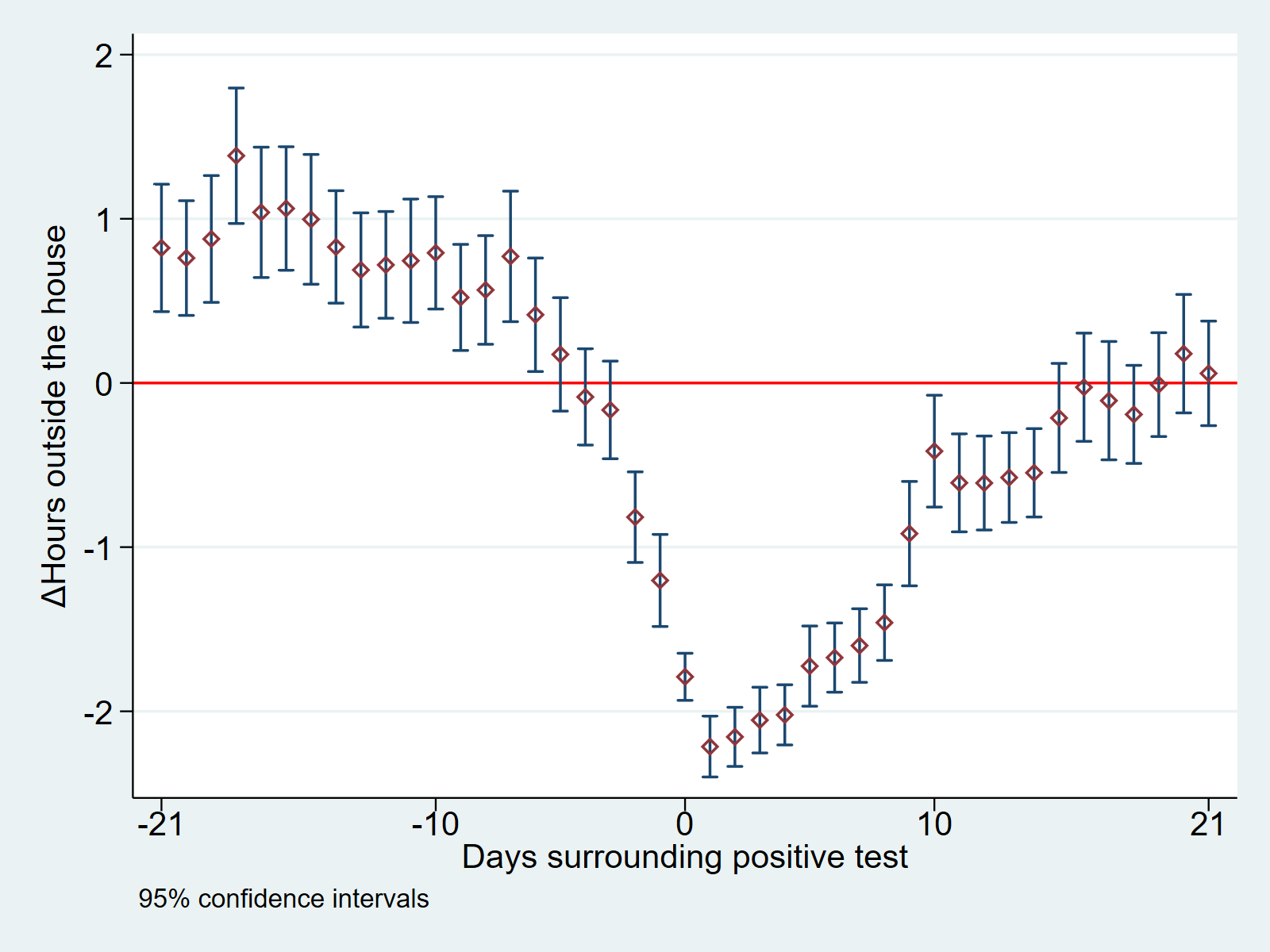

Supplement: S7 File — (ZIP) [file pone.0253566.s023.zip › sensitivity/noLoyaluithuis.tif]

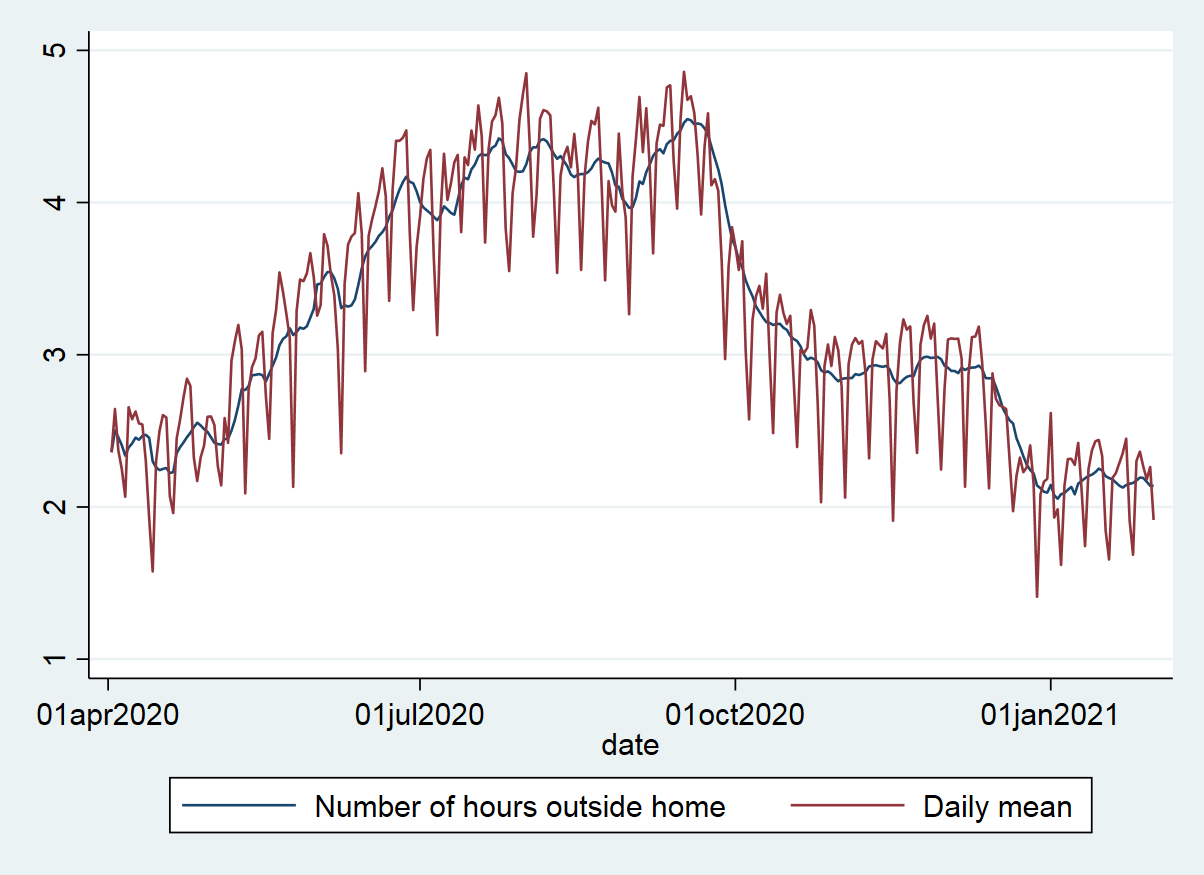

Supplement: S7 File — (ZIP) [file pone.0253566.s023.zip › sensitivity/noLoyalUithuistime.tif]

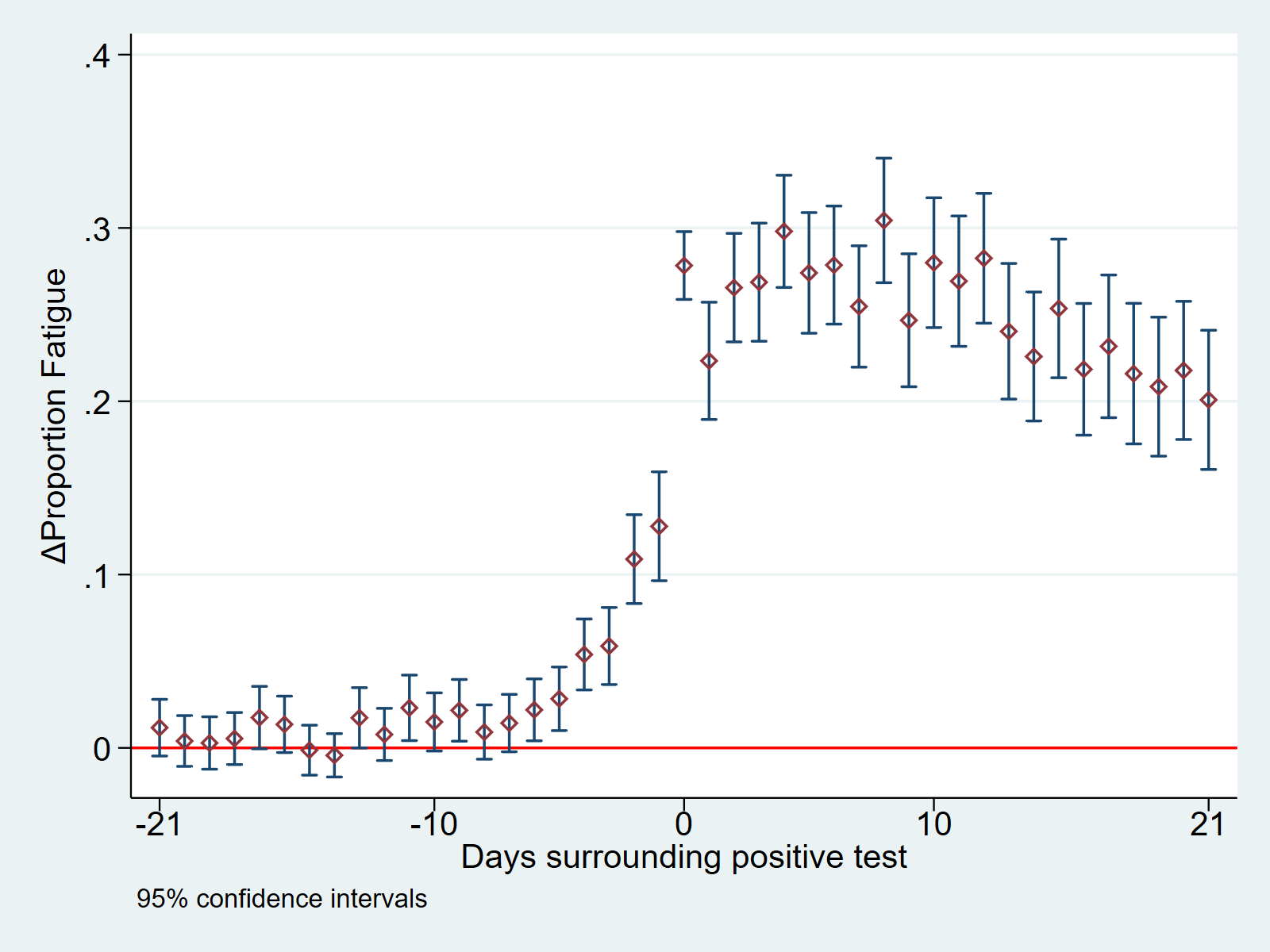

Supplement: S7 File — (ZIP) [file pone.0253566.s023.zip › sensitivity/noLoyalvermoeidheid.tif]

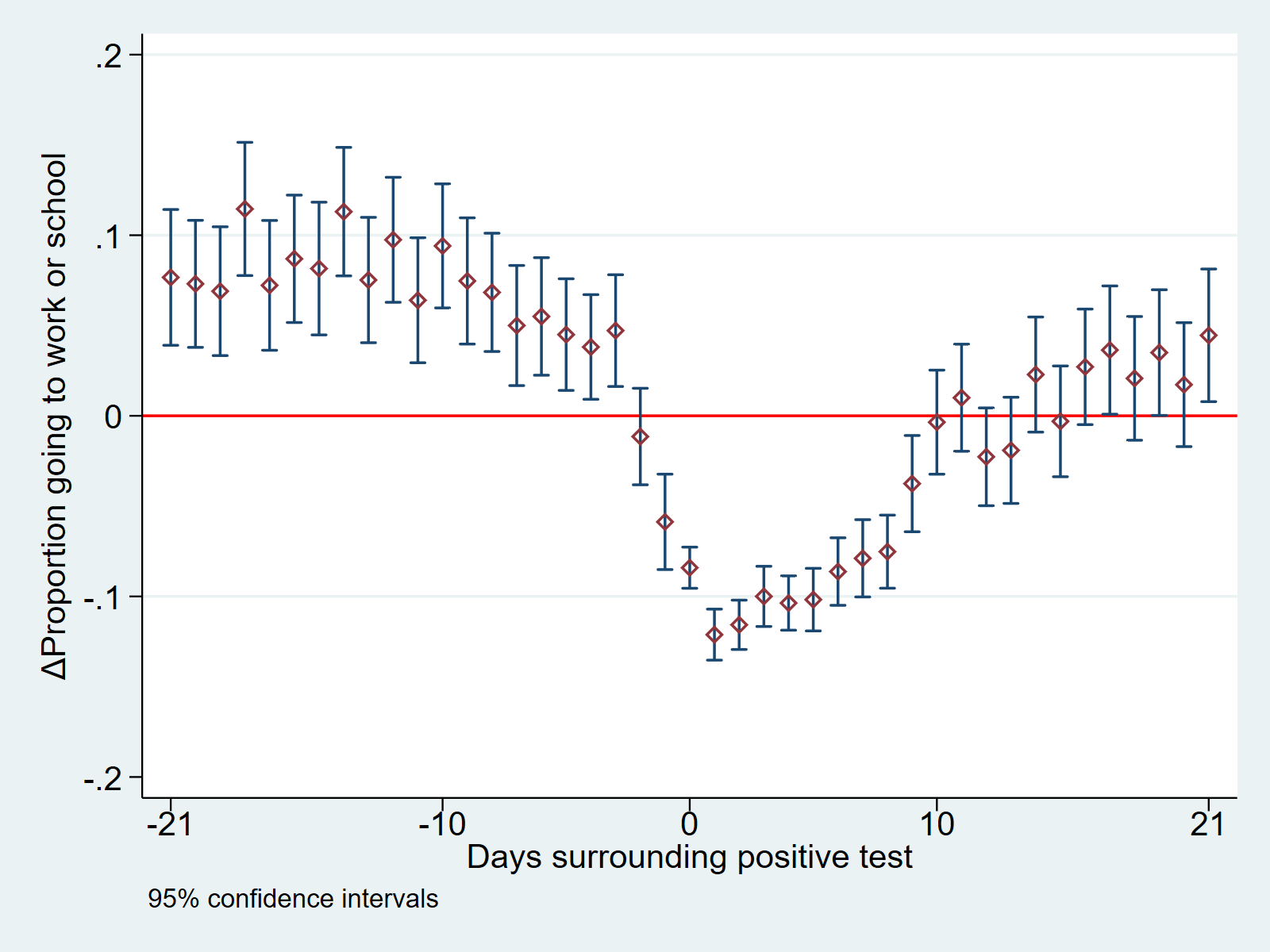

Supplement: S7 File — (ZIP) [file pone.0253566.s023.zip › sensitivity/noLoyalwerk.tif]

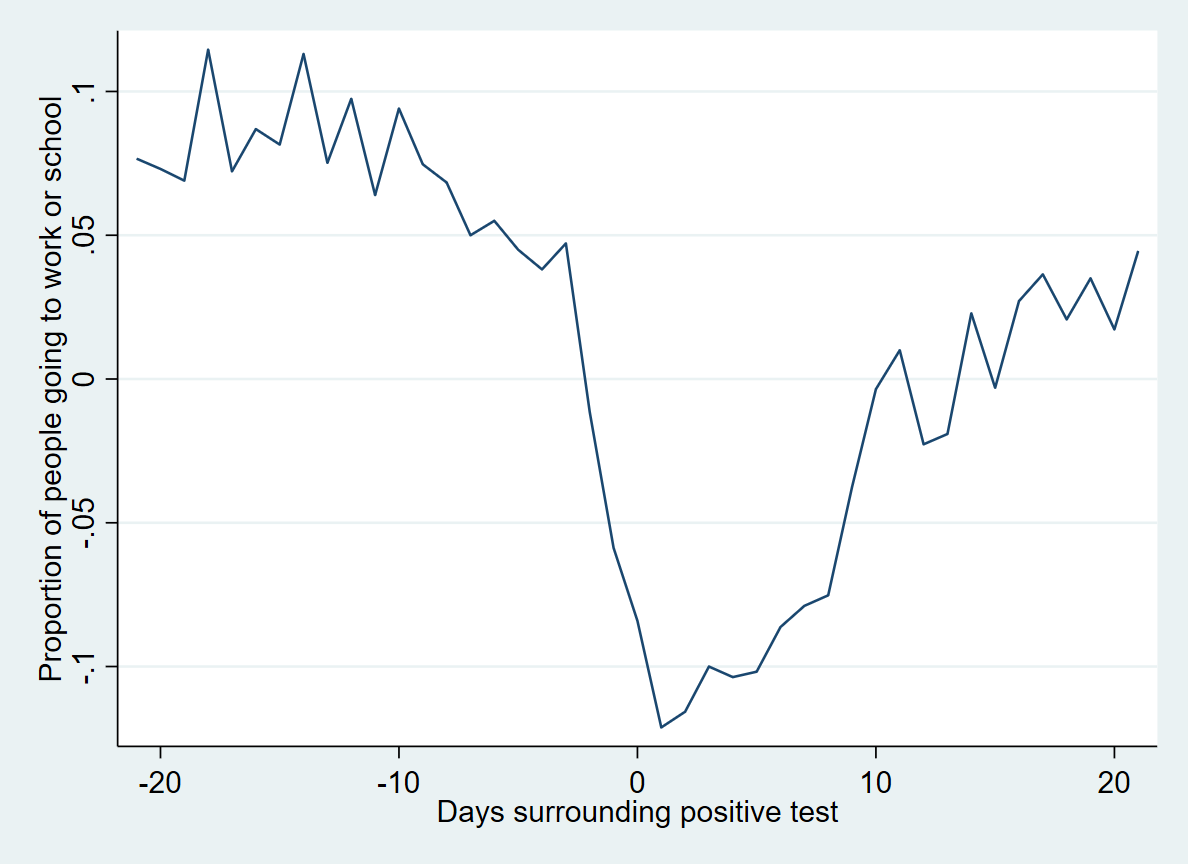

Supplement: S7 File — (ZIP) [file pone.0253566.s023.zip › sensitivity/noLoyalwerktest.tif]

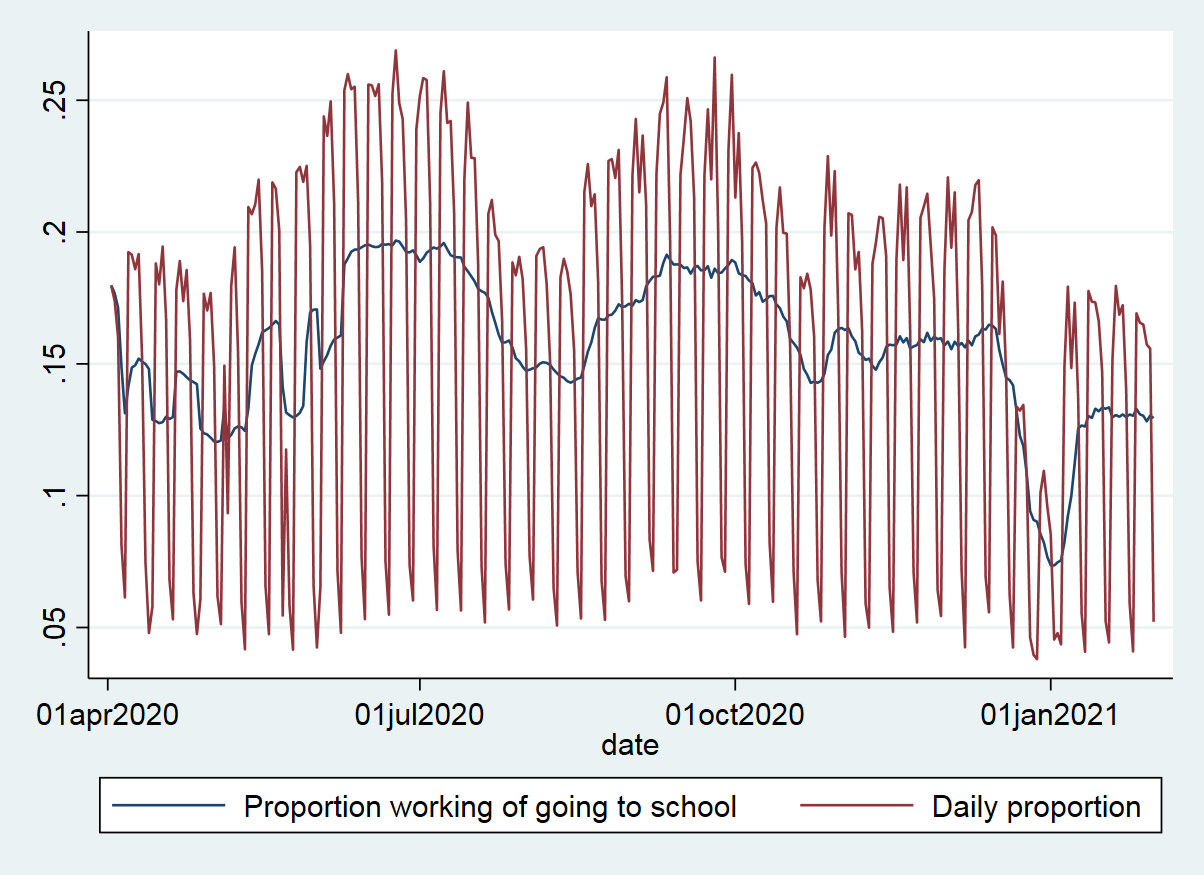

Supplement: S7 File — (ZIP) [file pone.0253566.s023.zip › sensitivity/noLoyalWerktime.tif]

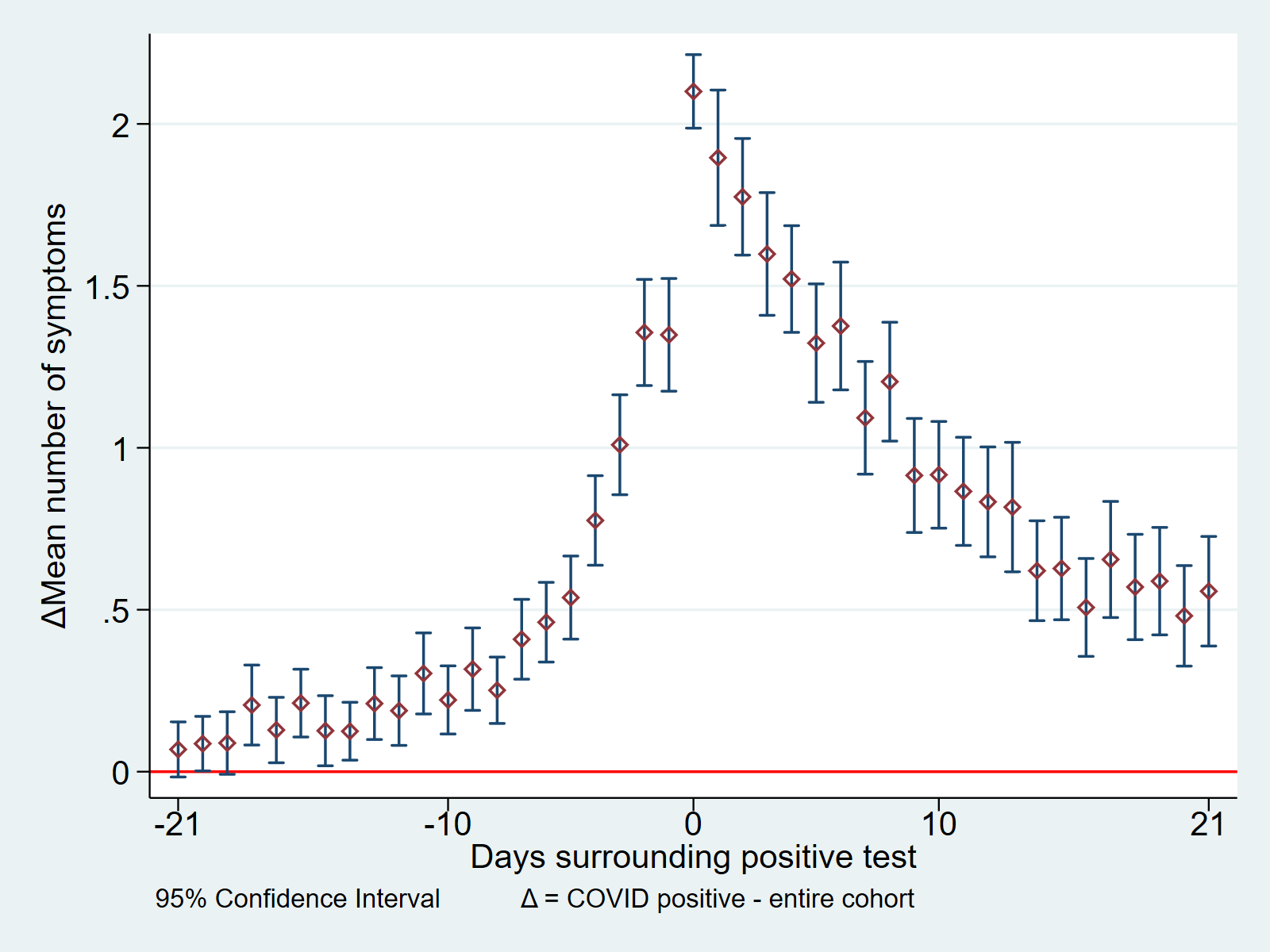

Supplement: S7 File — (ZIP) [file pone.0253566.s023.zip › sensitivity/noZHaantalsymp.tif]

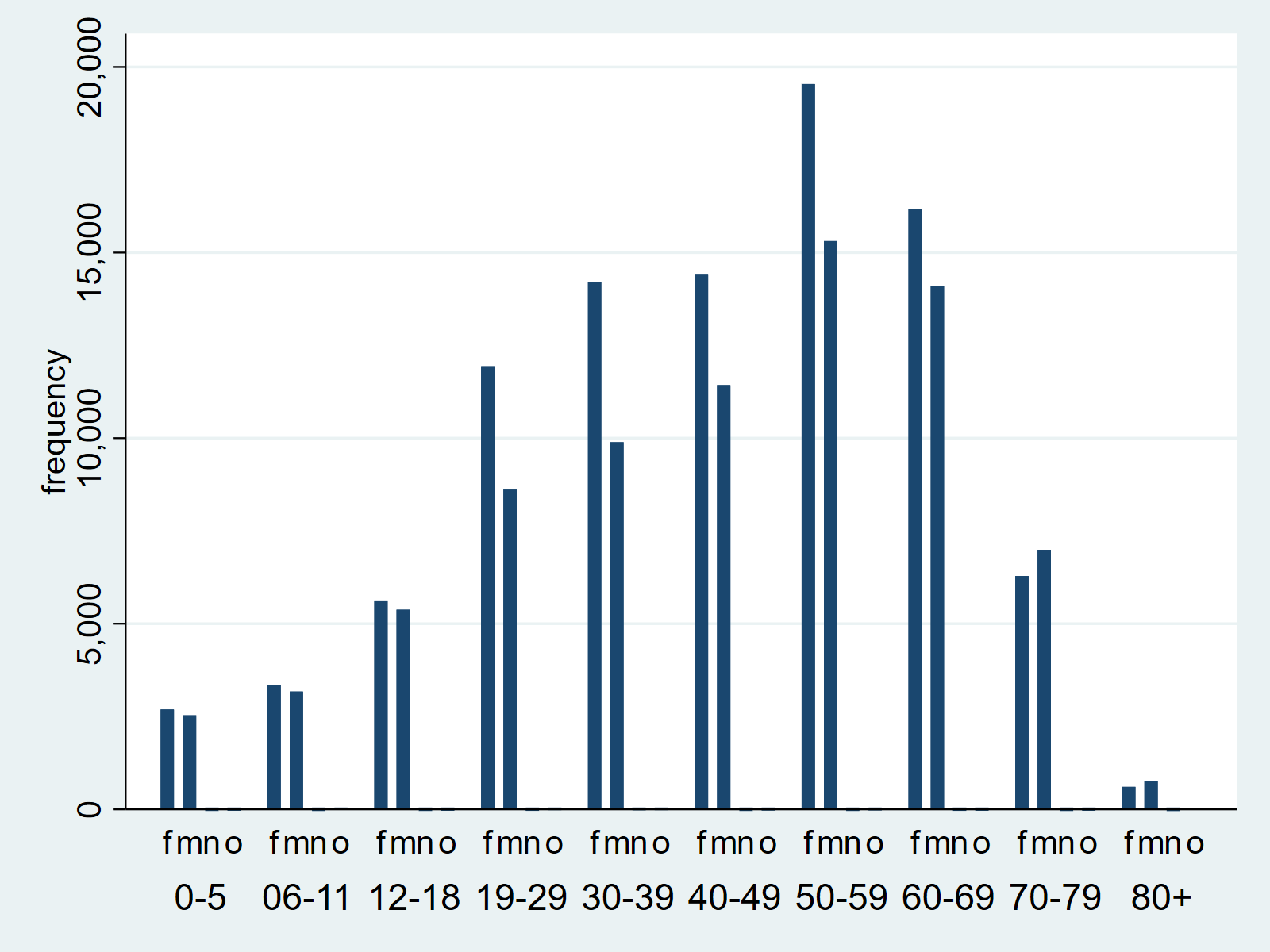

Supplement: S7 File — (ZIP) [file pone.0253566.s023.zip › sensitivity/noZHagegenderobs.tif]

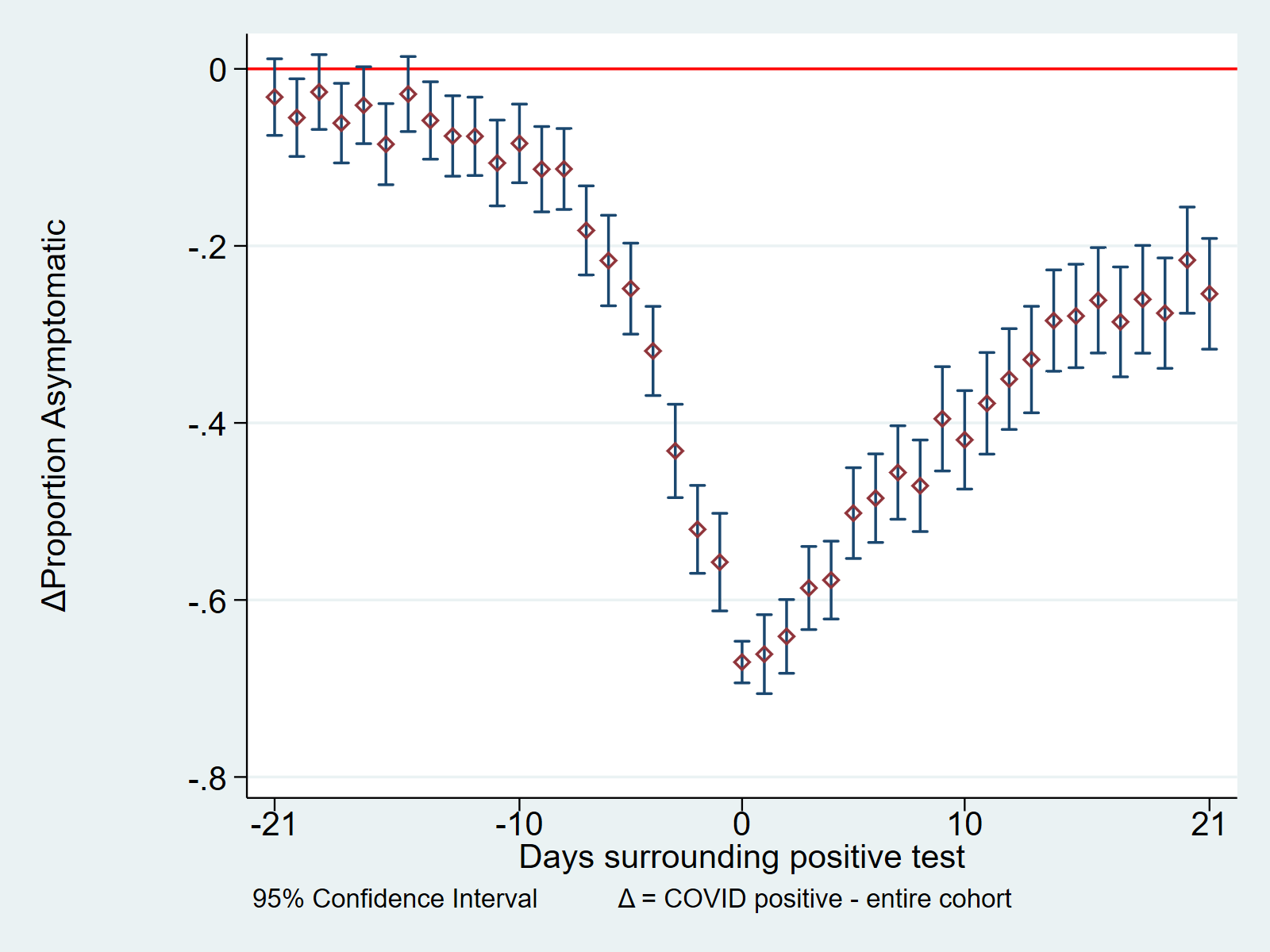

Supplement: S7 File — (ZIP) [file pone.0253566.s023.zip › sensitivity/noZHasymp.tif]

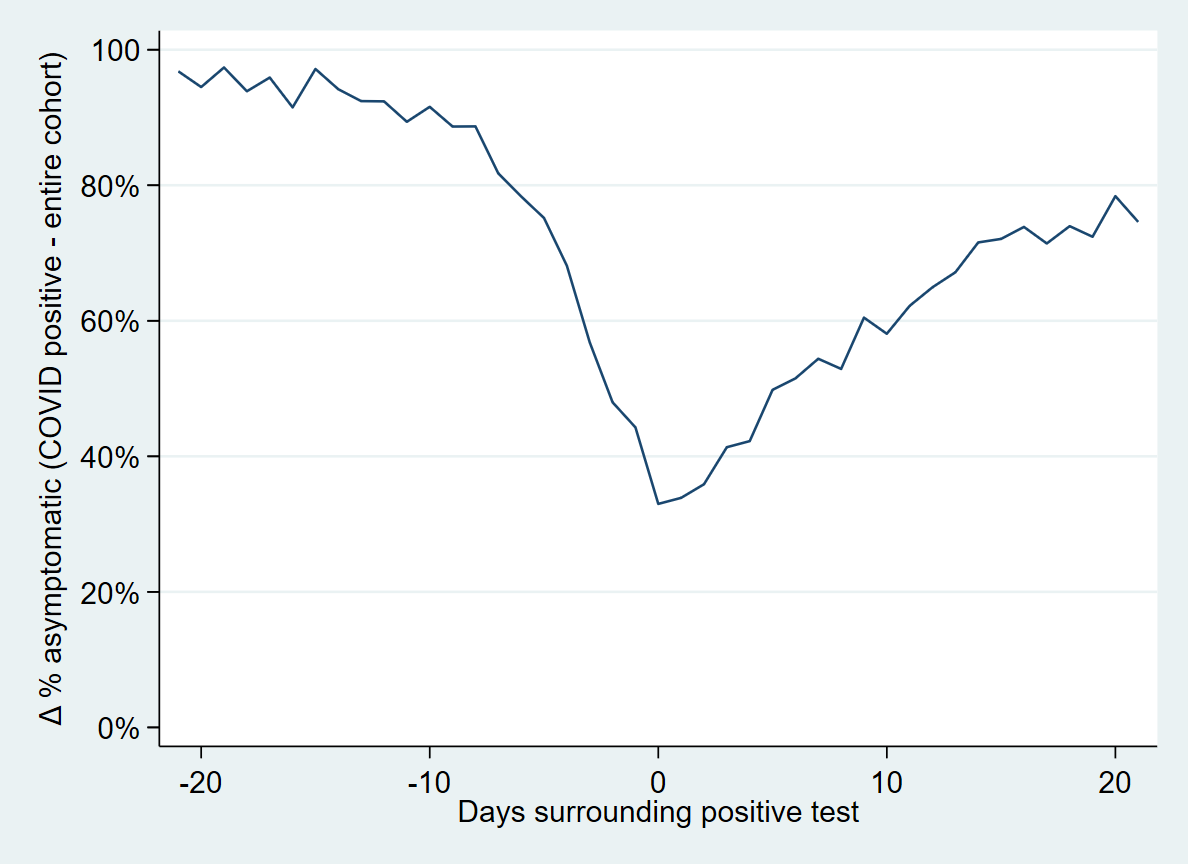

Supplement: S7 File — (ZIP) [file pone.0253566.s023.zip › sensitivity/noZHasymptest.tif]

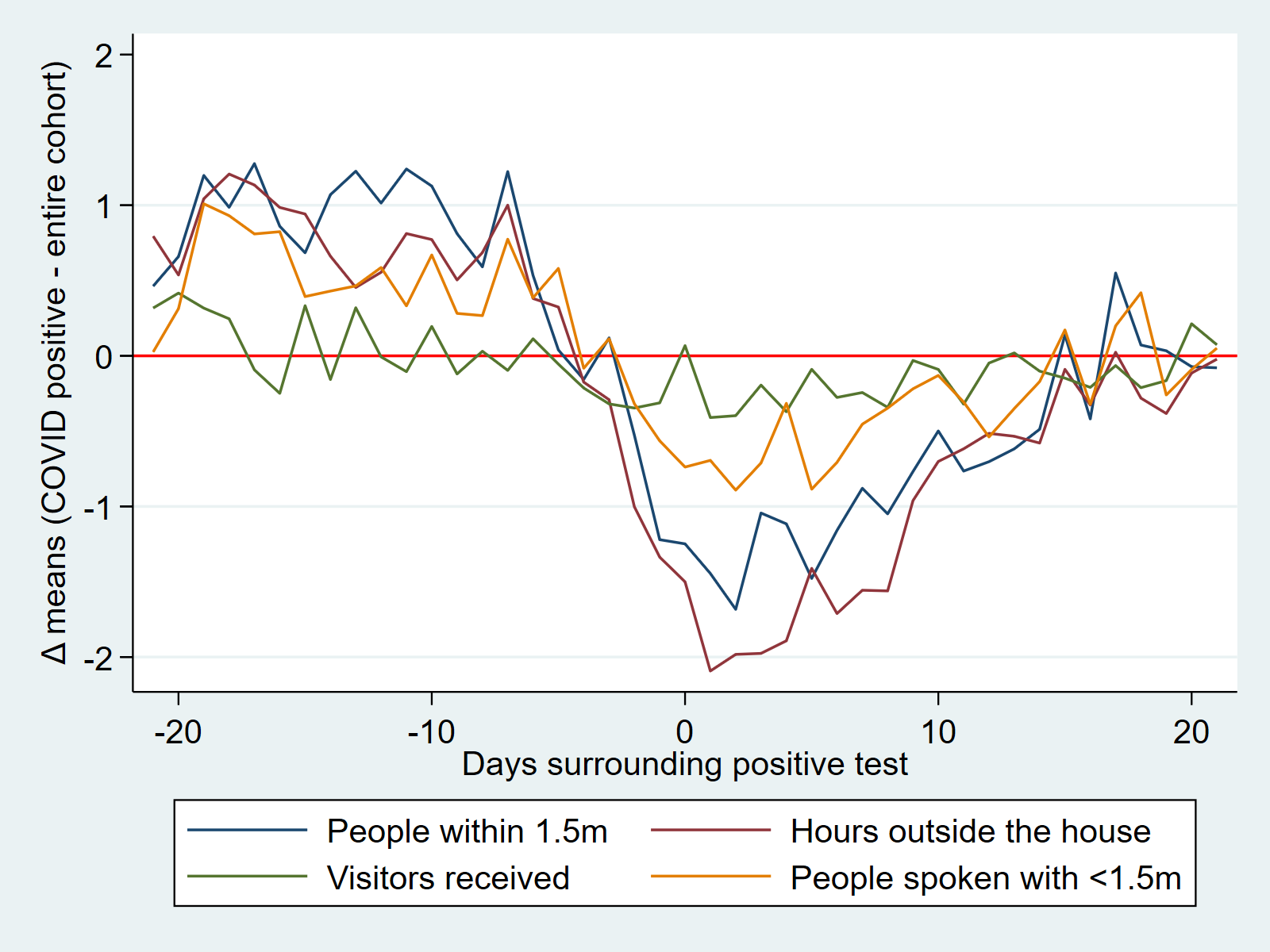

Supplement: S7 File — (ZIP) [file pone.0253566.s023.zip › sensitivity/noZHbehavetest.tif]

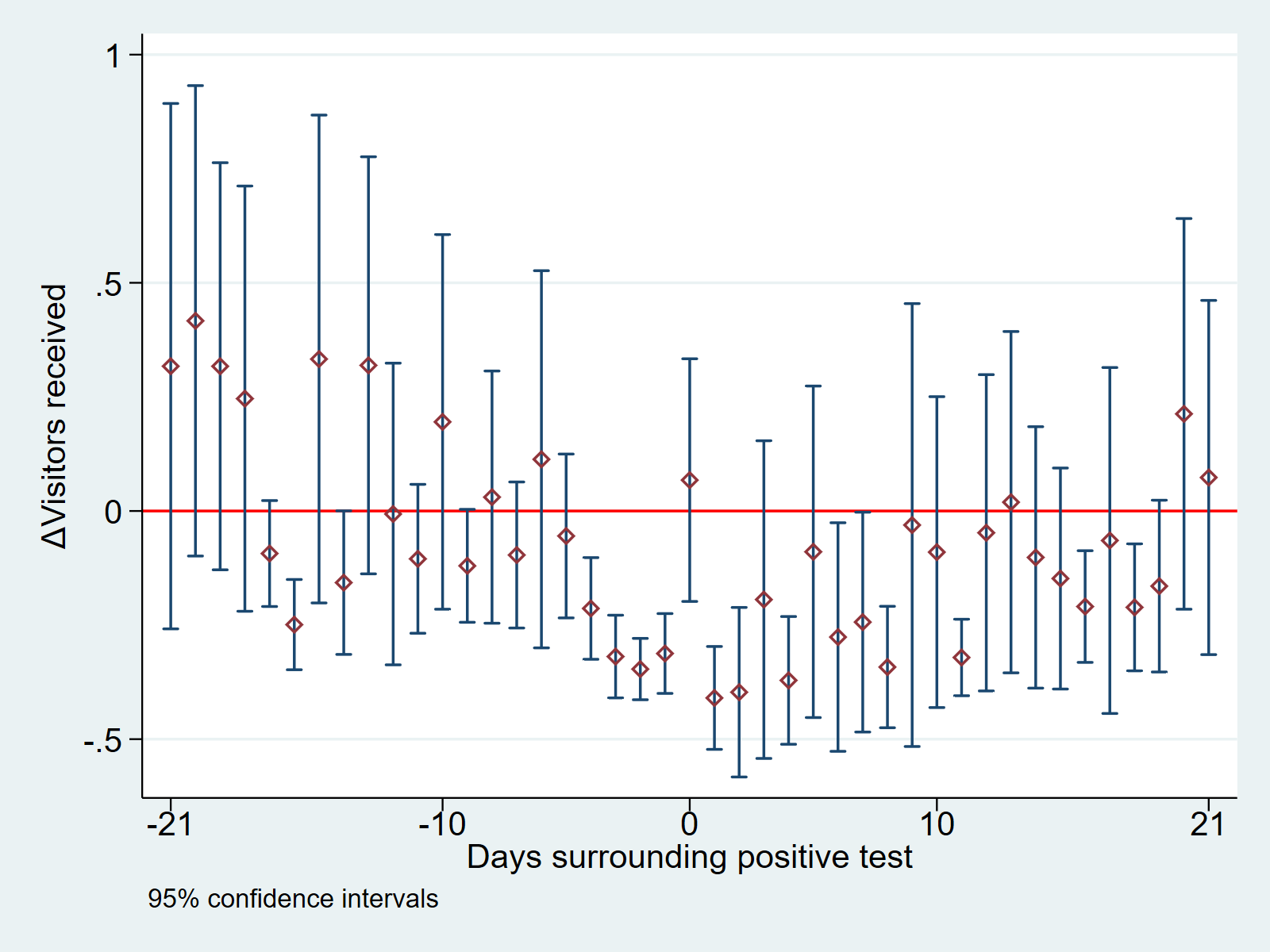

Supplement: S7 File — (ZIP) [file pone.0253566.s023.zip › sensitivity/noZHbezoek.tif]

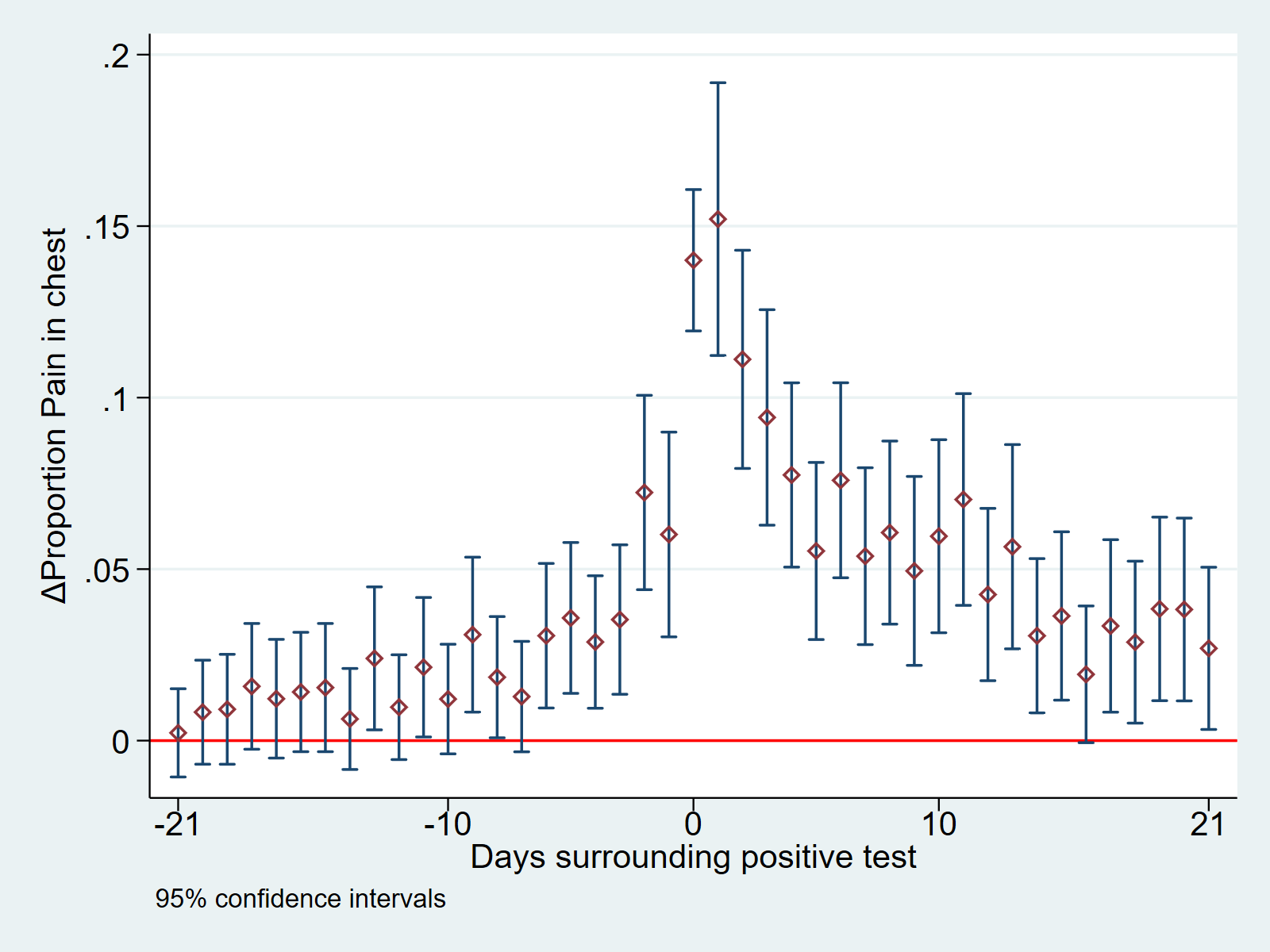

Supplement: S7 File — (ZIP) [file pone.0253566.s023.zip › sensitivity/noZHborstkas.tif]

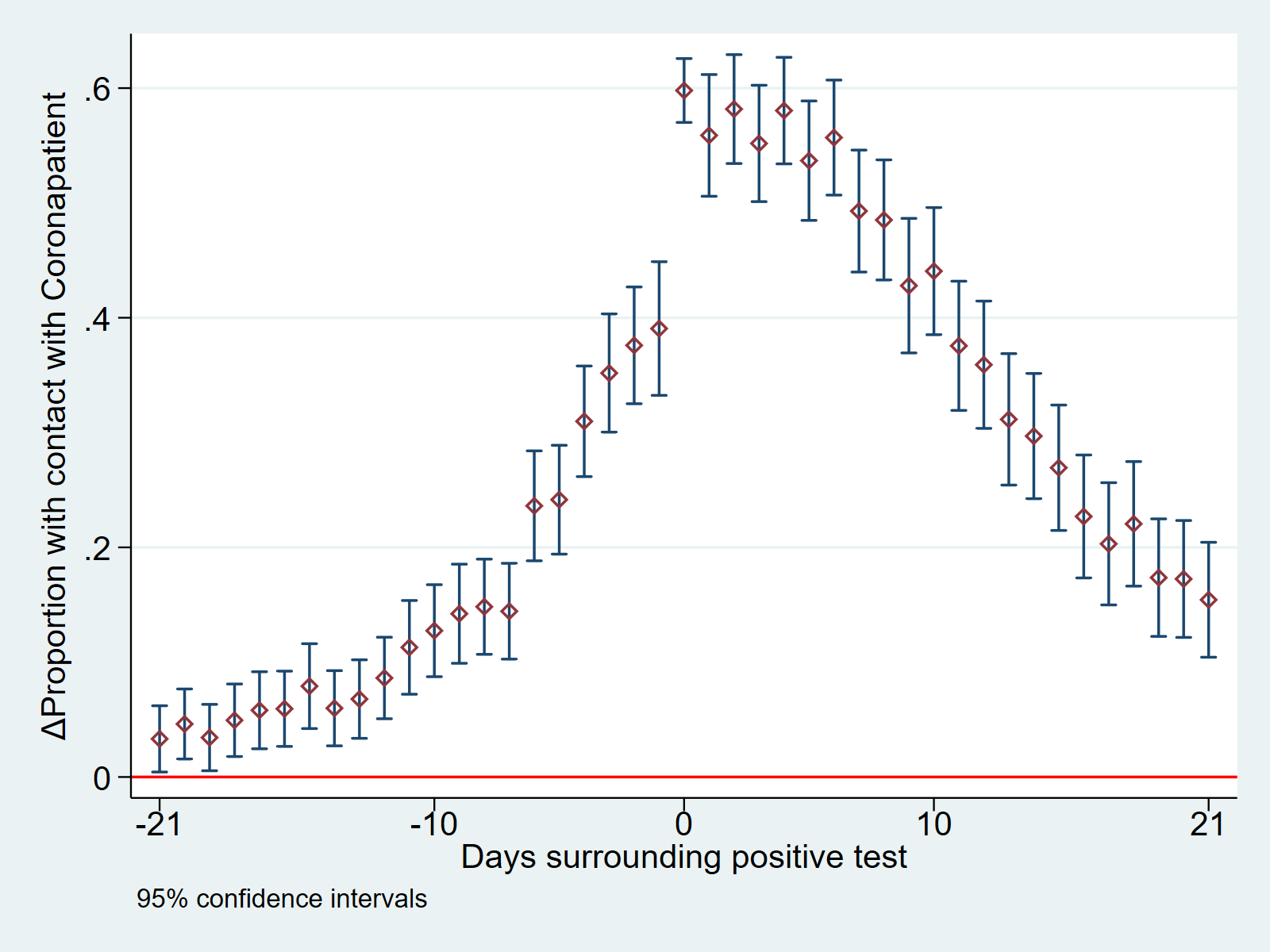

Supplement: S7 File — (ZIP) [file pone.0253566.s023.zip › sensitivity/noZHcontact.tif]

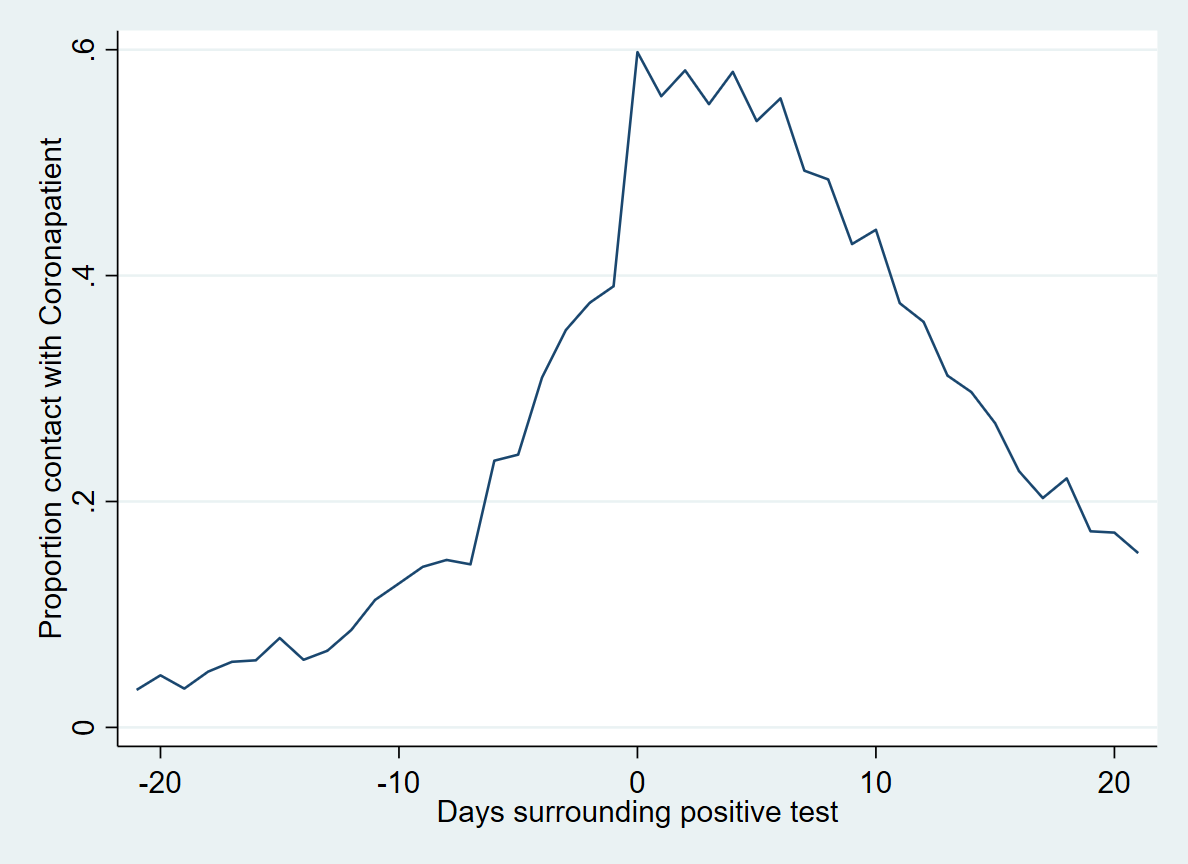

Supplement: S7 File — (ZIP) [file pone.0253566.s023.zip › sensitivity/noZHcontacttest.tif]

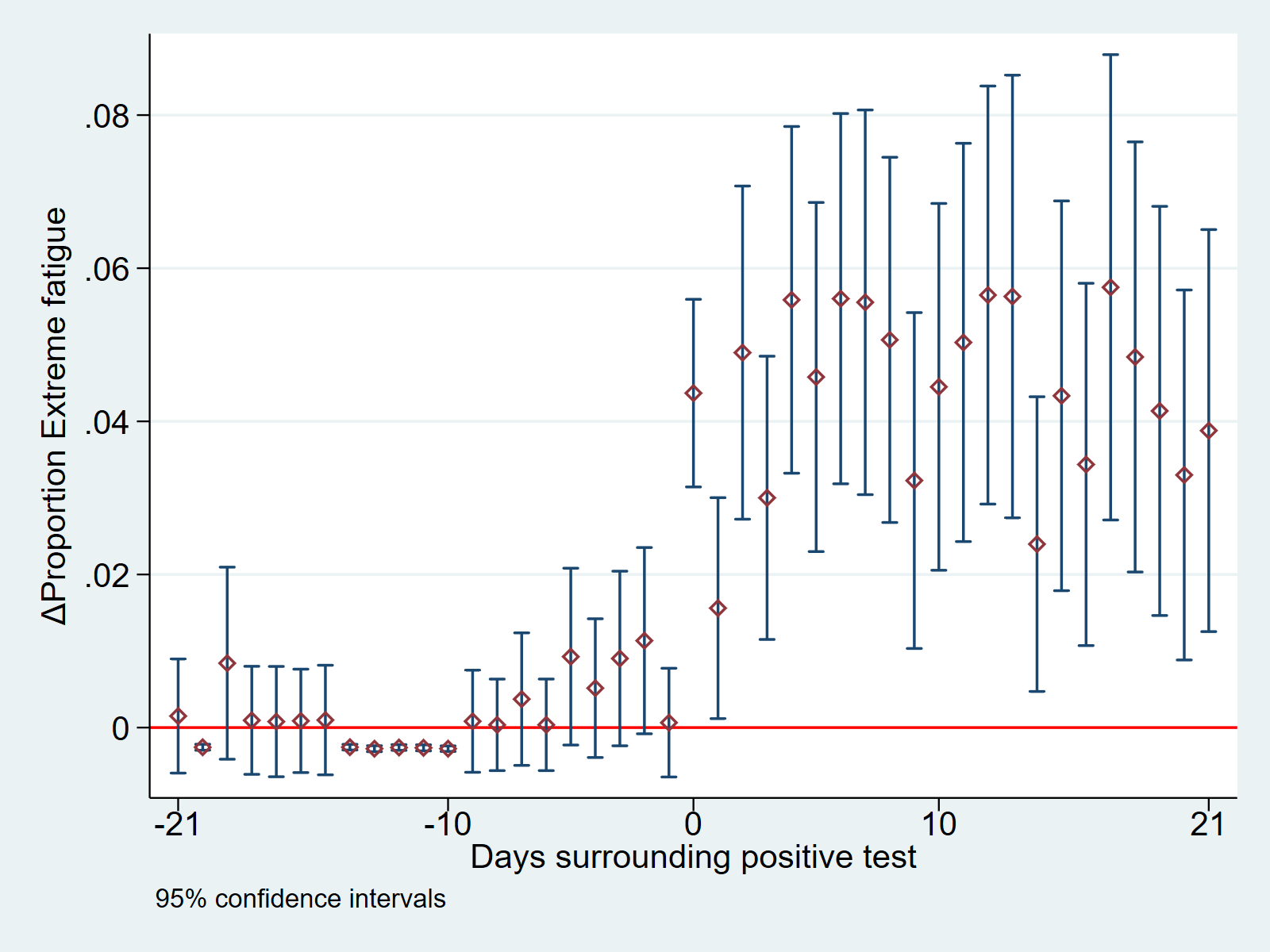

Supplement: S7 File — (ZIP) [file pone.0253566.s023.zip › sensitivity/noZHextreemvermoeidheid.tif]

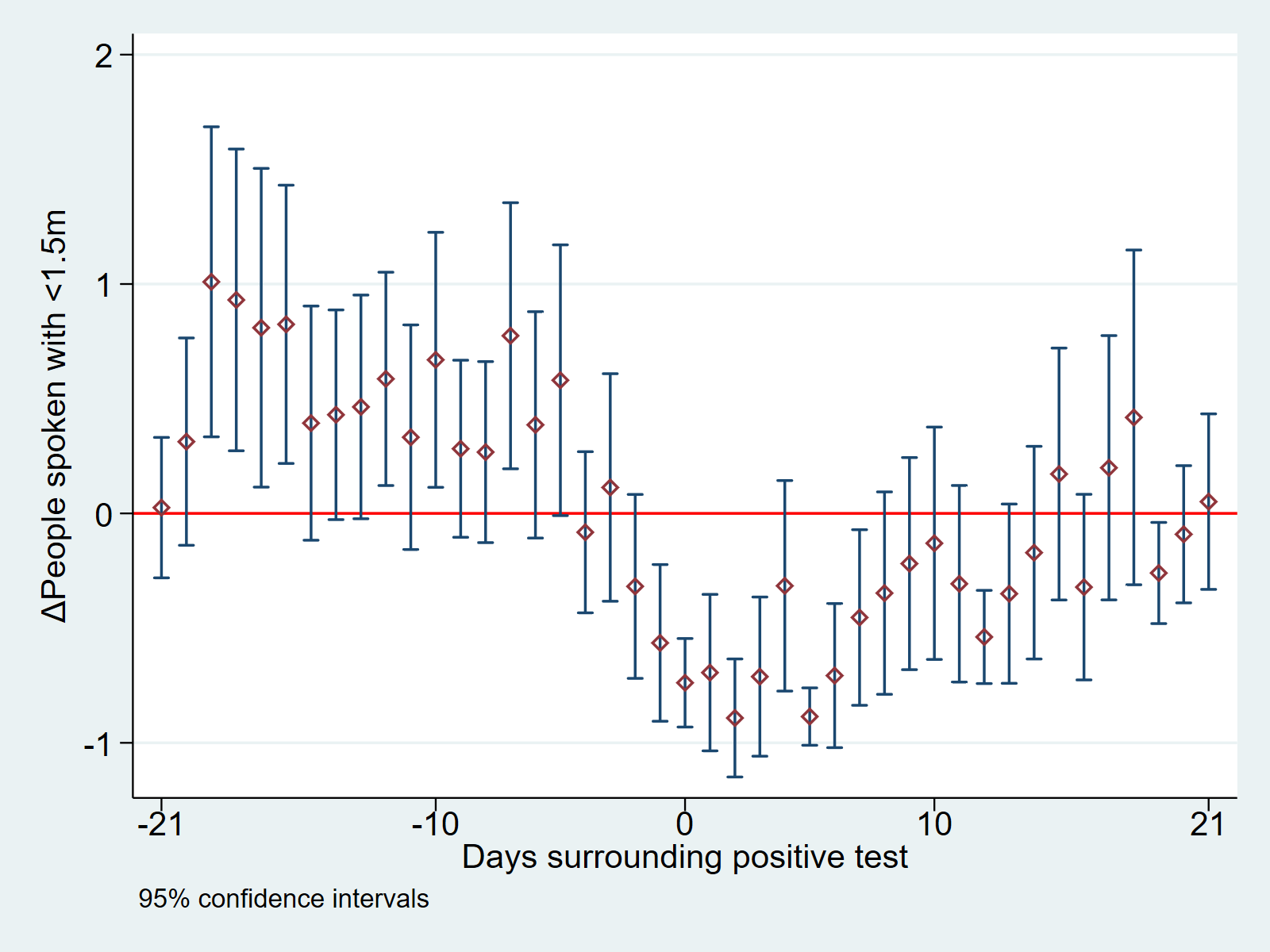

Supplement: S7 File — (ZIP) [file pone.0253566.s023.zip › sensitivity/noZHgesproken.tif]

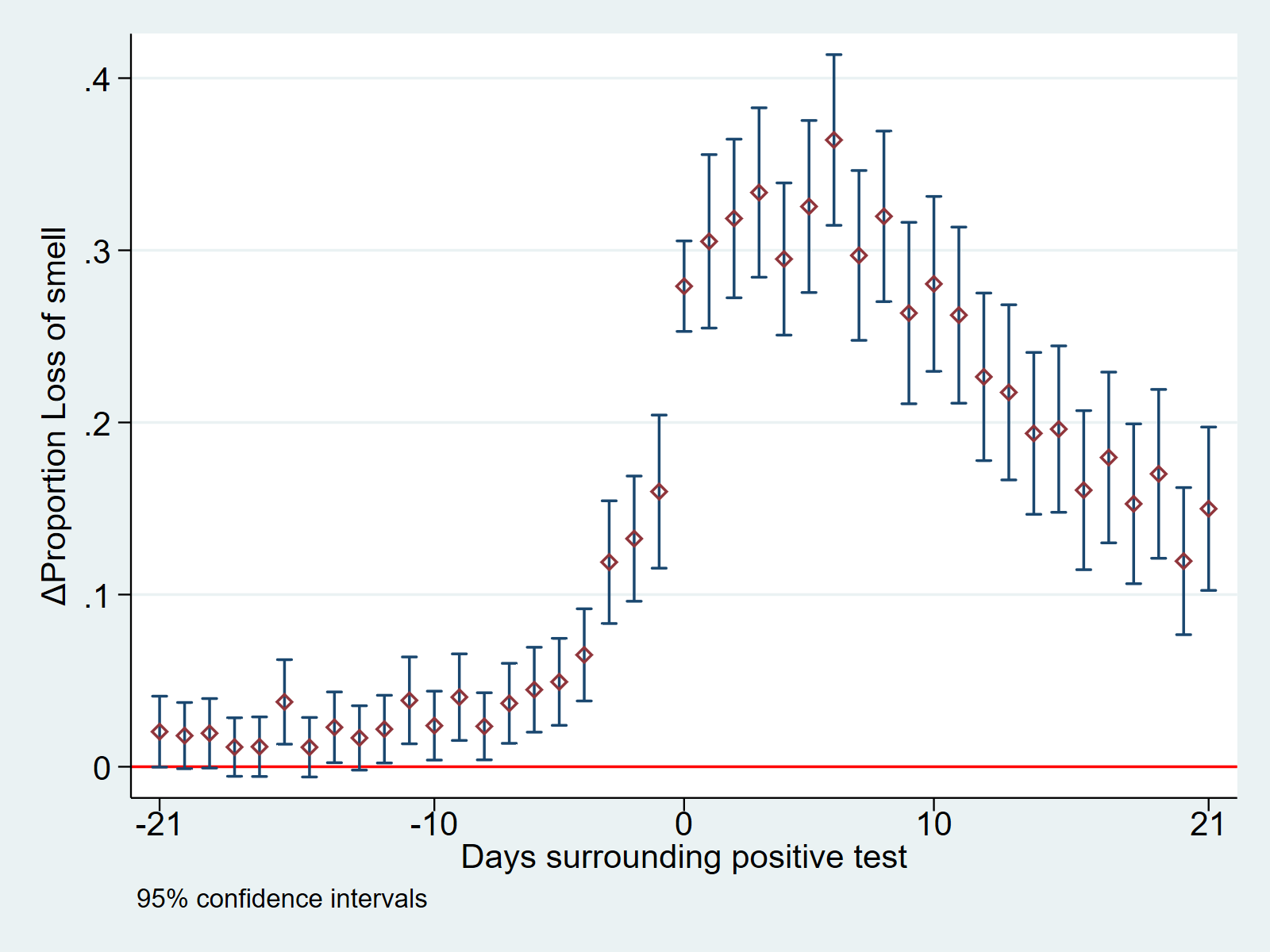

Supplement: S7 File — (ZIP) [file pone.0253566.s023.zip › sensitivity/noZHgeur.tif]

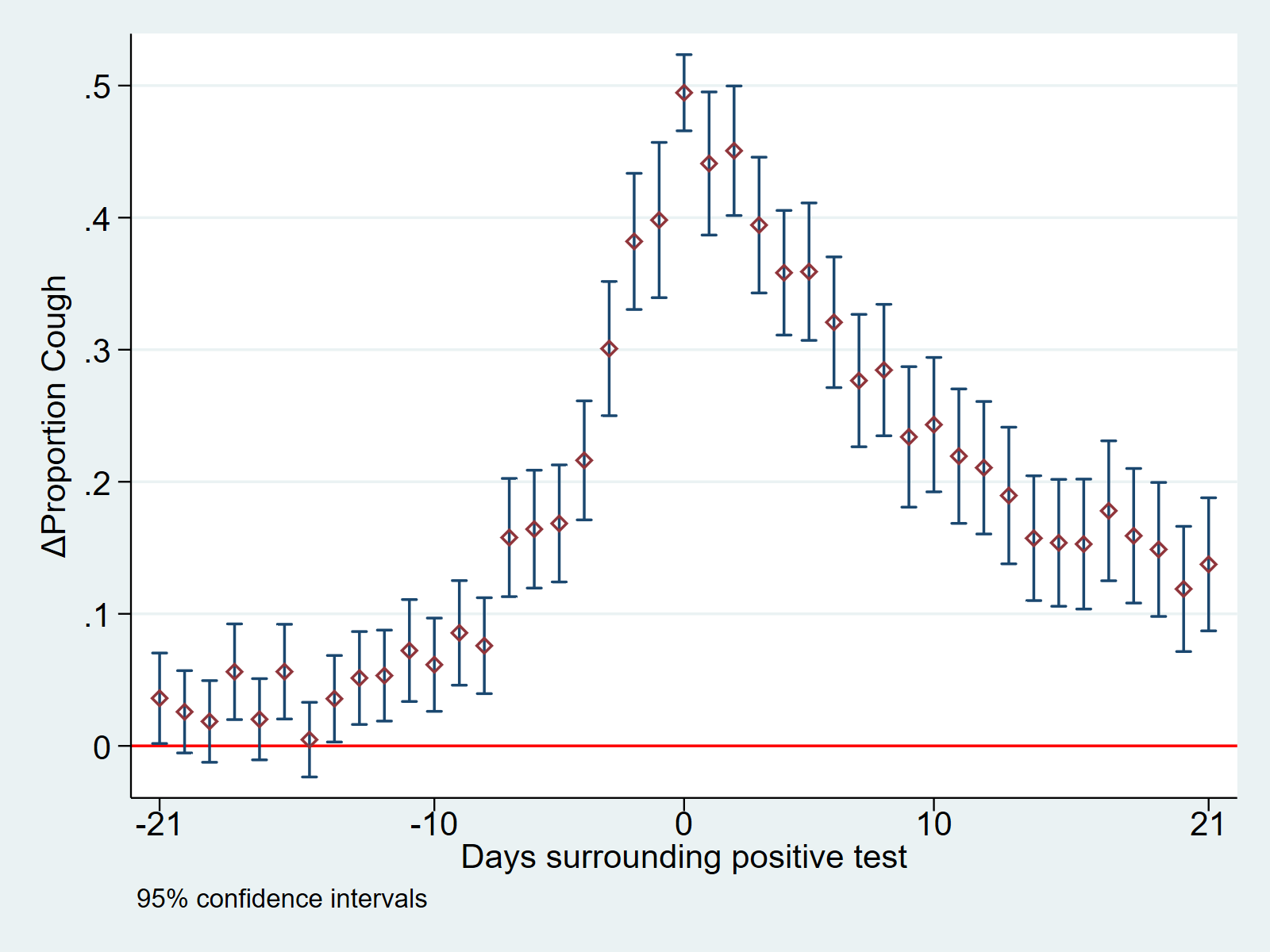

Supplement: S7 File — (ZIP) [file pone.0253566.s023.zip › sensitivity/noZHhoesten.tif]
