# Supplementary material for: COVID RADAR app: Description and validation of population surveillance of symptoms and behavior in relation to COVID-19
Source: PLoS One. 2021 Jun 30;16(6):e0253566. doi: 10.1371/journal.pone.0253566 (PMC8244909; doi:10.1371/journal.pone.0253566)
Supplement: S6 File — (DOCX) [file pone.0253566.s022.docx]

**Overview of development of the app**

*Design of the COVID Radar*

During the design phase of the app, an expert panel was commissioned with members from disciplines such as infectious diseases, clinical medicine, primary care, psychology, epidemiology, data-science and engineering. The questions forming the questionnaire were chosen based on expert opinion and a review of the peer-reviewed knowledge base for studies exploring COVID-19-related symptoms and behavior [1-4]. The app had been updated multiple times since its release in April 2020, with the questionnaire adjusted based on the advice of the expert group and user feedback. List of updates:

- May: Addition of reminders
- May: Addition of update notification
- May: broadening of answer options of nearby contacts from 0-20 to 0-50
- July: Change of contacts within 5 meters answer option from integer to discrete.
- July: Addition of question “Gesproken” (how many people have you spoken with within 1.5 meters”)
- August: Addition of questions: Sport (“Did you sport yesterday?”) and Vacation (Are you on holiday?”)
- September: Addition of variable profession
- October: Addition of variable Fatique and Headache
- December: Addition of registrations of Negative tests
- December: Addition of questions about Masks and Public places

To maximize user adherence, we added persuasive system design elements to the COVID Radar, such as reminders, personal feedback, and social comparison [3].

*Data Sources and cleaning*

Data were collected on an ORTEC server and transferred daily to a safe data environment within the Information Technology system of the LUMC (supplemental Figure 2). Following importation of the daily data, we exclude observations from users who had requested to opt out, observations listing nonexistent postcodes, and double measurements within one user. For users with multiple measurements within one day, we included the observation with the fewest missing values. We considered users COVID-19 positive/negative if they indicated a COVID-19 test result at least twice in the app, with the date of the first report used as day zero. This was to reduce the amount of (we assume) false indicated tests results (if positive once in the app, the default answer should stay on ‘positive test result’).

For comparison of COVID Radar user-reported positive COVID-19 tests with those reported nationally, we used the daily COVID-19 case count per municipality (355 in the Netherlands with a median of ~31,000 inhabitants) as reported by the National Institute for Public Health and the Environment (Rijksinstituut voor Volksgezondheid en Milieu - RIVM) which itself collects these figures from the nation’s 26 Municipal Health Services (GGD) [5]. We excluded data which was not region specific. These data are linked with the weekly reported number of positive viral cultures reported by 21 Dutch laboratories serving as the country’s testing apparatus for viral surveillance [6]. We extracted the cultures positive for Rhinovirus given this virus’s symptoms most resemble those of COVID-19. Demographic data about postal code inhabitants are as reported by the Dutch Central Bureau of Statistics (CBS) [7].

Users can adjust their personal information at will (e.g. changing postal code while on vacation). The four digits of the Dutch postal code specify the region of each user, with regions comprising hundreds to tens of thousands of inhabitants. This allows placing a user’s location without identifying the user. Users are asked to answer about symptoms on that particular day, and their behaviors on the day before. A push notification reminder is sent to all users every other day to remind and encourage users to fill out the questionnaire each day.

For missing values in the questions “have you been to work/school yesterday”, “number of hours outside of house” and “number of persons within 5 meters” the number 0 was used. For unreal high numbers the maximum number of 50 was used.

1. Drew DA, Nguyen LH, Steves CJ, Menni C, Freydin M, Varsavsky T, et al. Rapid implementation of mobile technology for real-time epidemiology of COVID-19. Science. 2020;368(6497):1362-7. doi: 10.1126/science.abc0473.

2. Menni C, Valdes AM, Freidin MB, Sudre CH, Nguyen LH, Drew DA, et al. Real-time tracking of self-reported symptoms to predict potential COVID-19. Nature Medicine. 2020;26(7):1037-40. doi: 10.1038/s41591-020-0916-2.

3. Oinas-Kukkonen H. Persuasive systems design: key issues, process model, and system features. Communication Association Information System. 2009;24.

4. Kelders SM, Kok RN, Ossebaard HC, Van Gemert-Pijnen JE. Persuasive system design does matter: a systematic review of adherence to web-based interventions. J Med Internet Res. 2012;14(6):e152.

5. (RIVM) RvVeM. Actuele informatie over het nieuwe coronavirus COVID-19 2020 [cited 01-12-2020]. Available from: <https://data.rivm.nl/geonetwork/srv/dut/catalog.search#/search?resultType=details&any_OR__title=covid-19&isChild='false'&fast=index&sortBy=relevance&from=1&to=20>.

6. (RIVM) RvVeM. Virologische Weekstaten <https://www.rivm.nl/virologische-weekstaten2021> [cited 1-2-2021].

7. Statistics CBf. Neighborhood, district and municipality 2020 for postal code and house number [Dataset] <https://www.cbs.nl/nl-nl/maatwerk/2020/39/buurt-wijk-en-gemeente-2020-voor-postcode-huisnummer2020> [cited 2020 1-11-2020].
