# Supplementary figures and images for: COVID RADAR app: Description and validation of population surveillance of symptoms and behavior in relation to COVID-19 (part 2 of 2)
Source: PLoS One. 2021 Jun 30;16(6):e0253566. doi: 10.1371/journal.pone.0253566 (PMC8244909; doi:10.1371/journal.pone.0253566)

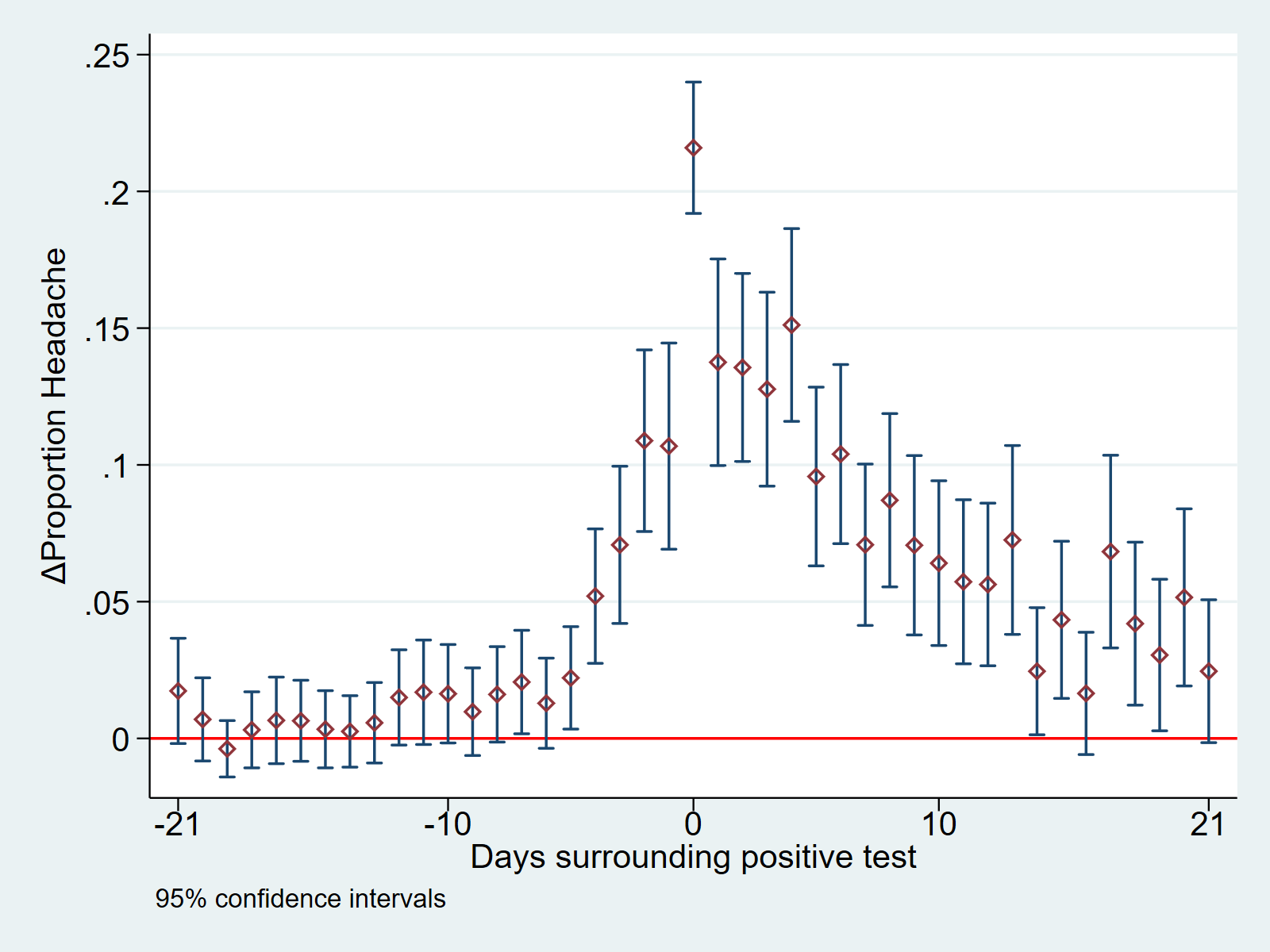

Supplement: S7 File — (ZIP) [file pone.0253566.s023.zip › sensitivity/noZHhoofdpijn.tif]

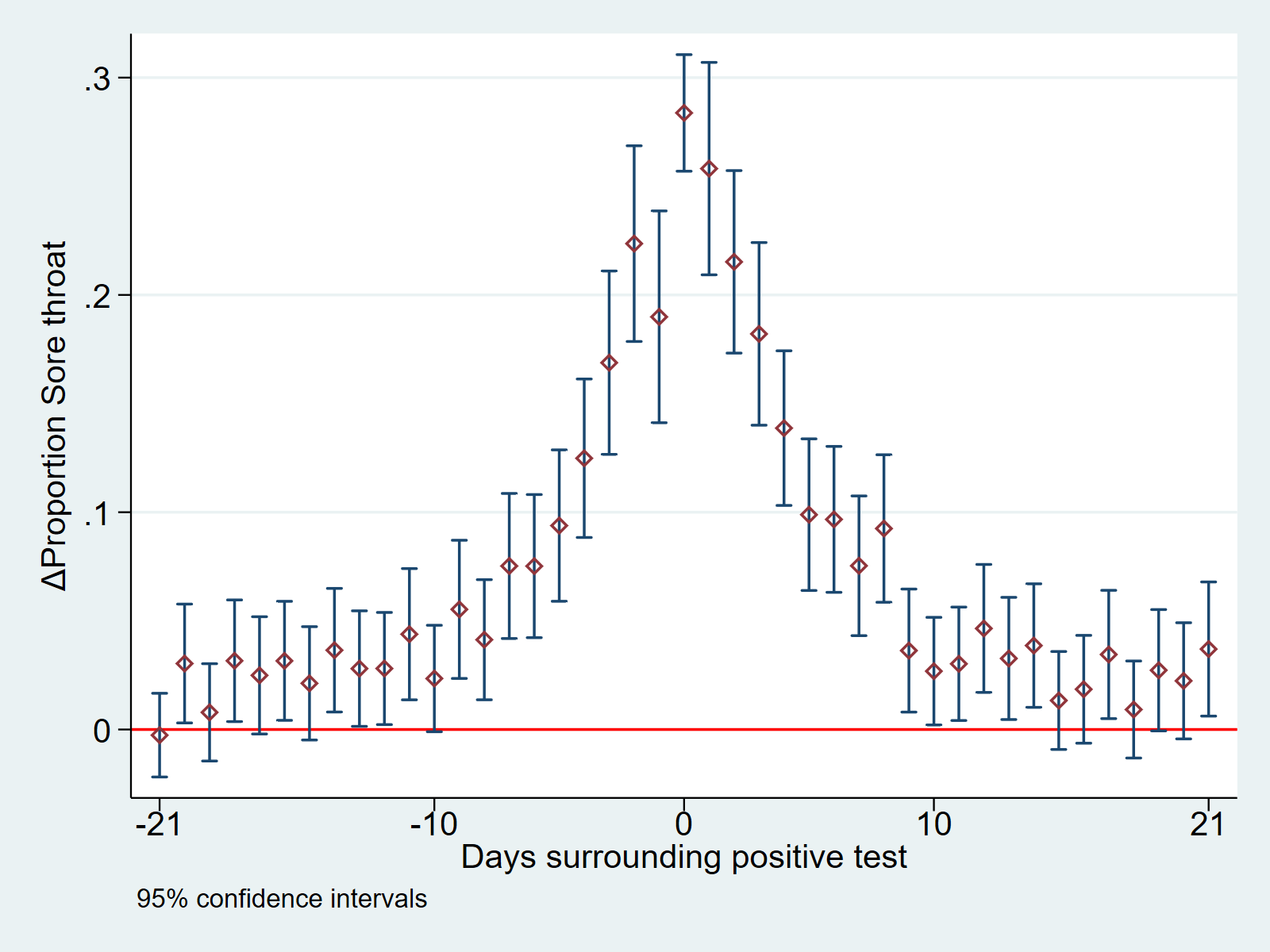

Supplement: S7 File — (ZIP) [file pone.0253566.s023.zip › sensitivity/noZHkeelpijn.tif]

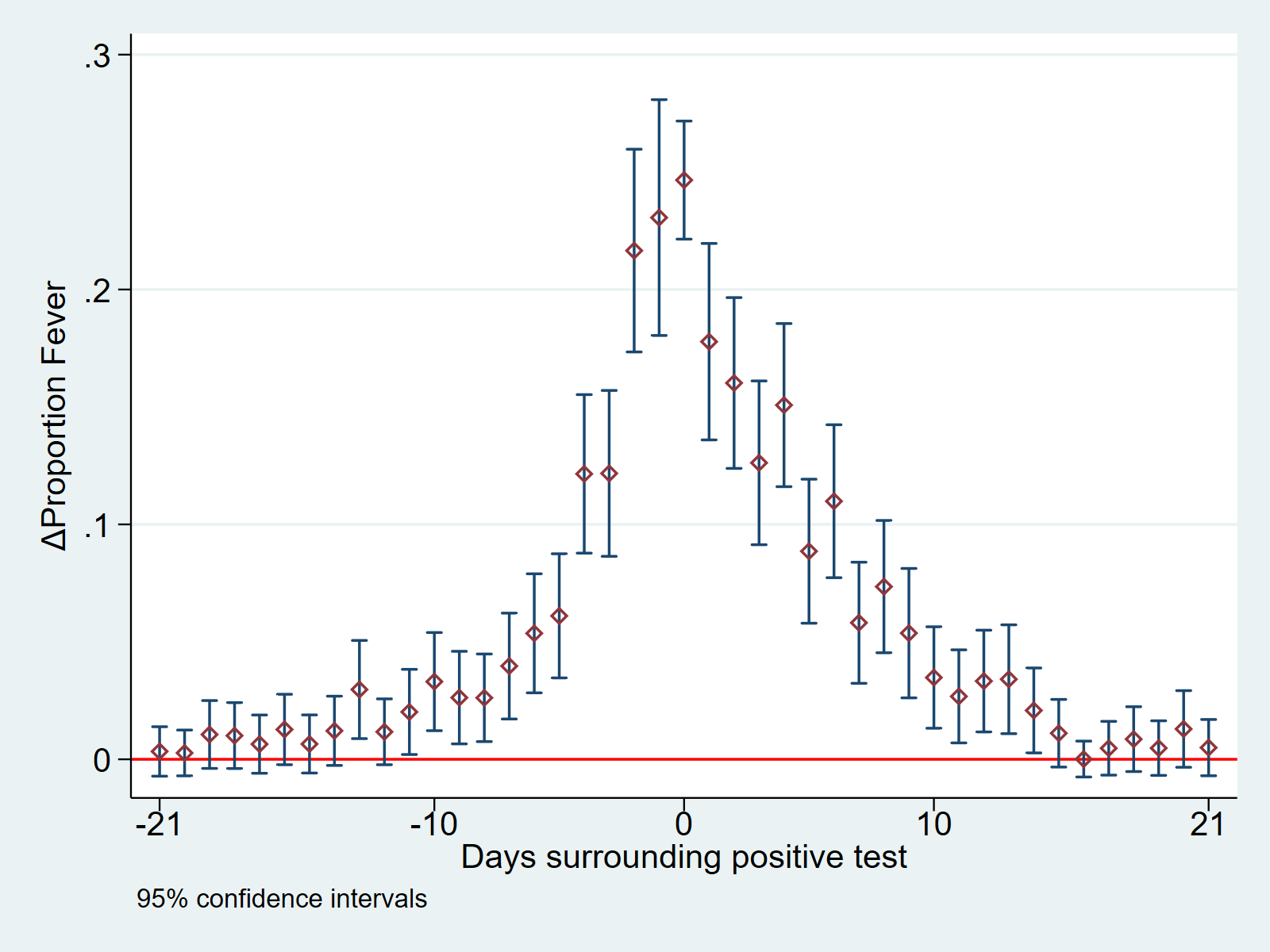

Supplement: S7 File — (ZIP) [file pone.0253566.s023.zip › sensitivity/noZHkoorts.tif]

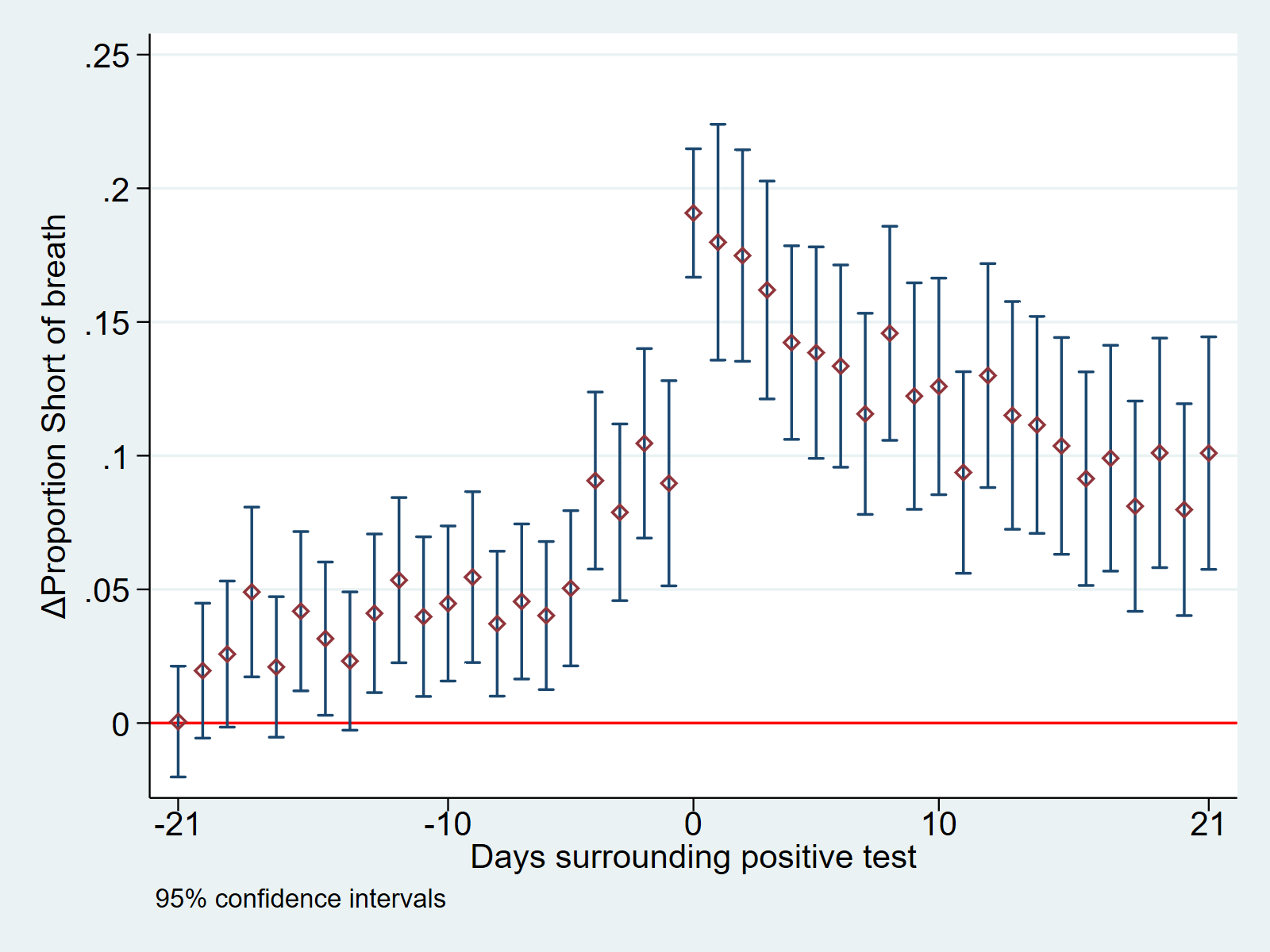

Supplement: S7 File — (ZIP) [file pone.0253566.s023.zip › sensitivity/noZHkortademig.tif]

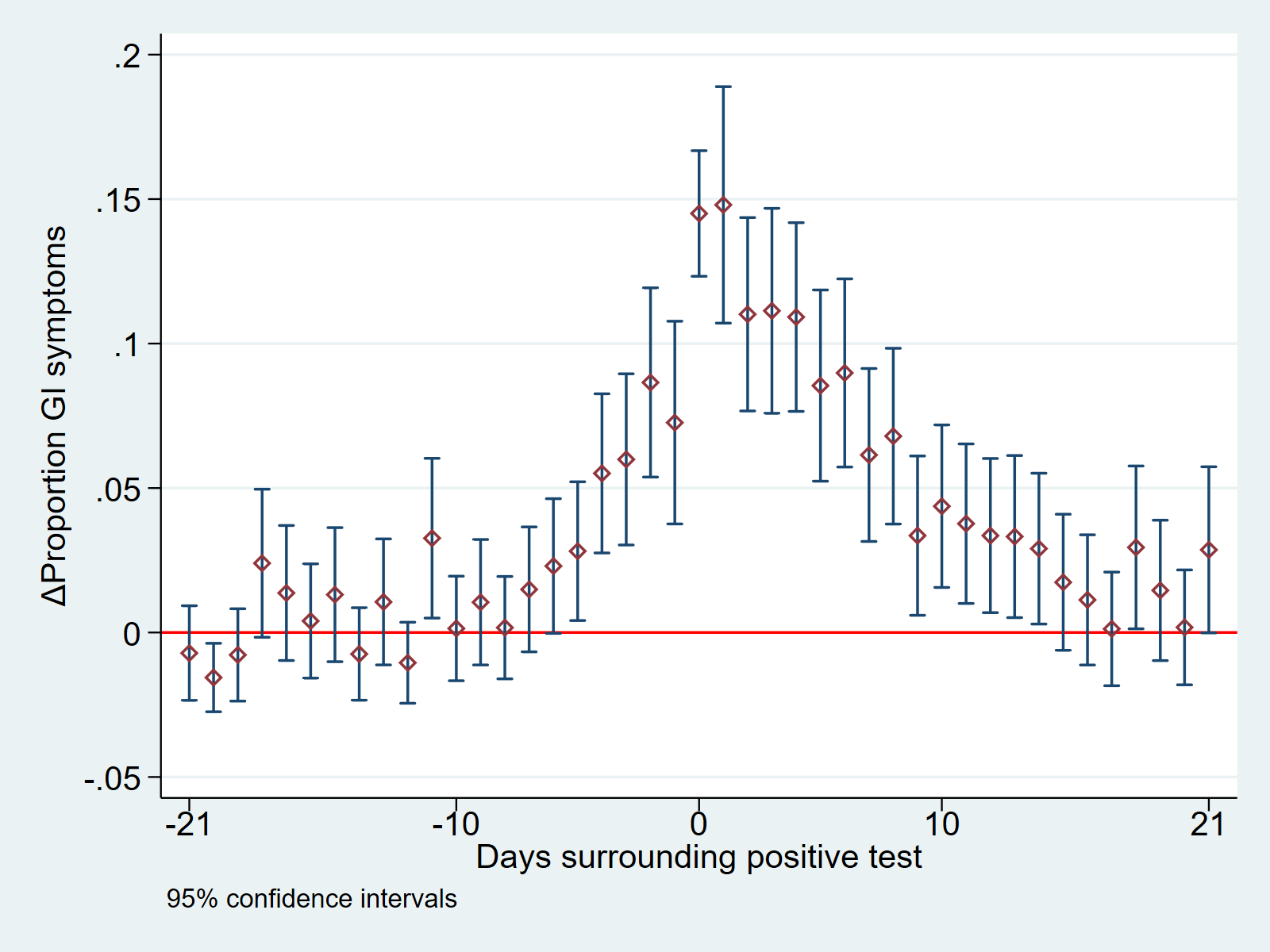

Supplement: S7 File — (ZIP) [file pone.0253566.s023.zip › sensitivity/noZHmaagdarm.tif]

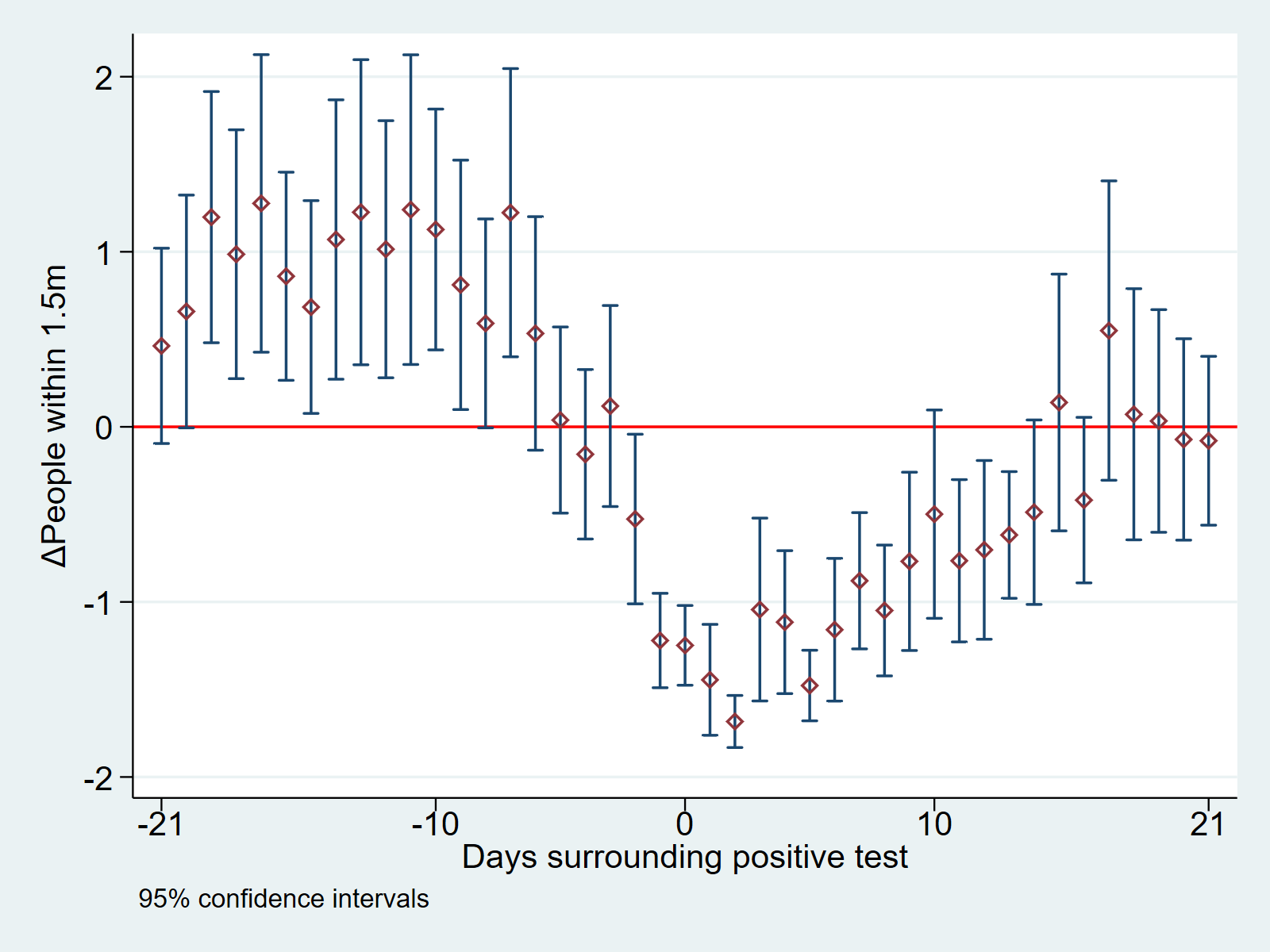

Supplement: S7 File — (ZIP) [file pone.0253566.s023.zip › sensitivity/noZHnabij.tif]

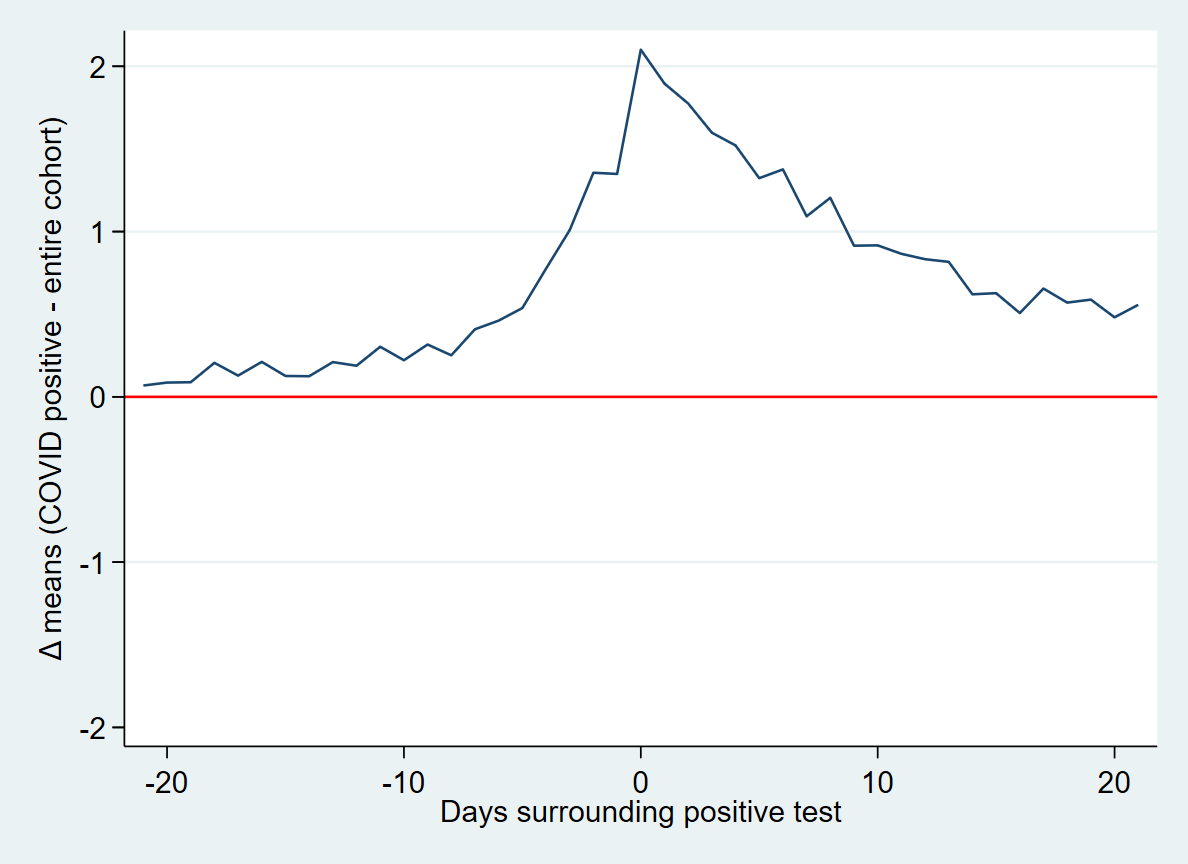

Supplement: S7 File — (ZIP) [file pone.0253566.s023.zip › sensitivity/noZHnsymptest.tif]

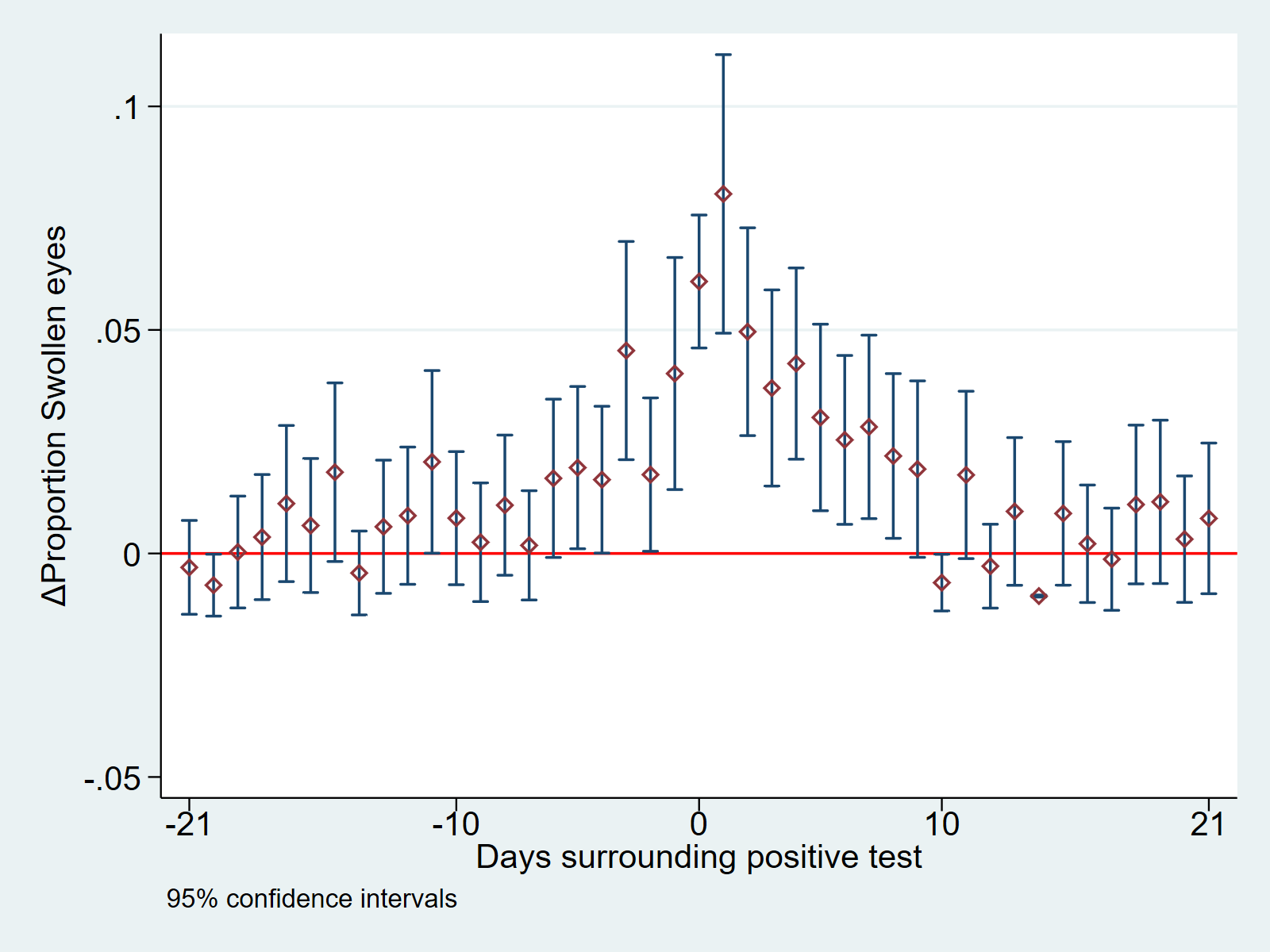

Supplement: S7 File — (ZIP) [file pone.0253566.s023.zip › sensitivity/noZHogen.tif]

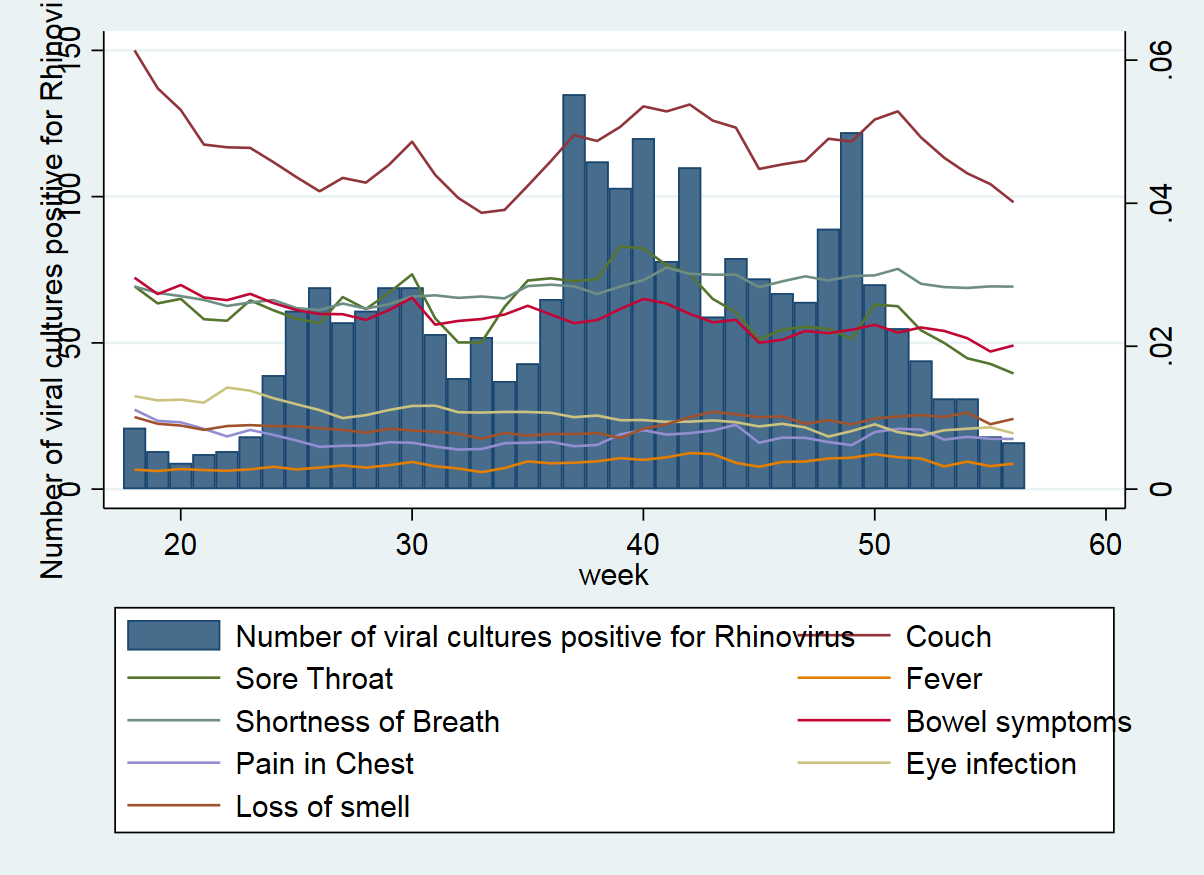

Supplement: S7 File — (ZIP) [file pone.0253566.s023.zip › sensitivity/noZHrhinovirus.tif]

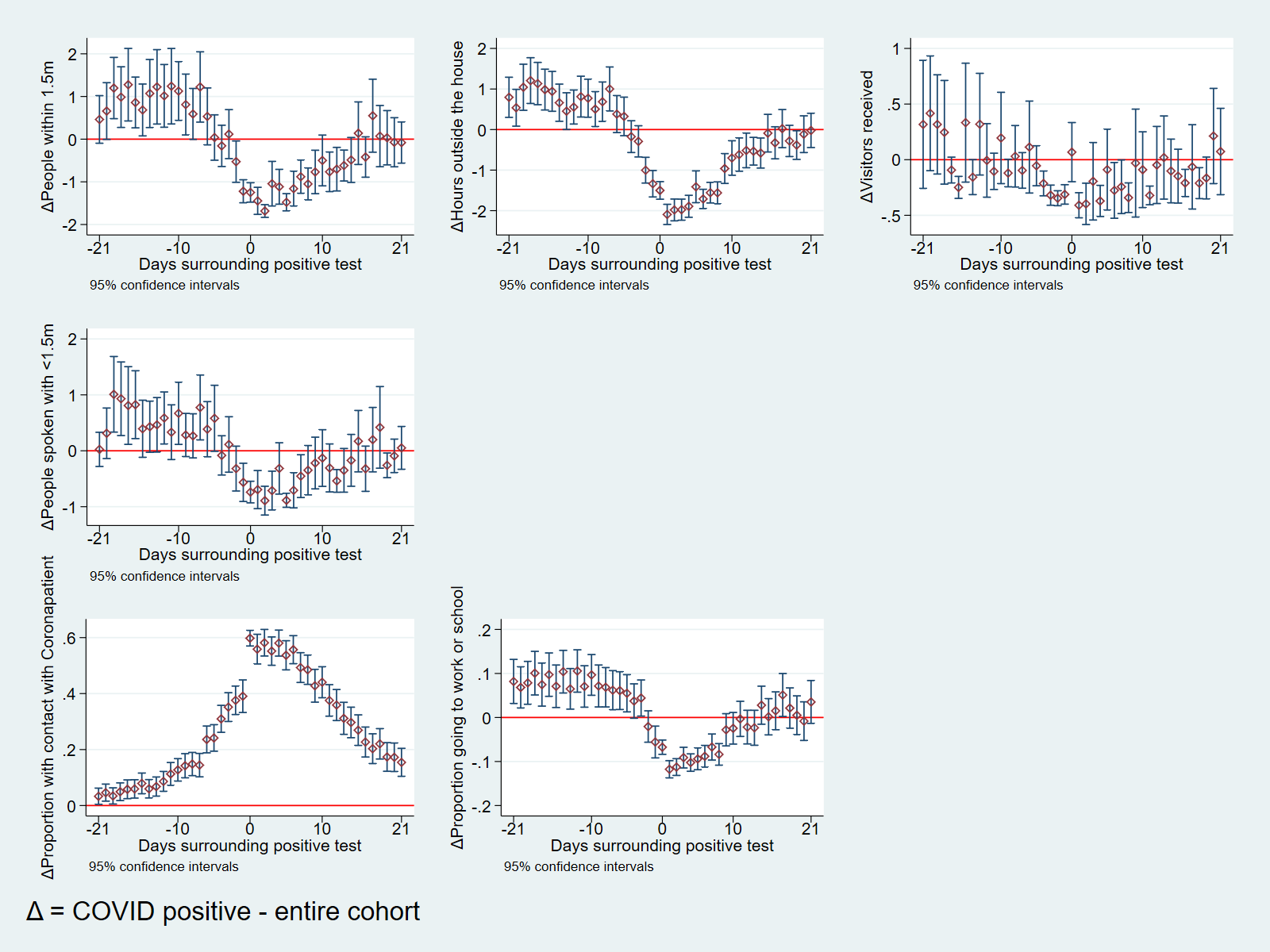

Supplement: S7 File — (ZIP) [file pone.0253566.s023.zip › sensitivity/noZHS19S25Behave.tif]

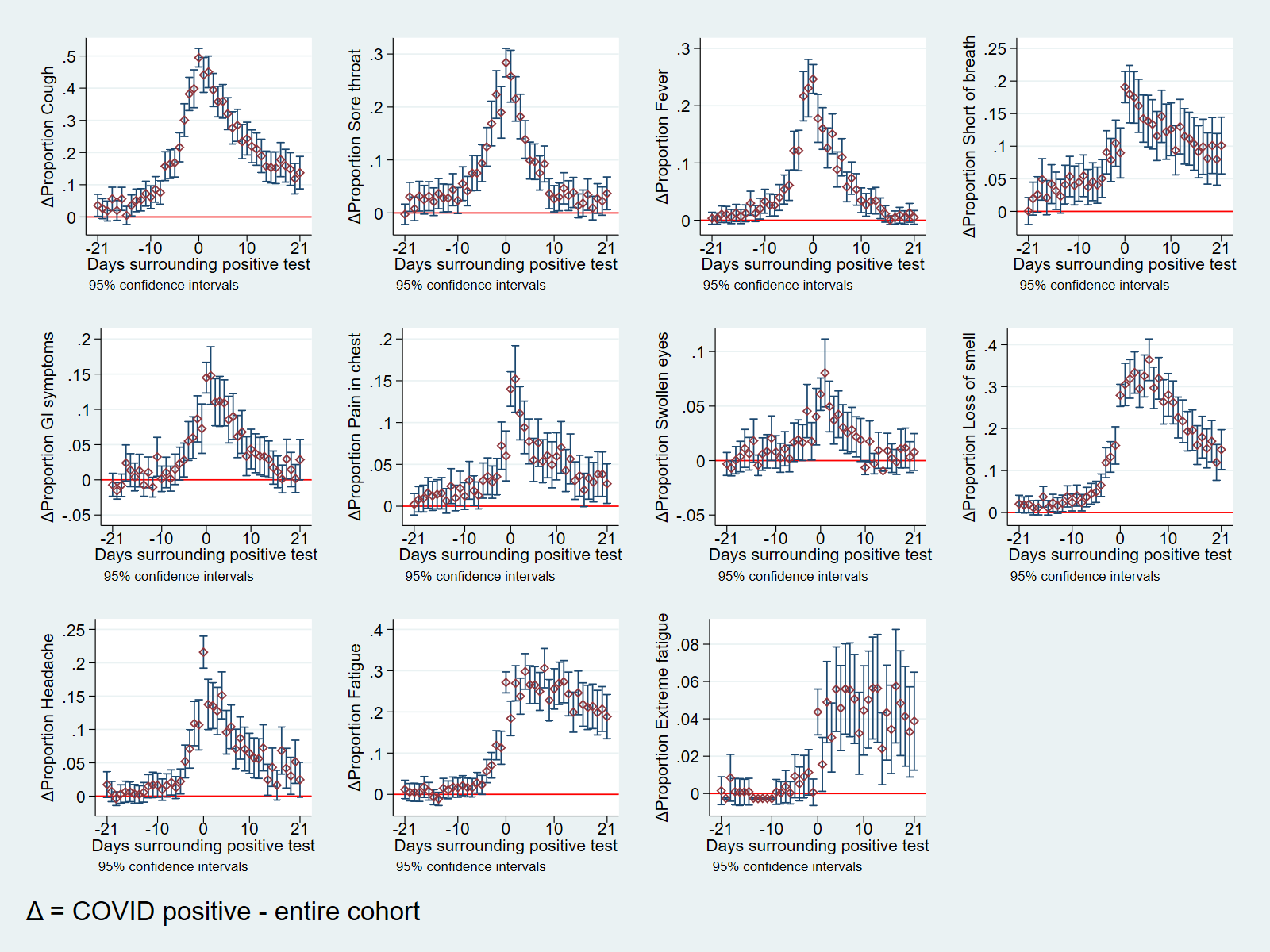

Supplement: S7 File — (ZIP) [file pone.0253566.s023.zip › sensitivity/noZHS6-S16AllSymp.tif]

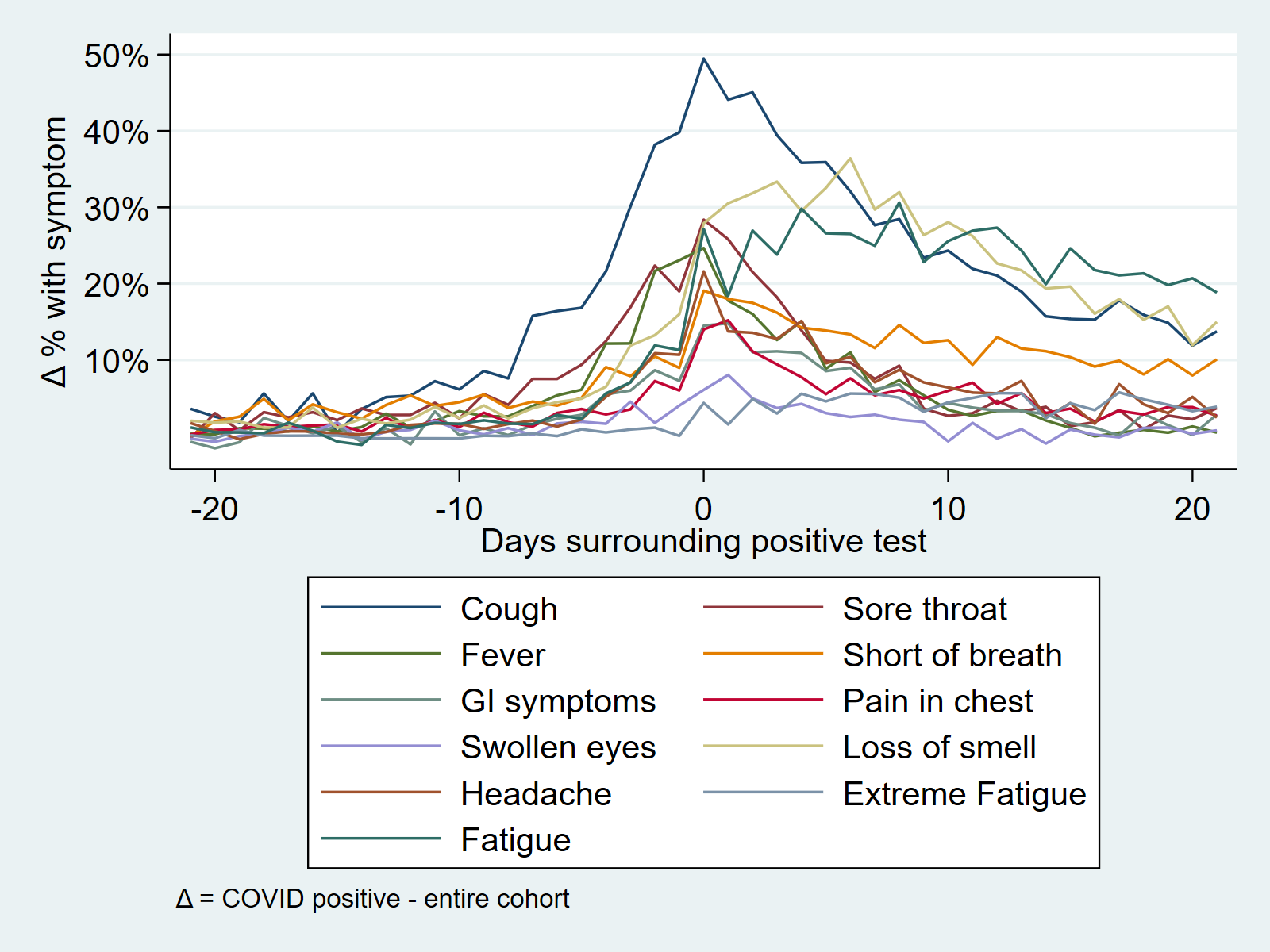

Supplement: S7 File — (ZIP) [file pone.0253566.s023.zip › sensitivity/noZHsymptomstest.tif]

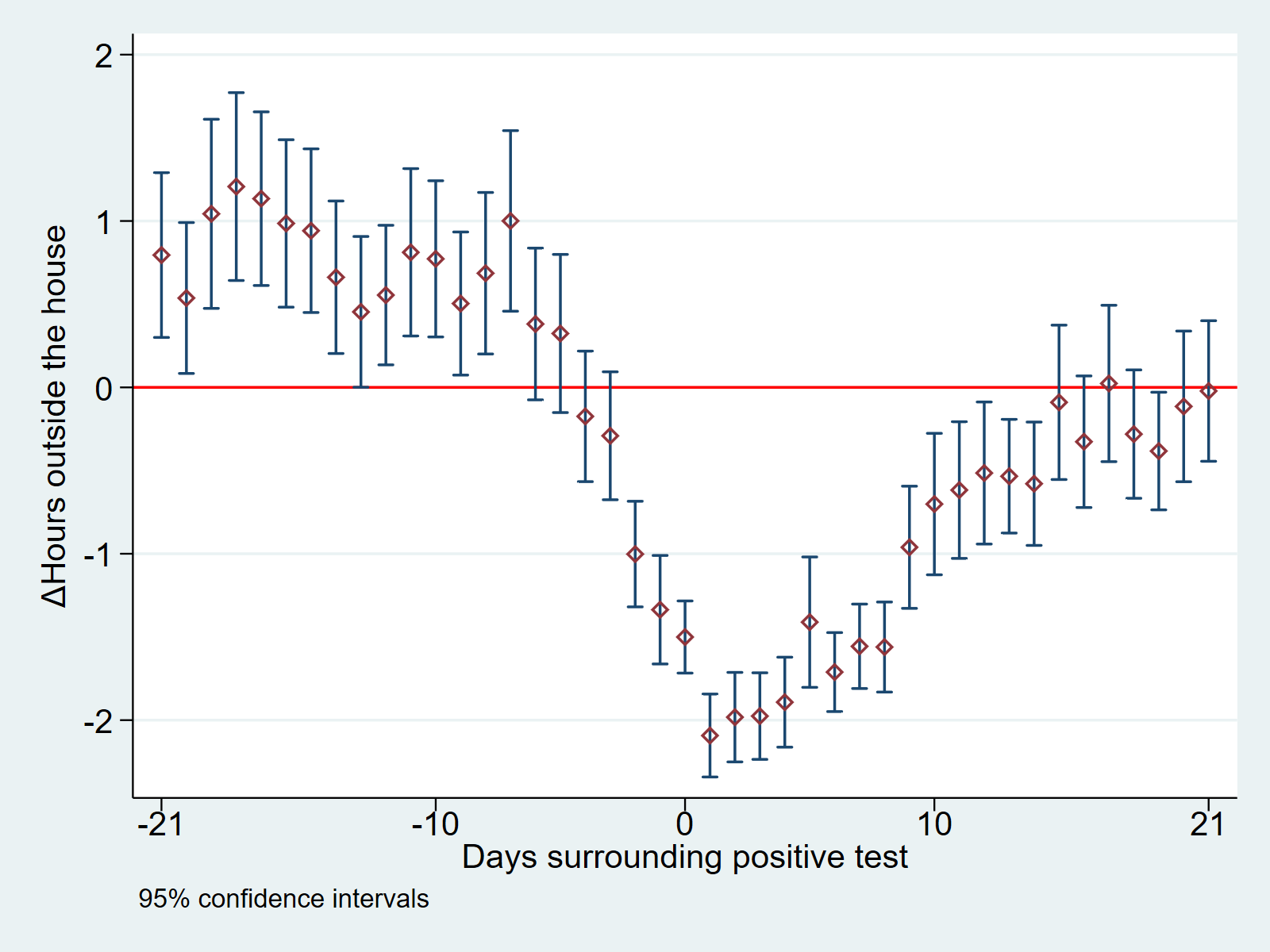

Supplement: S7 File — (ZIP) [file pone.0253566.s023.zip › sensitivity/noZHuithuis.tif]

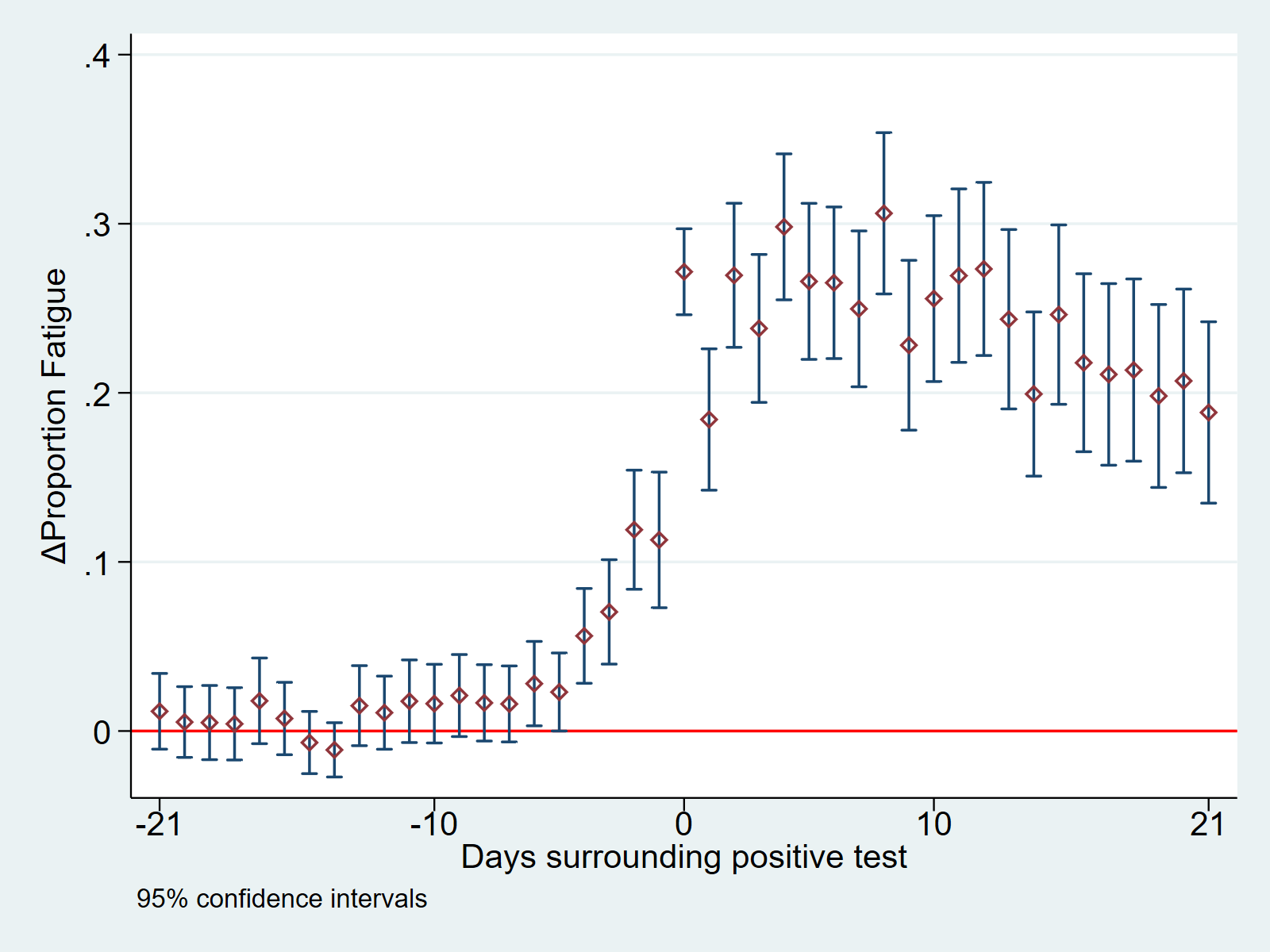

Supplement: S7 File — (ZIP) [file pone.0253566.s023.zip › sensitivity/noZHvermoeidheid.tif]

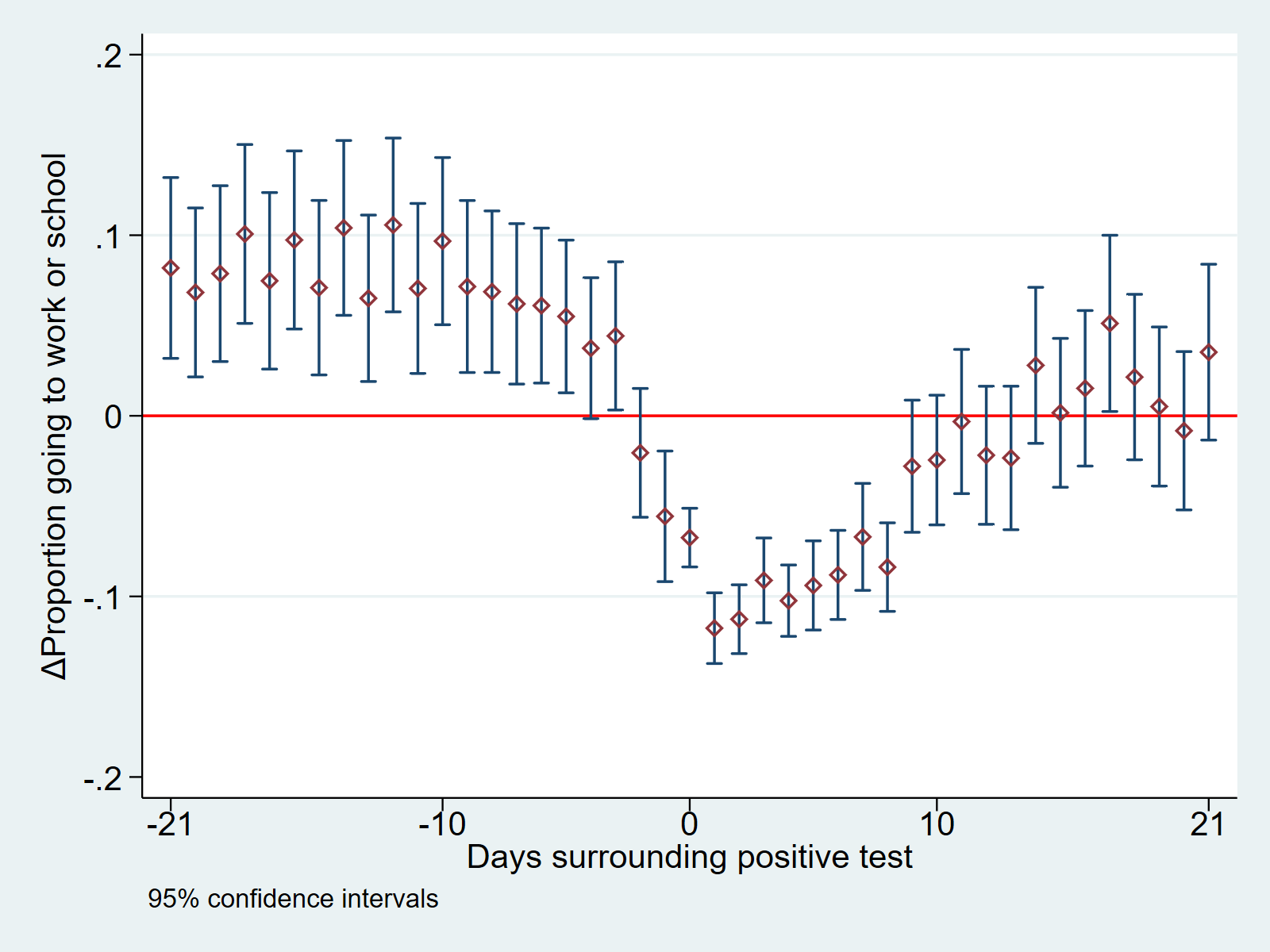

Supplement: S7 File — (ZIP) [file pone.0253566.s023.zip › sensitivity/noZHwerk.tif]

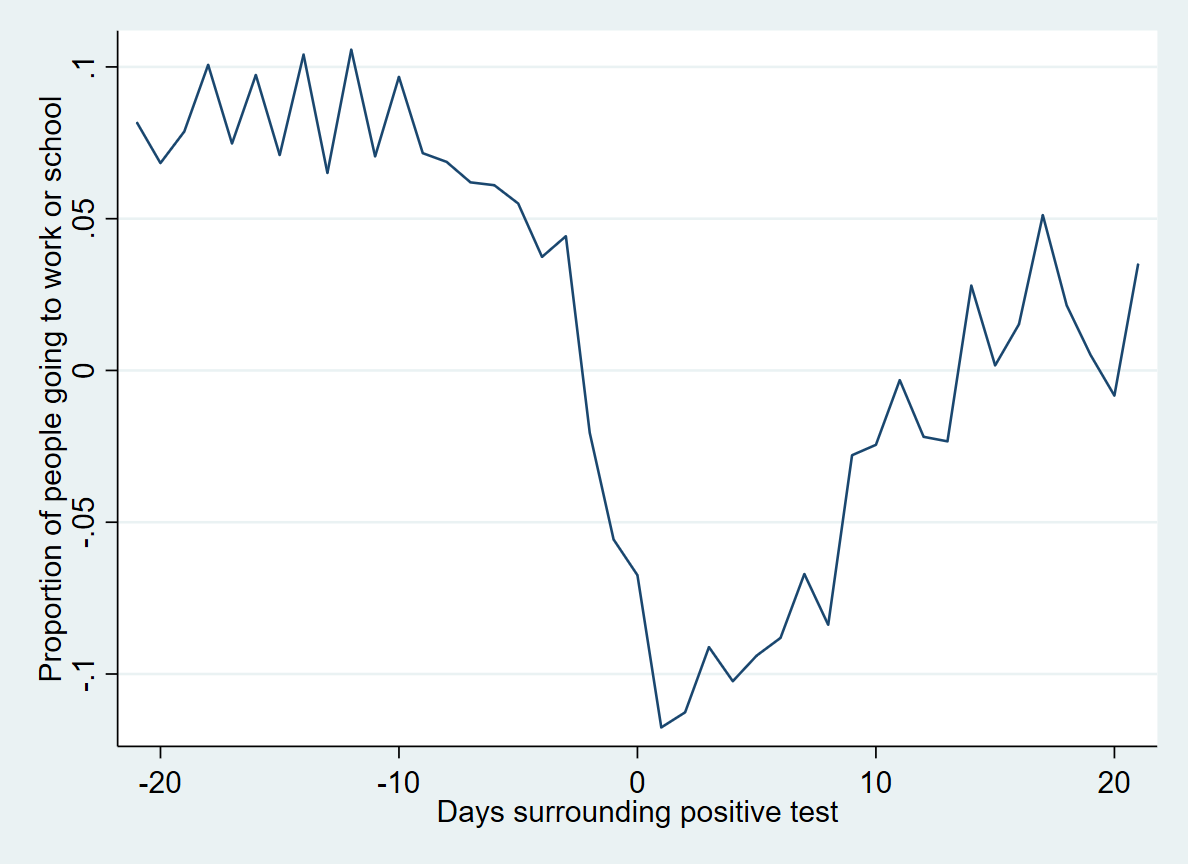

Supplement: S7 File — (ZIP) [file pone.0253566.s023.zip › sensitivity/noZHwerktest.tif]
